# Symmetry-controlled multi-gap superconductivity and higher-order topological phases of MoTe_2_

**DOI:** 10.1038/s41467-026-72368-x

**Published:** 2026-05-19

**Authors:** Sangyun Lee, Myungjun Kang, Jihyun Kim, Suyeon Cho, Duk Y. Kim, Sangmo Cheon, Tuson Park

**Affiliations:** 1 Center for Quantum Materials and Superconductivity (CQMS) and Department of Physics, Sungkyunkwan University, Suwon, South Korea; 2 Los Alamos National Laboratory, Los Alamos, NM USA; 3 Department of Physics and National High Magnetic Field Laboratory, University of Florida, Gainesville, Florida USA; 4 Department of Physics, Hanyang University, Seoul, South Korea; 5 High Pressure Research Center, Hanyang University, Seoul, South Korea; 6 Department of Physics, University of Wisconsin–Milwaukee, Milwaukee, Wisconsin USA; 7 Institute for Multiscale Matter and Systems (IMMS), Ewha Womans University, Seoul, South Korea; 8 Agency for Defense Development, Daejeon, South Korea; 9 Research Institute for Natural Science, Hanyang University, Seoul, South Korea; 10 Hanyang Institute for Quantum Science and Quantum Technology, Hanyang University, Seoul, South Korea

**Keywords:** Superconducting properties and materials, Topological matter

## Abstract

The transition-metal dichalcogenide MoTe_2_ has been proposed as an ideal platform to intertwine superconductivity with band topology, yet a key experiment—tracking how its properties evolve across a pressure-tuned structural and topological phase transition—has remained elusive. Here, we map the superconducting landscape across these high-pressure regimes from the noncentrosymmetric type-II Weyl semimetal T_*d*_ phase to the centrosymmetric $1{{{{\rm{T}}}}}^{{\prime} }$ phase using surface-sensitive soft point-contact Andreev spectroscopy combined with quantitative theoretical analysis. In the T_*d*_ phase, our spectra consistently reveal two distinct superconducting gaps that remain resolvable under an external magnetic field, implying robust and pressure-independent multi-gap superconductivity consistent with muon-spin-rotation evidence for two *s*-wave gaps at ambient pressure. In the $1{{{{\rm{T}}}}}^{{\prime} }$ phase, reached by pressure along a topological pathway that connects the Weyl to the higher-order topological phase, we observe an *s* + *p*-wave surface response whose *p*-wave component follows the *s*-wave gap in temperature and is rapidly suppressed by a magnetic field—fingerprints of proximity-induced *p*-wave pairing between a bulk *s*-wave superconducting band and second-order topological surface states. This phenomenology aligns with theoretical analysis showing that the T_*d*_ phase hosts type-II Weyl points, whereas the $1{{{{\rm{T}}}}}^{{\prime} }$ phase realizes a higher-order topological insulator arising from double-band inversion. Finally, we further propose that the resulting higher-order hinge boundary channels provide a natural route toward potential zero-energy Majorana corner modes under the observed *s* + *p*-wave proximity pairing, suggesting MoTe_2_ as an intrinsic, pressure-tunable platform for multi-gap and *s* + *p*-wave topological superconductivity.

## Abstract (web summary)

MoTe_2_ has attracted attention as a potential topological superconductor. Here, using the point-contact Andreev reflection spectroscopy, the authors reveal the landscape of superconductivity in MoTe_2_, from Weyl to higher-order topological bands, suggesting a tunable platform for exotic superconductivity and potential Majorana states.

## Introduction

Nontrivial electronic band topology and symmetry-protected superconductivity are at the forefront of condensed-matter physics and hold considerable promise for fault-tolerant quantum information processing. Paradigmatic examples such as Dirac/Weyl semimetals and higher-order topological insulators host unconventional boundary states ranging from Fermi-arc surface bands to hinge- and corner-localized modes^1,2^. When these topological bands become superconducting (SC), they can in principle give rise to Majorana excitations obeying non-Abelian statistics, offering a route toward topological quantum computation^3,4^.

**Fig. 1:**
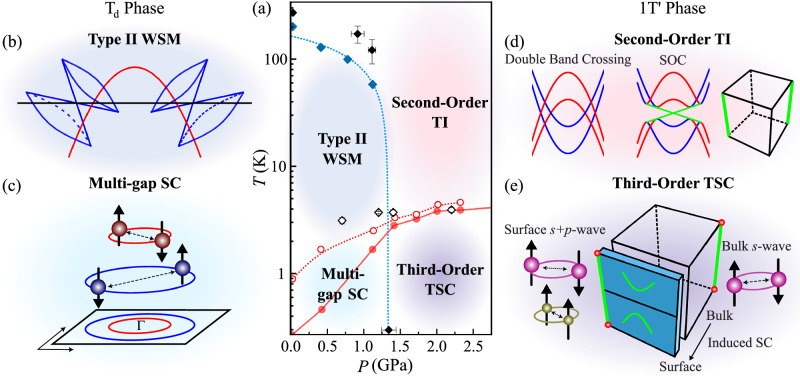
Temperature-pressure phase diagram and schematic evolution of topology and superconductivity in MoTe_2_. **a** Phase diagram of MoTe_2_ across the T_*d*_ and $1{{{{\rm{T}}}}}^{{\prime} }$ phases. Blue and black diamonds indicate the structural transition temperature (T^*^) from $1{{{{\rm{T}}}}}^{{\prime} }$ to T_*d*_ derived from resistivity and X-ray data^74,75^. Red circles mark the onset (*T*_c,onset_) and zero-resistance (*T*_zero_) SC transitions^74^, while open black diamonds show *T*_*c*_ measured by point-contact Andreev reflection, in good agreement with *T*_c,onset_. The suppression of T^*^ correlates with a sharp rise of *T*_*c*_ in the T_*d*_ phase near the putative quantum critical point (*P*_*c*_ ≈ 1.4 GPa). **b** Schematic band structure near *Γ* for the type-II Weyl semimetal, where a parabolic band (red) lies between two Weyl cones (blue). **c** Multi-gap superconductivity in the T_*d*_ phase. Below *T*_*c*_, both the parabolic band and Weyl cones in **b** develop distinct SC gaps, forming separate Cooper-pair condensates. **d** Band structure and wave-function distribution of the second-order topological insulating band. With SOC, the double band crossing (left) opens a gap, yielding a bulk insulator that hosts second-order topological boundary states (green, middle), localized at the crystal hinges or corners (right). **e** Schematic of the bulk-to-surface proximity effect. Bulk *s*-wave superconductivity induces a *p*-wave component on the helical surface states (left), forming *s *+ *p*-wave pairing on the surface (right) and gapped hinge states (green) hosting localized Majorana corner modes (red) in the proposed third-order topological superconducting phase.

Within this broader landscape, transition-metal dichalcogenides (TMDCs) have emerged as a versatile materials family in which band topology and superconductivity can coexist and be tuned by strain, doping, gating, or pressure^5–9^. MoTe_2_ is particularly appealing because it hosts a sequence of distinct topological phases within a single compound^10–19^ [Fig. 1a]. At ambient pressure, the orthorhombic and noncentrosymmetric T_*d*_ phase is a type-II Weyl semimetal with Fermi-arc surface states coexisting with a parabolic bulk band near the *Γ* point^20–24^ [Fig. 1b]. Experiments have reported a multi-gap SC response, likely originating from both the parabolic and Weyl bands [Fig. 1c], although the existence of multiple gaps in the T_*d*_ phase remains under debate^24–26^. Under higher pressure, MoTe_2_ transforms into the monoclinic, centrosymmetric $1{{{{\rm{T}}}}}^{{\prime} }$ phase, predicted to host higher-order topological bands protected by time-reversal and inversion symmetries^5,27–32^ [Fig. 1d]. In the closely related WTe_2_, Josephson interferometry and transport measurements provide compelling evidence for hinge conduction consistent with higher-order topology, highlighting the XTe_2_ family as a platform where higher-order bands are experimentally accessible^28^. This pressure-driven evolution from a type-II Weyl semimetal to a higher-order topological phase in MoTe_2_ therefore offers a unique opportunity to investigate how electronic topology and superconductivity intertwine across a symmetry-restoring transition.

From the perspective of realizing topological superconductivity, MoTe_2_ offers several unique advantages. A conventional strategy is to seek intrinsic *p*-wave superconductivity, with prominent candidates including Sr_2_RuO_4_ and Cu_*x*_Bi_2_Se_3_^33,34^. However, such states tend to be highly sensitive to disorder and are limited to a small number of candidate materials. An alternative, extrinsic route is to engineer proximity-induced *p*-wave superconductivity at the interface between a topological insulator and an *s*-wave superconductor^35,36^. This approach, however, typically requires long SC coherence lengths and precise heterostructure engineering, which significantly restricts practical implementation. Motivated by these limitations, we focus on an intermediate regime in which superconductivity and topological bands coexist within a single material. The $1{{{{\rm{T}}}}}^{{\prime} }$ phase of MoTe_2_ precisely realizes such a regime; a bulk *s*-wave SC band coexists with higher-order topological bands. Interband coupling between the bulk *s*-wave SC states and the topological bands can induce an effective *p*-wave pairing on the surface through a momentum-space proximity effect [Fig. 1e]. This mechanism provides an intrinsic route to topological superconductivity in a single compound, overcoming the drawbacks of both intrinsic and heterostructure-based implementations, analogous to the self-proximitized topological superconductivity suggested in Fe-based superconductors^37,38^.

Despite its potential importance in understanding the interplay between topology and superconductivity in MoTe_2_, experimental studies remain scarce, primarily because accessing both phases requires extremely high pressures, which is challenging for surface-sensitive techniques such as ARPES and STM. Developing alternative spectroscopic probes and controlling the relevant symmetries are, therefore, crucial for elucidating the SC properties of MoTe_2_.

Here, we systematically trace the evolution of superconductivity across the pressure-driven structural and topological transformation of MoTe_2_ under extreme pressure. Using surface-sensitive soft point-contact Andreev reflection spectroscopy (PCAR) over a wide pressure range and under various temperatures and magnetic fields, complemented by a quantitative Blonder-Tinkham-Klapwijk (BTK) analysis, we capture the transition from a multi-gap SC state in the noncentrosymmetric type-II Weyl T_*d*_ phase to a proximity-induced topological state in the centrosymmetric $1{{{{\rm{T}}}}}^{{\prime} }$ phase. In the T_*d*_ phase, our spectra consistently reveal two well-resolved SC gaps, implying robust and pressure-independent multi-gap superconductivity rooted in the type-II Weyl semimetal state; a self-consistent multi-gap analysis incorporating interband coupling quantitatively reproduces the experimental behavior and corroborates the two-gap nature reported by muon-spin-rotation measurements^20–26^. In the $1{{{{\rm{T}}}}}^{{\prime} }$ phase, accessed upon further compression, a sharp conductance peak emerges near zero bias, indicative of a surface *p*-wave component induced through proximity coupling between bulk *s*-wave superconductivity and helical Dirac surface states derived from a second-order topological insulating (SOTI) band protected by time-reversal and inversion symmetries. The similar temperature and magnetic-field dependence of both gaps supports this proximity-induced *s *+ *p*-wave scenario, while the surface *p*-wave component is fragile—readily suppressed by magnetic fields or the loss of inversion symmetry as pressure is reduced—reflecting the correlation with the bulk *s*-wave order. Furthermore, theoretical analysis based on a Bogoliubov–de Gennes (BdG) Hamiltonian suggests that the SOTI hinge states can provide a route to corner-localized Majorana zero modes under appropriate *s *+ *p*-wave proximity pairing, pointing to a possible third-order topological superconducting (TOTSC) regime. Collectively, these findings imply MoTe_2_ as a symmetry-controlled and pressure-tunable platform in which multi-gap and topological superconductivity can be accessed, suggesting an alternative route toward realizing Majorana physics in TMDCs.

## Results

**Fig. 2:**
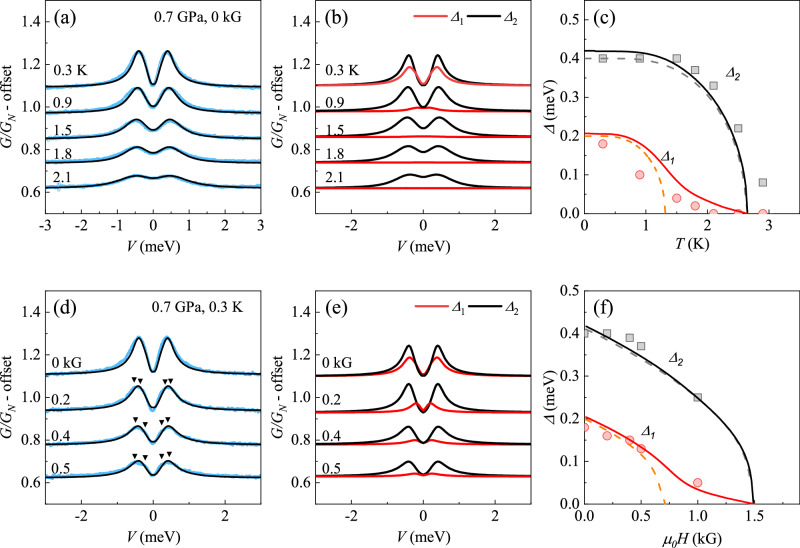
Multi-gap superconductivity in T_*d*_-MoTe_2_ at 0.7 GPa. **a** Temperature-dependent normalized conductance spectra. Symbols show experimental data, and solid lines represent two-gap *s*-wave BTK fits. **b** Decomposition of the BTK fit into contributions from the larger gap Δ_2_ (black) and the smaller gap Δ_1_ (red). Curves in **a**, **b** are vertically offset for clarity. **c** Temperature dependence of the extracted gaps Δ_1,2_(*T*) from BTK analysis (symbols) compared with coupled-gap calculations. Solid lines include finite interband coupling, whereas dashed lines show the decoupled limit without coupling. **d** Field-dependent normalized conductance spectra at 0.7 GPa and 0.3 K with corresponding BTK fits (solid lines). Arrows mark the two gap-edge features and their evolution with the magnetic field. **e** Decomposition of the field-dependent spectra into Δ_2_ (black) and Δ_1_ (red) components. Curves in **d**, **e** are vertically offset for clarity. **f** Magnetic-field dependence of the gaps Δ_1,2_(*H*) extracted from BTK analysis (symbols), compared with calculations with (solid lines) and without (dashed lines) coupling.

We systematically examine the multi-gap nature of superconductivity in orthorhombic T_*d*_-MoTe_2_ under pressure across a wide range of experimental conditions using PCAR. Figure 2a shows the temperature-dependent evolution of the PCAR spectra at 0.7 GPa together with fits based on the BTK formalism^39,40^ [see Secs. S1.1.1–S1.1.3 for analysis details]. Below *T*_*c*_, the spectra develop two well-resolved gap-edge features that can be reproduced when two SC gaps are included in the BTK analysis. The individual gap contributions are displayed in Fig. 2b, and the extracted gap values (symbols) are summarized in Fig. 2c. Notably, the smaller gap exhibits a pronounced tail as *T* approaches *T*_*c*_, consistent with finite coupling in a coupled two-gap system [Sec. S2]. This characteristic is shown for the BTK analysis (symbols) as well as the theoretical calculations (solid lines) and does not exist when there is no interband coupling (dashed lines), within a type-II Weyl-semimetal-derived SC model. The resulting BCS gap ratios are comparable to those reported for other multi-gap superconductors [Sec. S1.2.6]^41–46^. Details of the theoretical model and the multi-gap analysis are provided in Secs. S2.1, S2.2, respectively.

The magnetic field dependence of the PCAR spectra further supports the multi-gap scenario. The two-gap character becomes apparent under fields up to 0.5 kG, as indicated by the arrows in Fig. 2d, and the evolution of the two gaps with respect to a magnetic field is shown in Fig. 2e. The extracted gap magnitudes Δ_*i*_(*H*) from the BTK analysis are summarized in Fig. 2f (symbols). For comparison, the solid and dashed lines show calculations from multi-gap equations with and without interband coupling, respectively [Sec. S2], within the same model used in Fig. 2c. Without interband coupling (dashed lines), the smaller gap is rapidly suppressed with increasing field and closes near its intrinsic pair-breaking scale. In contrast, finite interband coupling (solid lines) enforces a cooperative evolution of the two gaps, allowing the smaller gap to persist to higher fields by being sustained by the larger gap. This coupled-gap behavior reproduces the experimentally observed long-tailed suppression of the smaller gap as *H* goes to *H*_*c*2_, which is absent in the decoupled limit.

Although the temperature-dependent PCAR spectra in Figs. 2a, S1a of Sec. S1.2.1 can be fitted reasonably by a single-gap *s*-wave model, the field-dependent spectra are most clearly and consistently described by two-gap BTK fits below *H*_*c*2_, as shown in Fig. 2d–f. Details of the fitting procedures and parameters are provided in Sec. S1.2.2. Furthermore, the temperature dependence of the upper critical field *H*_*c*2_(*T*) exhibits a characteristic negative curvature near *T*_*c*_ [Fig. S2], which is well captured by a two-band Werthamer-Helfand-Hohenberg (WHH) model, but is difficult to reconcile with single-gap superconductivity [Sec. S1.2.3]. This behavior persists at an elevated pressure of 1.2 GPa, as shown in Fig. S3, underlining the robustness of the two-gap structure against pressure [see Secs. S1.2.4–S1.2.6 for detailed data, fitting parameters, and field dependence]. Taken together, these observations are consistent with previous muon-spin-rotation measurements at ambient pressure, which reported two *s*-wave gaps in T_*d*_-MoTe_2_^26^.

The origin of the two distinct SC gaps can be traced to the multiband electronic structure of the T_*d*_ phase. In the normal state, MoTe_2_ hosts both quasi-parabolic bands and Weyl-derived bands near the Fermi level^20–24^. The orthorhombic T_*d*_ structure lacks inversion symmetry and hosts multiple Weyl nodes arising from strongly spin-orbit-coupled electron and hole pockets near the Fermi energy. This intricate multiband Fermi-surface topology naturally favors multi-gap superconductivity with finite interband pairing interactions, consistent with our PCAR analysis and previous bulk probes^24–26^. Our results therefore indicate that superconductivity in the T_*d*_ phase emerges from a multiband electronic environment intrinsic to the Weyl semimetal state of MoTe_2_, and remains robust under both ambient and moderate pressures.

**Fig. 3:**
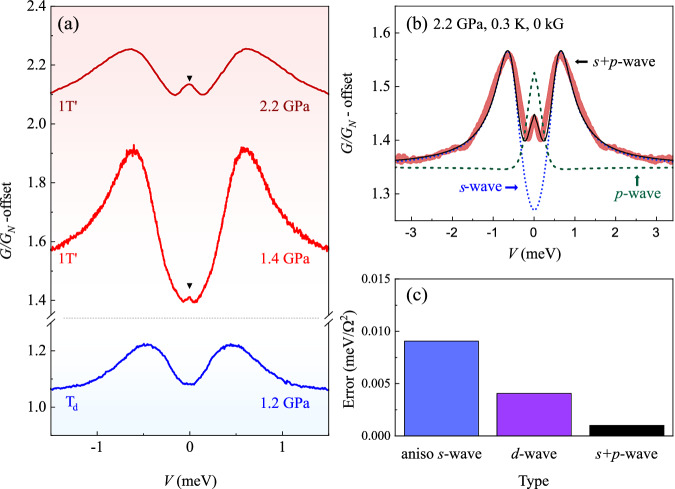
Pressure-tuned emergence of *s* + *p*-wave superconductivity in MoTe_2_. **a** Evolution of the normalized differential conductance from the T_*d*_ (1.2 GPa) to the $1{{{{\rm{T}}}}}^{{\prime} }$ (1.4 and 2.2 GPa) phases at 0.3 K. The black triangle marks a conductance peak near zero-bias, which appears only in the $1{{{{\rm{T}}}}}^{{\prime} }$ phase. **b** BTK fits for the normalized conductance measured at 2.2 GPa. Blue, green, and black curves represent the *s*-, *p*-, and combined *s* + *p*-wave SC states, respectively, derived from two-gap BTK modeling. **c** Total fitting error, estimated as the difference between the experimental data and BTK calculations, which highlights the agreement of the *s* + *p*-wave model.

As pressure is increased, MoTe_2_ undergoes a structural transition from the orthorhombic T_*d*_ phase to the monoclinic $1{{{{\rm{T}}}}}^{{\prime} }$ phase while SC remains below *T*_*c*_ [Fig. 1a]. The evolution of the soft point-contact spectra at 0.3 K from 1.2 to 2.2 GPa shows that, upon entering the $1{{{{\rm{T}}}}}^{{\prime} }$ phase, a sharp conductance peak develops near zero-bias within the double-peak structure characteristic of the SC gap [Fig. 3a]. This feature is well captured by the BTK fit in Fig. 3b, which displays the normalized conductance at 0.3 K and 2.2 GPa, clearly resolving the *s*- and *p*-wave contributions with corresponding gap magnitudes Δ_*s*_ = 0.6 meV and Δ_*p*_ = 0.4 meV, indicating that the *p*-wave channel makes a substantial and reproducible contribution to the fits rather than being a numerical artifact.

Within the class of BTK models we have considered, the PCAR spectra in the $1{{{{\rm{T}}}}}^{{\prime} }$ phase are most consistently captured by an *s* + *p*-wave gap [Fig. 3c]. Deviations between the fits and the PCAR data in Figs. 3c, S4 are substantially smaller for the *s* + *p*-wave model than for anisotropic *s*-wave or *d*-wave models, supporting the *s* + *p*-wave scenario in the $1{{{{\rm{T}}}}}^{{\prime} }$ phase [see Sec. S1.3.1 for detailed analysis]. Moreover, a fully gapped isotropic *s*-wave state fails to reproduce the zero-bias peak [Fig. S5a; Sec. S1.3.2], and an anisotropic *s*-wave scenario likewise cannot account for the data [Fig. S6a, b; Sec. S1.3.3]. While pure *p*- or *d*-wave single-gap models can, in principle, generate a zero-bias peak through Andreev bound states at finely tuned interface angles^47,48^, the specific angles required [*α* = *π*/1.35 for *p*-wave and *α* = *π*/14 for *d*-wave; Figs. S5b, S6c, d] are incompatible with our experimental geometry, which corresponds to a normal-incidence configuration (*α* = 0). Furthermore, *d*-wave pairing is disfavored because the monoclinic symmetry of $1{{{{\rm{T}}}}}^{{\prime} }$-MoTe_2_ differs from the tetragonal lattices in which conventional *d*-wave states are typically stabilized^49^. Thus, the pressure evolution of the PCAR spectra and the systematic failure of alternative models point to an unconventional SC state in $1{{{{\rm{T}}}}}^{{\prime} }$-MoTe_2_ with a substantial *p*-wave component, most naturally realized as an *s* + *p*-wave gap.

**Fig. 4:**
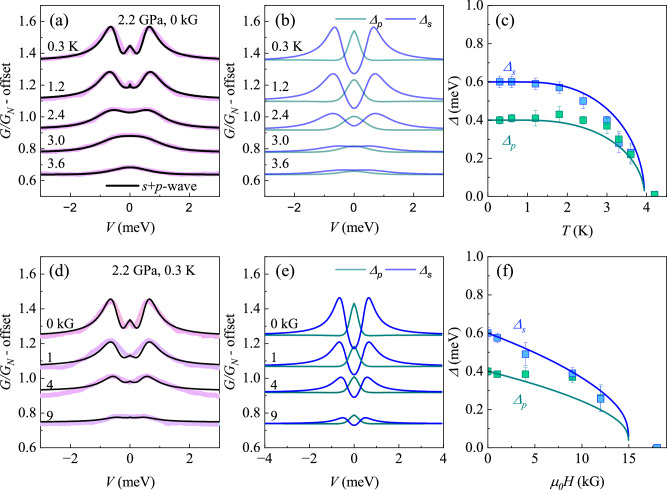
Surface *s* + *p*-wave superconductivity in $1{{{{\rm{T}}}}}^{{\prime} }$-MoTe_2_ at 2.2 GPa. **a** Temperature-dependent normalized differential conductance spectra of MoTe_2_ at 2.2 GPa. Colored curves show experimental data, while black curves represent extended BTK fits using an *s* + *p*-wave model. **b** Decomposition of the BTK fits in **a** into the *s*-wave (blue) and *p*-wave (green) components. Curves in **a**, **b** are vertically offset for clarity. **c** Temperature dependence of the extracted gaps Δ_*s*_(*T*) and Δ_*p*_(*T*) (symbols) compared with theoretical calculations including interband coupling (solid lines). **d** Field-dependent normalized conductance spectra at 0.3 K (symbols) with BTK fits (solid lines), showing a progressive suppression of the single peak near zero-bias without observable splitting. **e** Decomposition of the field-dependent spectra in **d** into the *s*- and *p*-wave components. Curves in **d**, **e** are vertically offset for clarity. **f** Magnetic-field dependence of Δ_*s*_(*H*) and Δ_*p*_(*H*) (symbols), compared with theoretical calculations (solid lines).

We next examine the properties and possible origin of the emergent *p*-wave component in the $1{{{{\rm{T}}}}}^{{\prime} }$ phase. As schematically illustrated in Fig. 1e, this component can be interpreted as a surface response induced via proximity to the bulk *s*-wave condensate, implying coupled pairing channels. Figure 4a shows the temperature-dependent PCAR spectra at 2.2 GPa, which are quantitatively captured by an *s* + *p*-wave BTK analysis in Fig. 4b. The extracted gap amplitudes (symbols) and corresponding calculations for a proximity-induced surface *s* + *p*-wave state (solid lines) are summarized in Fig. 4c, showing that the *s*- and *p*-wave components decrease in tandem with increasing temperature and vanish at the same *T*_*c*_. This parallel evolution supports a proximity-induced *p*-wave component tied to the bulk *s*-wave order parameter.

Moreover, the *s*- and *p*-wave SC gaps also exhibit similar magnetic-field dependence. The field-dependent PCAR spectra at 2.2 GPa and 0.3 K are well captured by a combined *s* + *p*-wave BTK model, which allows us to separate the two contributions [Fig. 4d, e; see Sec. S1.3.4 for fitting parameters]. Both gaps are gradually suppressed as the field increases, and the extracted Δ_*s*_(*H*) and Δ_*p*_(*H*) decrease monotonically and become nearly indistinguishable above 10 kG [Fig. 4f]. This evolution is quantitatively reproduced by our theoretical calculations (solid lines) incorporating coupling between the *s*- and *p*-wave pairing channels [Fig. 4f], indicating that the two components are not independent but form a coupled order parameter approaching a common pair-breaking field. In contrast, if the *p*-wave component originated from an intrinsic spin-triplet odd-parity SC channel, it would not necessarily be expected to track the magnetic-field suppression of the *s*-wave gap so closely; instead, we observe that it collapses together with the *s*-wave gap as *H* goes to *H*_*c*2_, reinforcing its proximity-induced character. Together with the temperature dependence, these results indicate that the *p*-wave feature is strongly tied to the underlying *s*-wave pairing, rather than arising from an independent SC channel.

Symmetry considerations provide a natural framework for the emergence of the *p*-wave component. The single peak near zero-bias observed in the inversion-symmetric $1{{{{\rm{T}}}}}^{{\prime} }$ phase is absent in the inversion-symmetry-broken T_*d*_ phase [Fig. 3a]. Moreover, the *p*-wave component is rapidly suppressed by an applied magnetic field, indicating its sensitivity to time-reversal-symmetry breaking [Fig. 4d–f]. These trends suggest that the symmetry setting of the $1{{{{\rm{T}}}}}^{{\prime} }$ phase—bulk inversion symmetry and time-reversal symmetry, combined with surface inversion breaking in the presence of strong spin–orbit coupling—is closely tied to the emergence of the *p*-wave response, consistent with the theoretical analysis presented below.

Before turning to our theoretical analysis, we evaluate the origin of the single peak near zero-bias in Figs. 3, 4 by excluding extrinsic mechanisms that could mimic a *p*-wave response. One possible scenario is a heating-induced thermal regime, in which local Joule heating and critical-current effects can generate a broad, strongly field-sensitive anomaly^50,51^ [Fig. S7]. However, the magnetic-field-dependent PCAR spectra at 2.2 GPa and 0.3 K shown in Fig. 4d exhibit none of these thermal signatures, and the zero-bias conductance does not follow the expected *G*(0, *T*) ∝ 1/*ρ*(*T*) scaling, arguing against a heating-induced origin [Sec. S1.4.1]. A second possibility is that a single peak near zero-bias arises within a two-gap *s*-wave scenario when one gap is extremely small. Such a scenario would require an extreme gap disparity—the larger gap must exceed the smaller one by at least a factor of seven at 3.3 K [see Fig. S8 and Sec. S1.4.2 for a detailed argument]. At 2.2 GPa, by contrast, the extracted *p*-wave gap is approximately 75% of the *s*-wave gap, well outside this regime [Fig. 4c]. Given the small gap ratio and the low measurement temperature (0.3 K), a purely two-gap *s*-wave model cannot account for the observed zero-bias peak.

Another scenario involves in-gap bound states generated by magnetic impurities, which may produce symmetric or asymmetric double peaks and, under specific conditions, a single zero-bias peak^52–55^. Such impurity-induced peaks are typically expected to split under an applied magnetic field due to the Zeeman effect. In contrast, the peak at 2.2 GPa remains unsplit and is only gradually suppressed across the entire field range, as shown in Fig. 4d, e, disfavoring a magnetic-impurity origin. Moreover, impurity-related or other extrinsic effects would not be expected to track the structural phase boundary. Consistent with this, Fig. 3a shows no peak near zero-bias throughout the T_*d*_ phase, whereas it appears only after transitioning into the $1{{{{\rm{T}}}}}^{{\prime} }$ phase [Sec. S1.4.3]. This pronounced pressure dependence, together with the reproducibility of the spectra in independent samples, is incompatible with impurity-driven or sample-specific scenarios and instead points to an intrinsic SC mechanism tied to the $1{{{{\rm{T}}}}}^{{\prime} }$ phase. We note that angle-resolved PCAR under high-pressure measurements would offer a more stringent test of the pairing symmetry.

We now theoretically investigate the SC gaps of the T_*d*_ and $1{{{{\rm{T}}}}}^{{\prime} }$ phases below *T*_*c*_. A high-symmetry parent phase, T_0_, has been proposed to evolve into the noncentrosymmetric T_*d*_ phase via inversion-symmetry breaking, or into the centrosymmetric $1{{{{\rm{T}}}}}^{{\prime} }$ phase via a shear-mode distortion^22,56^ [see Secs. S2.1, S3.1 for model Hamiltonians]. In the T_0_ phase, the low-energy band structure near the *Γ* point consists of a parabolic band and a Dirac-like cone, with PT symmetry constraining the degeneracies. When inversion symmetry is broken, and the system enters the T_*d*_ phase, the Dirac-like cone splits into type-II Weyl cones located near the Fermi level, which coexist with the parabolic band [Fig. 1b]. Based on this electronic structure, we construct an SC Hamiltonian to rationalize the observed gap structure, in which both the parabolic band and the Weyl sectors acquire gaps below *T*_*c*_. We derive coupled two-gap BCS self-consistency equations from the mean-field theory and solve them numerically; a finite interband coupling is essential to reproduce the experimentally observed persistence of the smaller gap toward *T*_*c*_ [see Methods and Sec. S2 for detailed analysis]. In the following, we contrast this multi-gap pairing framework in the T_*d*_ phase with the centrosymmetric $1{{{{\rm{T}}}}}^{{\prime} }$ phase, where the shear-distorted band topology gives rise to higher-order (hinge) boundary states that can host a proximity-induced *p*-wave component.

**Fig. 5:**
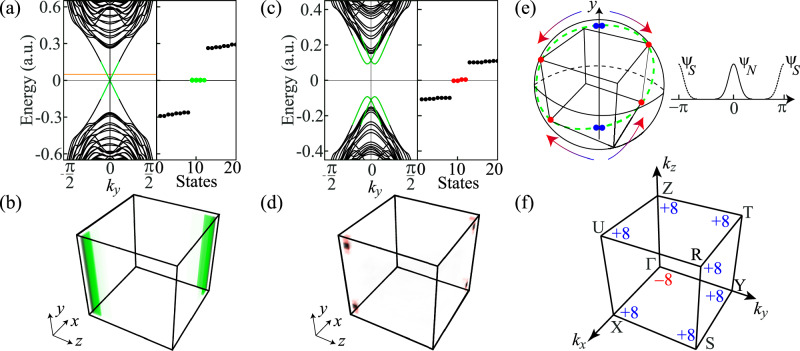
Theoretical analysis of the emergence of Majorana corner states. **a** (Left) Band structures of the finite tight-binding Hamiltonian with 15 × 15 unit cells in the *xz* plane and periodic in *y* (black) for the $1{{{{\rm{T}}}}}^{{\prime} }$ phase. The analytic hinge dispersion (green) overlaps with the numerical result; the orange line marks *μ *= 0.05. (Right) Energy eigenvalues at *k*_*y*_ = 0. **b** Wavefunction distributions of zero-energy hinge states. Darker color indicates a higher density. **c** (Left) Band structures for the SC phase with 15 × 15 unit cells (black), where the analytic hinge dispersion (green) coincides with the numerical result. (Right) Eigenvalues of the 15 × 15 × 15 lattice showing four nearly zero-energy states. **d** Real-space wavefunction distributions of the nearly zero-energy states. **e** (Left) Schematics of Majorana zero-energy states; pole states (blue) in spherical geometry move to corner states (red) along the dotted green line in cubic geometry. (Right) Localized Majorana wavefunctions along a hinge through the poles, with north (*Ψ*_*N*_, solid) and south (*Ψ*_*S*_, dashed) states well-localized at *θ* = 0 and *π*. **f** Parity eigenvalues of occupied states at TRIM points. The parameters are given in Methods.

To describe the $1{{{{\rm{T}}}}}^{{\prime} }$ phase, we adopt a minimal model capturing the low-energy effective theory in which the effect of the shear distortion is encoded through SOC and symmetry-allowed mass terms^5,27^. The SOC gaps out the Dirac-like cone of the T_0_ phase, and the additional mass terms drive a double band inversion, realizing an SOTI band^5,27,32^, as illustrated in Fig. 1d. As shown in Fig. 5a, this SOTI band structure supports surface states that consist of two helical Dirac cones with spin-momentum locking, which are gapped by symmetry-allowed mass terms^5^ [see Sec. S3.1.3 for analytical details]. In a finite geometry, these surface Dirac cones, in turn, host one-dimensional gapless hinge states, a hallmark of higher-order topology^2^ [Fig. 5b]. Additional details—including the lattice Hamiltonian, symmetry analysis, and numerical and analytical results—are provided in Sec. S3.1.

Motivated by the coexistence of a bulk *s*-wave SC band and higher-order band topology in $1{{{{\rm{T}}}}}^{{\prime} }$-MoTe_2_, we analyze how bulk-to-boundary proximity effects can generate an effective *s* + *p*-wave pairing structure on the higher-order boundary. The helical Dirac surface states of the SOTI band provide a natural venue for generating unconventional pairing. In particular, it is well established that *p*-wave correlations can emerge when an *s*-wave superconductor is proximitized to the surface states of a topological insulator^37,38^. In the present context, the coexistence of a bulk *s*-wave SC band and helical SOTI surface states in $1{{{{\rm{T}}}}}^{{\prime} }$-MoTe_2_ realizes this mechanism intrinsically; single-particle tunneling between the bulk band and the SOTI band gives rise to a bulk-to-surface self-proximity effect that induces a *p*-wave component in the surface pairing. To analyze this mechanism and the higher-order nature of the SC $1{{{{\rm{T}}}}}^{{\prime} }$ phase, we construct a BdG Hamiltonian by coupling the bulk *s*-wave band to the SOTI band via a tunneling term [Sec. S3.2.1].

To explicitly determine the induced SC pairing generated by this bulk-to-surface proximity mechanism, we first derive the effective SC Hamiltonian for the SOTI band by integrating out the bulk *s*-wave band using the Schrieffer-Wolff transformation^57^ [Sec. S3.2.2]. This procedure generates two *s*-wave and three *p*-wave pairing channels in the resulting effective BdG description; the corresponding fermion bilinear forms are summarized in Table 1. The surface pairing structures are then obtained from a low-energy surface BdG Hamiltonian, constructed by projecting the bulk effective BdG Hamiltonian onto a cylindrical surface^58^. On the surface, two *s*-wave pairings (Δ_*s*1_, Δ_*s*2_) and one *p*-wave pairing (Δ_*p*3_) survive, whereas the other two *p*-wave channels vanish under the projection [Sec. S3.2.3]. Therefore, we theoretically demonstrate that both *s*- and *p*-wave surface superconductivity are induced by the bulk *s*-wave superconductivity [Fig. 1e].

**Table 1:** Induced *s*- and *p*-wave pairings in the bulk system

*s*-wave	Fermion Bilinear	Matrix Form
Δ_*s*1_	$i{\sum }_{j=1}^{2}\left[-{c}_{j,\uparrow }^{{{\dagger}} }{c}_{j+2,\downarrow }^{{{\dagger}} }+{c}_{j,\downarrow }^{{{\dagger}} }{c}_{j+2,\uparrow }^{{{\dagger}} }\right]+ i{\sum }_{k=1}^{2}{(-1)}^{k-1}\left[{c}_{k,\uparrow }^{{{\dagger}} }{c}_{5-k,\downarrow }^{{{\dagger}} }-{c}_{k,\downarrow }^{{{\dagger}} }{c}_{5-k,\uparrow }^{{{\dagger}} }\right]$+h.c.	${\xi }_{x}\left({\tau }_{x}+{\tau }_{y}{\mu }_{y}\right){\sigma }_{y}$
Δ_*s*2_	$i{\sum }_{j=1}^{2}{(-1)}^{j}\left({c}_{j,\uparrow }^{{{\dagger}} }{c}_{j+2,\downarrow }^{{{\dagger}} }-{c}_{j,\downarrow }^{{{\dagger}} }{c}_{j+2,\uparrow }^{{{\dagger}} }\right)$+h.c.	*ξ*_*x*_*τ*_*x*_*μ*_*x*_*σ*_*y*_

*p*-wave	Fermion Bilinear	Matrix Form
Δ_*p*1_	$i\left(\sin {k}_{x}-\sin {k}_{z}\right){\sum }_{j}{(-1)}^{j-1}\left({c}_{j,\uparrow }^{{{\dagger}} }{c}_{j+2,\downarrow }^{{{\dagger}} }-{c}_{j,\downarrow }^{{{\dagger}} }{c}_{j+2,\uparrow }^{{{\dagger}} }\right)$+h.c.	$\left(\sin {k}_{x}-\sin {k}_{z}\right){\xi }_{y}{\mu }_{y}{\sigma }_{y}$
Δ_*p*2_	$\sin {k}_{y}{\sum }_{j}\left({c}_{j,\uparrow }^{{{\dagger}} }{c}_{j,\downarrow }^{{{\dagger}} }+{c}_{j,\downarrow }^{{{\dagger}} }{c}_{j,\uparrow }^{{{\dagger}} }\right)$+h.c.	$\sin {k}_{y}{\xi }_{x}{\sigma }_{x}$
Δ_*p*3_	$\sin {k}_{y}{\sum }_{j}{(-1)}^{{\left[\frac{j-1}{2}\right]}_{{{{\rm{floor}}}}}}\left({c}_{j,\uparrow }^{{{\dagger}} }{c}_{j,\downarrow }^{{{\dagger}} }+{c}_{j,\downarrow }^{{{\dagger}} }{c}_{j,\uparrow }^{{{\dagger}} } \right)+ \sin {k}_{y}{\sum }_{k=1}^{2}\left({c}_{2k-1,\uparrow }^{{{\dagger}} }{c}_{2k,\downarrow }^{{{\dagger}} }+{c}_{2k-1,\downarrow }^{{{\dagger}} }{c}_{2k,\uparrow }^{{{\dagger}} }\right)$+h.c.	$\sin {k}_{y}{\xi }_{x}\left({\tau }_{z}+{\mu }_{x}\right){\sigma }_{x}$

The pairings are expressed as fermion bilinears and in their respective matrix forms. In the column of fermion bilinear, the index *j* indicates the orbital, with the allowed indices *j* = 1, 2, 3, 4. The 16 × 16 matrix form for the pairing functions is expressed by the tensor product of Pauli matrices Δ(**k**)*ξ*_*i*_*τ*_*j*_*μ*_*k*_*σ*_*l,*_ except for the coefficient for the SC gap function Δ_*i*_. The Pauli matrices *ξ*_*i*_ indicate the Nambu space, *τ*_*j*_ and *μ*_*k*_ indicate orbitals, and *σ*_*l*_ indicate the spin degrees of freedom.

The calculated temperature and magnetic-field dependence of the induced *s*- and *p*-wave gaps capture the key features of the experimental PCAR spectra [Fig. 4c, f]; both components share the same critical temperature, and the *p*-wave contribution is fragile, being readily suppressed by breaking inversion symmetry or by an applied magnetic field. This consistency supports our interpretation that the observed surface *s* + *p*-wave response in the $1{{{{\rm{T}}}}}^{{\prime} }$ phase originates from a bulk-to-surface self-proximity effect in a higher-order topological band structure, providing the microscopic basis for the emergence of Majorana corner states in the TOTSC phase discussed below.

Taken together with the SOTI boundary structure, the induced *s*- and *p*-wave components provide a minimal route toward a TOTSC phase under suitable conditions. To make this connection explicit, we track how the helical hinge modes of the normal SOTI band evolve under SC pairing using complementary numerical and analytical approaches, and identify the resulting gapped hinge phase as a one-dimensional topological superconductor that can support Majorana corner states at its terminations.

In the normal SOTI phase, gapless helical hinge states appear along the geometrical hinges [Fig. 5a, b]. When superconductivity is introduced, these hinge modes acquire a gap and evolve into massive hinge states (green lines) in the TOTSC phase [Fig. 5c]. Figure 5c displays the band structure of a finite system with a square cross-section in the *xz* plane and periodic boundary conditions along the *y* axis (the *C*_2_ symmetry axis). The dispersion is obtained both numerically, from a tight-binding (TB) BdG Hamiltonian, and analytically, using a Jackiw–Rebbi domain-wall construction^59^. The excellent agreement between the analytic hinge dispersion of Eq. (10) (green) and the numerical bands (black) confirms that the SOTI hinge channels are fully gapped in the SC state, as shown in Fig. 5c, d [see Sec. S3.2.5 for the analytical dispersion]. This gapped hinge system effectively realizes a one-dimensional topological superconductor along each hinge, which can support Majorana corner states at its terminations.

To directly identify these corner states, we compute the energy spectrum of a fully finite cubic sample using the TB BdG Hamiltonian. We find four nearly zero-energy in-gap states [Fig. 5c] whose energies approach zero exponentially with increasing system size; the residual splitting is a finite-size effect of the numerical calculation, whereas the theoretical treatment yields exact zero modes in the thermodynamic limit. The theoretical analysis for the zero-energy mode is given for the simplified case in Sec. S3.2.5 and for the more general case in Sec. S3.2.7. The corresponding real-space probability densities demonstrate that each mode is exponentially localized at one of the four corners of the cubic geometry, establishing their corner character [Fig. 5d]. The same physics can be captured in a spherical geometry [Fig. 5e]; the zero modes are concentrated near the north and south poles (*θ* = 0, *π* with respect to the *y* axis), and can be viewed as four localized wavefunctions sitting at the poles and then smoothly descending and splitting into the four corners when the sphere is deformed into a cube.

The resulting zero-energy Majorana corner states arising in cylindrical and spherical geometry form Majorana Kramers’ pairs^60,61^. Denoting the four corners (or poles) by *j* = *T*, *B*, *N*, *S* (top, bottom, north, south), we label the corresponding wavefunctions by ${\psi }_{j}^{\pm }$. They are localized at the top and bottom (north and south) poles, ${\psi }_{T,B}^{\pm }$ (${\psi }_{N,S}^{\pm }$), and are constrained by particle-hole and time-reversal symmetries; $C{\psi }_{T,B,N,S}^{\pm }=\pm {\psi }_{T,B,N,S}^{\pm }$ and $\Theta {\psi }_{T,B,N,S}^{\pm }=\mp i{\psi }_{T,B,N,S}^{\mp }$ with the charge-conjugation and time-reversal operators *C* = *ξ*_*x*_*K*, *Θ* = *iσ*_*y*_*K*, respectively, where *ξ*_*x*_ acts in Nambu space, *σ*_*y*_ acts in spin space, and *K* denotes complex conjugation. The relations ${({\psi }_{T,B,N,S}^{\pm })}^{C}=\pm {\psi }_{T,B,N,S}^{\pm }$ demonstrate that each state is self-conjugate under particle-hole symmetry and, hence, is a Majorana mode. Because time reversal interchanges ${\psi }_{j}^{+}$ and ${\psi }_{j}^{-}$ at each corner, these modes come in Kramers’ pairs protected by *Θ*. Consequently, no linear combination of these Majorana pairs can be deformed into a purely electron-like or hole-like state without breaking either particle-hole or time-reversal symmetry. Further details of the construction and symmetry properties of the Majorana corner modes are provided in Methods [see also Secs. S3.2.6, S3.2.8].

Within the Altland–Zirnbauer classification, the BdG Hamiltonian of the SC $1{{{{\rm{T}}}}}^{{\prime} }$ phase, endowed with both time-reversal and particle-hole symmetries, belongs to class DIII and is therefore allowed to host topological Majorana boundary states^32,62,63^. The bulk topology is captured by a ${{\mathbb{Z}}}_{8}$ index^32,64,65^,

1 $$\kappa=\frac{1}{4}{\sum}_{K\in {{{\rm{TRIM}}}}}\left[{N}^{+}(K)-{N}^{-}(K)\right]\,{{{\rm{mod}}}}\,8,$$

where *N*^±^(*K*) indicates the number of occupied states with inversion eigenvalues  ± 1 at each time-reversal-invariant momentum (TRIM) *K*. In this convention, *κ* = 0 corresponds to a trivial phase, *κ* = 1, 3, 5, 7 to first-order topological superconductors with gapless surface states, *κ* = 2, 6 to second-order phases with hinge Majorana modes, and *κ* = 4 to third-order phases hosting Majorana corner states. Parity eigenvalues for each TRIM point are given in Fig. 5f, and the resulting value *κ* = 4 identifies the TOTSC phase. Crucially, the full Hamiltonian *H*_Tot_ of Eq. (7), which describes the SOTI bands coupled to a bulk *s*-wave superconductor, and the effective proximity-induced SC SOTI Hamiltonian of Eq. (8), obtained via the Schrieffer-Wolff transformation, share the same bulk invariant, *κ* = 4, as explicitly verified in Fig. S11 and detailed in Sec. S3.2.4.

This analysis shows that the induced *p*-wave SC gap is governed by helical Dirac surface states that are stabilized by the combined action of inversion and time-reversal symmetries in the $1{{{{\rm{T}}}}}^{{\prime} }$ phase. Under high pressure, restoration of inversion symmetry reinforces these helical surface states, which enhances bulk-to-surface proximity coupling and thereby promotes the formation of a robust *p*-wave component. In contrast, upon lowering the pressure into the T_*d*_ phase, the helical Dirac surface states disappear as inversion symmetry is broken, suppressing the *p*-wave gap and, consequently, topological superconductivity. Taken together with the experimental observation of a proximity-induced *s* + *p*-wave surface gap, these results demonstrate that the topological SC behavior of MoTe_2_ is symmetry-controlled and tunable via inversion-symmetry restoration under pressure, providing a route to realizing Majorana corner states in a bulk crystal.

Note that our present experiments do not directly image hinge or corner modes. Future experiments such as angle-resolved PCAR, hinge/corner selective tunneling spectroscopy, and thermal transport anisotropy along hinges/corners will be needed to establish unambiguous evidence of the TOTSC phase and Majorana corner states^40,66–71^.

## Discussion

In conclusion, we have systematically investigated the interplay between topology and superconductivity in MoTe_2_ across the noncentrosymmetric T_*d*_ and centrosymmetric $1{{{{\rm{T}}}}}^{{\prime} }$ phases. Using surface-sensitive soft point-contact Andreev reflection spectroscopy under variable temperature, magnetic field, and extreme pressure, together with quantitative theoretical modeling, we map how the SC response evolves across the pressure-driven structural and topological transformation. In the pressure-tuned T_*d*_ phase, the spectra reveal a two-gap *s*-wave SC state associated with the multiband type-II Weyl semimetal, consistent with previous multi-gap observations at ambient pressure^26^. Upon increasing pressure, MoTe_2_ transforms into the $1{{{{\rm{T}}}}}^{{\prime} }$ phase, where a pronounced single peak near zero-bias between double conductance maxima is captured by an extended BTK analysis with coexisting *s*- and *p*-wave components. Our analyses imply that the *p*-wave gap is linked to spin-momentum-locked helical Dirac surface states of an SOTI band, stabilized by inversion and time-reversal symmetries in $1{{{{\rm{T}}}}}^{{\prime} }$. Restoration of inversion symmetry under pressure reinforces these surface states, inducing bulk-to-surface proximity coupling and stabilizing the *p*-wave component, whereas in the T_*d*_ phase, broken inversion symmetry removes this protection, suppressing the *p*-wave gap and, consequently, topological superconductivity. Our theoretical analysis further supports a scenario in which the higher-order boundary modes evolve into a gapped hinge phase and can host zero-energy Majorana corner modes in an effective TOTSC regime. These findings indicate that MoTe_2_ is a symmetry-controlled, pressure-tunable platform for exploring topological superconductivity, providing a pathway toward engineering Majorana modes in quantum materials.

## Methods

### Experimental methods

Under quasi-hydrostatic pressure conditions, MoTe_2_ undergoes a phase transition from the T_*d*_ to $1{{{{\rm{T}}}}}^{{\prime} }$ phases. To create quasi-hydrostatic pressure environments, the samples are enclosed within a Teflon tube and pressurized using a clamp-type hybrid cell filled with Daphne 7373 as the pressure-transmitting medium. This setup allows for the application of quasi-hydrostatic pressures up to 2.7 GPa. The pressure inside the cell at low temperatures is determined by monitoring the sharp resistivity drop at the SC transition temperature of Pb and Sn^72,73^.

Soft point-contact spectroscopy is a highly sensitive technique for probing the SC gap structure and surface properties of materials^24^. In this study, we employed soft point-contact spectroscopy to investigate the SC gap structure of MoTe_2_ across the pressure-driven transition from the T_*d*_ to the $1{{{{\rm{T}}}}}^{{\prime} }$ phase. Point contacts were formed by depositing fine silver grains onto the sample surface under quasi-hydrostatic pressure. After screening numerous crystals and contact configurations, we identified conditions that yield stable soft point-contact spectra under pressure. For the measurements reported here, the contacts remained in the ballistic regime under high pressure without crossing over into the thermal regime. The temperature was controlled down to 0.25 K using a ^3^He Heliox system (Oxford Instruments Nanotechnology).

The PCAR spectra and upper critical field data were analyzed using a combination of BTK, WHH, and multi-gap BCS models, as detailed in the Supplementary Information [Sec. S1]. The fitting methodology is given in Sec. S1.1, and fitting parameters are summarized in Tables S1–S7.

For the T_*d*_ phase, temperature- and field-dependent PCAR spectra at 0.7 GPa were analyzed using both single-gap and extended two-gap *s*-wave BTK models, with the resulting gap amplitudes further constrained by self-consistent solutions of the coupled two-band BCS gap equations [Sec. S1.2]. To assess the robustness of this multi-gap behavior, we additionally performed complementary PCAR measurements at 1.2 GPa, applying the same temperature- and field-dependent BTK framework and comparing the evolution of the two gaps between the two pressure points [Secs. S1.2.4, S1.2.5]. The corresponding upper critical field *H*_*c*2_(*T*) was extracted from both pressure datasets and analyzed within single- and two-band WHH models, with only the multiband description reproducing the pronounced downward curvature near *T*_*c*_ [Sec. S1.2.3].

For the pressure-induced $1{{{{\rm{T}}}}}^{{\prime} }$ phase, we carried out a systematic BTK comparison of anisotropic *s*-wave, ${d}_{{x}^{2}-{y}^{2}}$-wave, and mixed *s* + *p*-wave order parameters, and we quantified the quality of each description by analyzing the bias-resolved residuals and the integrated squared deviation between the experimental and calculated conductance [Sec. S1.3]. The systematic exclusion of thermal, multi-gap *s*-wave, and impurity-induced origins of the single peak near zero-bias is discussed in detail in Sec. S1.4.

### Theoretical methods

We interpret the pressure-dependent SC phases of MoTe_2_ by combining TB descriptions of the normal-state band structures with effective BdG models and multi-gap BCS gap equations. Starting from a PT-symmetric Dirac semimetal in the high-symmetry T_0_ phase, we introduce inversion-breaking and shear distortions to construct low-energy models for the orthorhombic T_*d*_ phase and the monoclinic $1{{{{\rm{T}}}}}^{{\prime} }$ phase, respectively. In the T_*d*_ phase, the Dirac cone splits into type-II Weyl cones, and superconductivity is modeled by a two-gap *s*-wave BdG Hamiltonian describing a parabolic band hybridized with a tilted Weyl band [Sec. S2.1]. From these BdG models, we derive coupled multi-gap BCS equations to analyze the temperature and magnetic-field dependence of the SC gaps in the T_*d*_ phase [Sec. S2.2]. In the $1{{{{\rm{T}}}}}^{{\prime} }$ phase, the normal state is described by a SOTI Hamiltonian [Sec. S3.1], and superconductivity is introduced by coupling a bulk *s*-wave BCS band to the SOTI bands [Sec. S3.2.1], which generates effective *s* + *p*-wave pairing on the higher-order topological surface. A Schrieffer-Wolff transformation is performed to obtain the induced *s*- and *p*-wave pairings in the $1{{{{\rm{T}}}}}^{{\prime} }$ phase [Sec. S3.2.2]. Projecting the induced BdG Hamiltonian of the $1{{{{\rm{T}}}}}^{{\prime} }$ phase onto the surfaces and hinges, and solving the resulting Dirac equations by Jackiw-Rebbi methods, we derive effective *s* + *p*-wave hinge Hamiltonians and demonstrate the existence of Majorana Kramers’ pairs localized at the corners [Secs. S3.2.3, S3.2.6 and S3.2.8]. The bulk topology of the SC $1{{{{\rm{T}}}}}^{{\prime} }$ phase is characterized by the ${{\mathbb{Z}}}_{8}$ higher-order topological index *κ* defined in Eq. (1), evaluated from inversion parities at the TRIM, which implies the TOTSC phase [Sec. S3.2.4]. The TB parameters used in Fig. 5 are *t*_*x*_ = *t*_*z*_ = 1, *t*_*y*_ = 2, *λ*_*x*_ = *λ*_*z*_ = 1, *λ*_*y*_ = 0.5, *m*_0_ = − 3, *m*_1_ = *m*_2_ = 0.3, Δ_*s*1_ = Δ_*p*1_ = 0, Δ_*s*2_ = 0.1, Δ_*p*2_ = 0.05, Δ_*p*3_ = 0.04, and *μ* = 0.05. Setting Δ_*s*1_ = Δ_*p*1_ = 0 simplifies the surface Hamiltonian but does not change the ${{\mathbb{Z}}}_{8}$ index defined in Eq. (1), so that the simplified model remains in the TOTSC phase.

In the SC T_*d*_ phase, we construct a minimal model that captures the multi-gap system observed in the PCAR spectra. For the normal state, we start from a PT-symmetric Dirac Hamiltonian that illustrates the T_0_ phase, in which a Dirac cone is realized near the *Γ* point, and then introduce inversion-breaking mass terms and a momentum-dependent tilt to obtain a type-II Weyl Hamiltonian for the T_*d*_ phase. To model the low-energy SC properties, we retain only the Weyl cone closest to the Fermi level and supplement it with an additional parabolic band centered at *Γ*, which is motivated by the normal-state band structure and serves as a natural second SC gap [Sec. S2.1].

We then introduce an *s*-wave pairing interaction on both bands and write a two-gap BdG Hamiltonian in terms of electron operators *a*_*k*,*s*_ for the parabolic band and *c*_*k*,*s*_ for the Weyl band. The mean-field interaction contains intraband pairing amplitudes *U* and *V* in the parabolic and Weyl bands, respectively, together with an interband coupling *W* that encodes proximity-type hybridization between the two condensates. Defining SC order parameters Δ_1_ and Δ_2_ on the parabolic and Weyl bands, the quasi particle dispersions are $E_{k,i,\pm} = \lambda_{i,k}\pm\sqrt{\xi_{k,i}^2+|\Delta_i|^2}$, where $\lambda_{i,k} \; {{\rm{and}}} \; \xi_{k,i}$ are the momentum odd and even parts of the band energies measured from the chemical potential. Minimizing the mean-field free energy leads to coupled two-gap BCS equations of the form

2 $${\Delta}_{1}=U {\sum}_{k,s} \frac{{\Delta}_{1}}{4s {E}_{k,1}} {{\tanh}} \frac{{\beta} {E}_{k,1,s}}{2}+W {\sum}_{k,s} \frac{{\Delta}_{2}}{4s {E}_{k,2}}{{\tanh}} \frac{{\beta} {E}_{k,2,s}}{2},$$

3 $${\Delta}_{2}=V {\sum}_{k,s} \frac{{\Delta}_{2}}{4s {E}_{k,2}}{{\tanh}} \frac{{\beta} {E}_{k,2,s}}{2}+W {\sum}_{k,s}\frac{{\Delta}_{1}}{4s {E}_{k, 1}}{{\tanh}} \frac{{\beta} {E}_{k,1,s}}{2} . $$

These equations are solved numerically to obtain the temperature dependence of the two gaps, Δ_1_(*T*) and Δ_2_(*T*), which are directly compared with the gap amplitudes extracted from the two-gap BTK fits to the PCAR spectra in the T_*d*_ phase [Sec. S2.2].

The interband coupling *W* is found to be essential for reproducing the long tail of the smaller gap toward *T*_*c*_, as well as its rapid suppression compared with the larger gap. In the limit where *W* becomes 0, the smaller gap collapses at a much lower temperature than observed experimentally, which is incompatible with the PCAR data. We also account for the magnetic-field dependence of the gaps by including a Zeeman term in the quasiparticle energies, $\bar{\lambda}_{k,i}= \lambda_{k,i}- B$, and tracking the evolution of Δ_1_ and Δ_2_ as a function of field at fixed low temperature. Altogether, this minimal Weyl-plus-parabolic-band model yields a self-consistent description of the multi-gap superconductivity in T_*d*_-MoTe_2_ under pressure.

In the SC $1{{{{\rm{T}}}}}^{{\prime} }$ phase, we exploit the higher-order band topology of MoTe_2_ and show that a normal-state SOTI band can evolve into the TOTSC phase via a bulk-to-surface self-proximity effect. The normal-state bands in the monoclinic $1{{{{\rm{T}}}}}^{{\prime} }$ structure are modeled by a lattice SOTI Hamiltonian that encodes the effect of the shear distortion relative to the T_0_ phase through spin-orbit coupling and symmetry-allowed mass terms [Sec. S3.1.1]. The TB Hamiltonian for the SOTI bands takes the form

4 $${H}_{{{{\rm{SOTI}}}}}({{{\bf{k}}}})=	 \left({m}_{0}+{\sum}_{i=x,y,z}{t}_{i}\cos {k}_{i}\right){\tau }_{z}+{\lambda }_{x}\sin {k}_{x}{\tau }_{y}{\mu }_{y}+{\lambda }_{z}\sin {k}_{z}{\tau }_{x} \\ 	+{\lambda }_{y}\sin {k}_{y}{\tau }_{y}{\mu }_{z}{\sigma }_{z}+{m}_{1}{\tau }_{z}{\mu }_{z}+{m}_{2}{\mu }_{x},$$

where the Pauli matrices *τ*_*i*_ and *μ*_*j*_ act on two orbital subspaces and *σ*_*k*_ on spin. The parameters *t*_*i*_ describe nearest-neighbor hopping, the coefficients *λ*_*j*_ implement spin-orbit or orbital-dependent hoppings, and *m*_1_ and *m*_2_ are symmetry-allowed mass terms that drive a double band inversion and gap out the surfaces while preserving gapless hinge modes. This Hamiltonian respects inversion and time-reversal symmetries appropriate for the $1{{{{\rm{T}}}}}^{{\prime} }$ structure and realizes helical hinge channels in a finite geometry [Sec. S3.1.2].

Superconductivity in the $1{{{{\rm{T}}}}}^{{\prime} }$ phase is introduced by coupling these SOTI bands to a low-energy bulk *s*-wave SC band centered at the *Γ* point. The bulk *s*-wave band is modeled by a simple BdG Hamiltonian

5 $${H}_{{{{\rm{SC}}}}}({{{\bf{k}}}})=\left(-{k}_{y}^{2}+{\varepsilon }_{0}\right){\xi }_{z}+{\Delta }_{s-{{{\rm{wave}}}}}{\xi }_{x}{\tau }_{z}{\mu }_{z}{\sigma }_{y},$$

where *ξ*_*i*_ are Nambu space Pauli matrices, and *ε*_0_ is an energy shift. Coupling between the SOTI and SC bands is

6 $${H}_{{{{\rm{I}}}}}={\sum}_{j,s}\lambda \left({a}_{j,s}^{{{\dagger}} }{c}_{j,s}+{c}_{j,s}^{{{\dagger}} }{a}_{j,s}\right),$$

where ${a}_{j,s}^{{{\dagger}} }$ and ${c}_{j,s}^{{{\dagger}} }$ create electrons with spin *s* in the bulk *s*-wave and SOTI bands, respectively, and *λ* is the tunneling amplitude. The full BdG Hamiltonian of the coupled system is then

7 $${H}_{{{{\rm{Tot}}}}}({{{\bf{k}}}})=\left(\begin{array}{cc}{H}_{{{{\rm{SC}}}}}({{{\bf{k}}}}) & {H}_{{{{\rm{I}}}}}\\ {H}_{{{{\rm{I}}}}}^{{{\dagger}} } & {H}_{{{{\rm{SOTI}}}}}^{{{{\rm{BdG}}}}}({{{\bf{k}}}})\end{array}\right),$$

where ${H}_{{{{\rm{SOTI}}}}}^{{{{\rm{BdG}}}}}({{{\bf{k}}}})$ is the BdG expansion of the SOTI Hamiltonian in Eq. (4) [Sec. S3.2.1].

Treating *H*_I_ perturbatively, we perform a Schrieffer-Wolff transformation to integrate out the bulk *s*-wave band and obtain an effective induced BdG Hamiltonian acting on the SOTI sector alone. To leading order in *λ,* this procedure generates a hierarchy of induced pairing channels, consisting of two *s*-wave terms and three *p*-wave terms on the SOTI bands, which are analytic functions of the microscopic parameters Δ_*s*−wave_, *λ*, *λ*_*y*_, *m*, and *μ* [Sec. S3.2.2]. A key result is that the proximity effect does not simply induce a trivial on-site *s*-wave pairing but a mixture of *s*- and *p*-wave pairings on the surface, as expected for helical surface states proximitized by a conventional superconductor [Sec. S3.2.3].

The resulting induced BdG Hamiltonian for the SOTI bands can be written schematically as

8 $${H}^{{{{\rm{BdG}}}}}({{{\bf{k}}}})=\left(\begin{array}{cc}{H}_{{{{\rm{SOTI}}}}}({{{\bf{k}}}})-\mu & \Delta ({{{\bf{k}}}})\\ {\Delta }^{{{\dagger}} }({{{\bf{k}}}}) & -{H}_{{{{\rm{SOTI}}}}}^{T}(-{{{\bf{k}}}})+\mu \end{array}\right),$$

where Δ(**k**) collects the induced *s*- and *p*-wave pairing terms listed in Table 1. Because the normal state is monoclinic and the SC transition does not change the crystal structure, both *H*_SOTI_(**k**) and *H*^BdG^(**k**) preserve inversion and time-reversal symmetries, and thus the BdG system remains PT symmetric. Evaluating the ${{\mathbb{Z}}}_{8}$ index *κ* shows that *κ* = 4, confirming that the induced SC phase is a TOTSC. We focus on the dominant induced pairings and set Δ_*s*1_ = Δ_*p*1_ = 0 while retaining Δ_*s*2_, Δ_*p*2_, and Δ_*p*3_, which leaves the bulk ${{\mathbb{Z}}}_{8}$ index unchanged [Sec. S3.2.4]. The results of a non-zero Δ_*s*1_ and Δ_*p*1_ are given in Sec. S3.2.7.

To connect this bulk description to the boundary phenomenology relevant for PCAR, we project *H*^BdG^(**k**) onto surface-localized states obtained from an effective Dirac equation in cylindrical geometry with open boundaries in the *xz* plane and periodic boundary conditions along *y*. The projected surface BdG Hamiltonian is defined by

9 $${\left[{H}_{{{{\rm{Surf}}}}}^{{{{\rm{BdG}}}}}({{{\bf{k}}}})\right]}_{ij}=\left\langle {\psi }_{i}\right|{H}^{{{{\rm{BdG}}}}}({{{\bf{k}}}})\left|{\psi }_{j}\right\rangle,$$

which yields a surface BdG Hamiltonian in which two *s*-wave components and a single *p*-wave component survive. At the hinge angle $\phi=\frac{\pi }{4}$, where the surface Dirac mass changes sign, the surface BdG Hamiltonian reduces to an effective one-dimensional hinge Hamiltonian of the form

10 $${H}_{{{{\rm{Hinge}}}}}^{{{{\rm{BdG}}}}}({k}_{y})=-\mu {\xi }_{z}+{\lambda }_{y}\sin {k}_{y}{\sigma }_{z}+{\Delta }_{s}{\xi }_{y}{\sigma }_{y}+{\Delta }_{p}\sin {k}_{y}{\xi }_{x}{\sigma }_{x},$$

where Δ_*s*_ ≡ Δ_*s*2_ and ${\Delta }_{p}\equiv \frac{\sqrt{2}+1}{\sqrt{2}}{\Delta }_{p3}$ for notational simplicity. The corresponding hinge dispersion is fully gapped and controlled by the *s*- and *p*-wave pairings, illustrating the effective *s* + *p*-wave of the surface [Sec. S3.2.5].

Replacing *k*_*y*_ with −*i*∂_*y*_ in Eq. (10) and solving the associated Dirac equation by the Jackiw-Rebbi method on a finite hinge segment, $-\frac{L}{2} < y < \frac{L}{2}$, we obtain zero-energy bound states localized at the ends of the hinge. These solutions form Kramers’ pairs of Majorana modes at the top and bottom corners, which satisfy particle-hole symmetry and are related by time-reversal symmetry [Sec. S3.2.6]. An analogous construction in spherical geometry leads to Majorana Kramers’ pairs localized at the north and south poles, providing a complementary three-dimensional view of the same TOTSC phase [Sec. S3.2.8].

## Supplementary information

Supplementary Information

Transparent Peer Review file

## Data Availability

All data supporting this paper are available within the article and the Supplementary Information. Additional data related to this study are available from the corresponding authors upon request.
