# Supplementary material for: Symmetry-controlled multi-gap superconductivity and higher-order topological phases of MoTe2
Source: Nat Commun. 2026 May 19;17:7685. doi: 10.1038/s41467-026-72368-x (PMC13434778; doi:10.1038/s41467-026-72368-x)
Supplement: Supplementary file 1 [file 41467_2026_72368_MOESM1_ESM.pdf]

Supplementary Information: Symmetry-Controlled Multi-gap Superconductivity and Higher-Order Topological Phases of $MoTe_2$

Sangyun Lee,[1,2,3,*] Myungjun Kang,[4,5,6,*] Jihyun Kim,[1] Suyeon Cho,[7] Duk Y. Kim,[8] Sangmo Cheon,[4,5,9,10,†] and Tuson Park[1,‡]

[1]*Center for Quantum Materials and Superconductivity (CQMS) and Department of Physics, Sungkyunkwan University, Suwon 16419, South Korea*

[2]*Los Alamos National Laboratory, Los Alamos, NM 87545, USA*

[3]*Department of Physics and National High Magnetic Field Laboratory, University of Florida, Florida 32611, USA*

[4]*Department of Physics, Hanyang University, Seoul 04763, South Korea*

[5]*High Pressure Research Center, Hanyang University, Seoul 04763, South Korea*

[6]*Department of Physics, University of Wisconsin–Milwaukee, Milwaukee, Wisconsin 53201, USA*

[7]*Division of Chemical Engineering and Materials Science, Ewha Womans University, Seoul, 03760, South Korea*

[8]*Agency for Defense Development, Daejeon 34186, South Korea*

[9]*Research Institute for Natural Science, Hanyang University, Seoul 04763, South Korea*

[10]*Hanyang Institute for Quantum Science and Quantum Technology, Hanyang University, Seoul 04763, South Korea*

---

[*] These authors contributed equally: Sangyun Lee and Myungjun Kang

[†] Corresponding authors:sangmocheon@hanyang.ac.kr

[‡] Corresponding authors:tp8701@skku.edu

CONTENTS

S1. Experimental Details 3

S1.1. Fitting Methodology 3

S1.1.1. Blonder-Tinkham-Klapwijk (BTK) Formalism for PCAR Spectra 3

S1.1.2. BTK Models for Anisotropic s-wave, p-wave, and $d_{x^2-y^2}$-wave Pairings 5

S1.1.3. Extended BTK Model for Multi-Gap Pairing 6

S1.2. Experimental Data and Analysis of Multi-Gap Superconductivity in the T$_d$ Phase 7

S1.2.1. Comparison between Single and Two-Gap BTK Fits to PCAR Data at 0.7 GPa 7

S1.2.2. Temperature- and Field-Dependent BTK Fitting Parameters at 0.7 GPa 9

S1.2.3. Werthamer-Helfand-Hohenberg (WHH) Analysis of the Upper Critical Field 11

S1.2.4. Alternative PCAR Evidence for Two-Gap Superconductivity at 1.2 GPa 13

S1.2.5. Temperature- and Field-Dependent BTK Fitting Parameters at 1.2 GPa 15

S1.2.6. Revealing Multi-Gap Pairing via Magnetic-Field-Dependent PCAR 17

S1.3. BTK Analysis for Superconducting Pairing Symmetries in the 1T′ Phase 19

S1.3.1. Quantitative Comparison of BTK Fits for Different Pairing Symmetries 19

S1.3.2. BTK comparison of s- and p-wave pairing in the 1T′ phase 21

S1.3.3. BTK Simulations of Competing Gap Symmetries in the 1T′ Phase 23

S1.3.4. BTK Parameterization of s+p-Wave Superconductivity at 2.2 GPa 25

S1.4. Systematic Exclusion of Extrinsic Mechanisms for the Single Peak near Zero-Bias 27

S1.4.1. Broad Zero-Bias Conductance Hump in the Thermal Regime 27

S1.4.2. Excluding a Two-Gap s-wave Origin of the Single Peak near Zero-Bias 29

S1.4.3. Ruling out impurity-induced origins of the Single Peak near Zero-Bias 31

S2. Theoretical Analysis for Superconducting T$_d$ Phase 33

S2.1. Minimal tight-binding model for the T$_d$ phase 33

S2.1.1. Inversion-symmetry breaking and Weyl nodes in the T$_d$ phase 34

S2.2. Coupled two-gap equation in the T$_d$ phase 36

S3. Theoretical Analysis for Superconducting 1T′ Phase 38

S3.1. Second-Order Topological Insulator: Normal State Model 38

S3.1.1. Lattice SOTI Hamiltonian and Symmetries 38

S3.1.2. Analytical Derivation of Topological Hinge States 40

S3.1.3. Effective Surface Hamiltonian: Two Copies of a Helical Dirac Cone 41

S3.2. Proximity-Induced Surface s+p-wave Topological Superconductor 42
S3.2.1. Model System: Bulk s-wave Band Coupled to SOTI Bands 42
S3.2.2. Schrieffer-Wolff Transformation and Induced Bulk Pairings 43
S3.2.3. Effective Induced BdG Hamiltonian and Surface s+p-wave Pairing 45
S3.2.4. Topological $\mathbb{Z}_8$ Index and Robustness of the TOTSC Phase 46
S3.2.5. Hinge Hamiltonian and Majorana Physics in Cylindrical Geometry 48
S3.2.6. Majorana Corner States in Cylindrical Geometry 49
S3.2.7. Effect of Additional Pairing Terms in Cylindrical Geometry 50
S3.2.8. Majorana Corner States in Spherical Geometry 51

References 54

S1. EXPERIMENTAL DETAILS

S1.1. Fitting Methodology

S1.1.1. Blonder-Tinkham-Klapwijk (BTK) Formalism for PCAR Spectra

In this section, we summarize the Blonder-Tinkham-Klapwijk (BTK) formalism used to fit the point-contact Andreev reflection (PCAR) spectra measured in both the T_d and $1T'$ phases of $MoTe_2$ [1, 2]. The BTK model describes quasiparticle transport across a normal metal-superconductor interface by treating on equal footing the processes of normal reflection, Andreev reflection, and transmission through a finite interfacial barrier.

Within this framework, the normalized differential conductance $G(V)$ at finite temperature can be written as

$$G(V) = \int_{-\infty}^{\infty} [1 + A(E) - B(E)] \left(-\frac{\partial f(E - eV)}{\partial E} \right) dE, \tag{S1}$$

where $A(E)$ and $B(E)$ are the energy-dependent Andreev- and normal-reflection probabilities, respectively, and $f(E)$ is the Fermi-Dirac distribution function. At zero temperature ($T = 0$), the derivative of the Fermi function reduces to a Dirac delta function, so that the conductance simplifies to

$$G(V, T = 0) = 1 + A(eV) - B(eV). \tag{S2}$$

A convenient and widely used formulation expresses the spectral conductance in terms of the interface transparency τ_N and the quasiparticle coherence function $\gamma(E)$ as

$$\sigma(E) = \tau_N \frac{1 + \tau_N |\gamma(E)|^2 + (\tau_N - 1)|\gamma(E)^2|^2}{|1 + (\tau_N - 1)\gamma(E)^2|^2}, \tag{S3}$$

where

$$\gamma(E) = \frac{E + i\Gamma - \sqrt{(E + i\Gamma)^2 - \Delta^2}}{\Delta}, \qquad \tau_N = \frac{1}{1 + Z^2}. \tag{S4}$$

Here, Δ is the superconducting gap magnitude, Z is the dimensionless barrier strength characterizing the N-S interface, and Γ is a phenomenological broadening parameter that accounts for finite quasiparticle lifetime and other sources of smearing in the spectra. These quantities are treated as fitting parameters that encode the gap structure and junction transparency for a given PCAR contact.

Finite-temperature effects are incorporated by convolving the spectral conductance $\sigma(E)$ with the derivative of the Fermi-Dirac distribution. The experimentally measured differential conductance is then given by

$$G(V, T) = \int_{-\infty}^{\infty} \sigma(E) \left(-\frac{\partial f(E - eV)}{\partial E} \right) dE, \tag{S5}$$

which introduces thermal broadening of the sharp features present in the $T = 0$ spectrum. In the main text and other Supplementary sections, we extend this basic BTK framework to include multiple superconducting gaps and, where appropriate, anisotropic or mixed-parity order parameters; in those cases, the total conductance is obtained by summing the contributions from each gap with appropriate weighting factors.

S1.1.2. BTK Models for Anisotropic s-wave, p-wave, and $d_{x^2-y^2}$-wave Pairings

To explore different possible superconducting pairing states beyond a conventional isotropic s-wave gap, we employ extended BTK models that incorporate anisotropic s-wave, p-wave, and $d_{x^2-y^2}$-wave order parameters [2]. In the standard isotropic s-wave case, the superconducting gap magnitude Δ is independent of the quasiparticle incidence angle at the normal to superconductor interface, and the conductance is fully determined by the gap amplitude, barrier strength Z, and broadening parameter Γ [Sec. S1.1].

By contrast, for anisotropic pairing states, the gap depends on the angle of incidence θ, which modifies both the Andreev and normal reflection probabilities entering the BTK formula. In our simulations, the angular dependence of the pairing potential is modeled as

$$|\Delta(\theta)| = \Delta_0\big(1 + A\cos\theta\big) \qquad \text{(anisotropic } s \text{ wave)}, \tag{S6}$$

$$\Delta(\theta) = \Delta_0 \cos\theta \qquad (p \text{ wave}), \tag{S7}$$

$$\Delta(\theta) = \Delta_0 \cos(2\theta) \qquad (d_{x^2-y^2} \text{ wave}), \tag{S8}$$

where Δ_0 is the characteristic gap scale and A is a dimensionless anisotropy parameter for the s-wave case. For $A = 0$, the anisotropic s-wave model reduces to the isotropic s wave, whereas finite A introduces angular modulation of the gap magnitude without producing sign changes. In contrast, the p-wave and $d_{x^2-y^2}$-wave forms change sign as a function of θ and therefore support nodal or sign-changing order parameters typical of unconventional superconductivity.

S1.1.3. Extended BTK Model for Multi-Gap Pairing

In the T_d phase, we employ an extended BTK model for multi-gap s-wave superconductivity [1, 2]. Within this approach, the total normalized conductance G_{total} is modeled as a weighted sum of the contributions from each gap:

$$G_{\text{total}}(V) = wG_1(V) + (1 - w)G_2(V), \tag{S9}$$

where $G_i(V)$ is the conductance for the i-th pairing channel and w is the weighting factor. As discussed in the main text, the temperature-dependent PCAR spectra in the T_d phase are well reproduced by a two-gap s-wave BTK model within this framework, whereas in the $1T'$ phase a unique description requires the inclusion of an additional p-wave component.

S1.2. Experimental Data and Analysis of Multi-Gap Superconductivity in the T_d Phase

S1.2.1. Comparison between Single and Two-Gap BTK Fits to PCAR Data at 0.7 GPa

The multi-gap nature of superconductivity in the high-pressure T_d phase is further examined through temperature-dependent PCAR measurements at 0.7 GPa. At $T < T_c$, the normalized differential conductance spectra exhibit pronounced double peaks [Fig. S1(a)], which at first glance are compatible with a single isotropic s-wave gap. Indeed, as shown by the red curve in Fig. S1(a), a conventional one-gap s-wave BTK fit captures the overall shape of the spectrum reasonably well.

However, as shown in Fig. S1(b), the application of a magnetic field clearly reveals a pronounced two-gap feature, as marked by black arrows. A more quantitative BTK analysis demonstrates that the spectra are consistently reproduced with higher fidelity by a two-gap s-wave model, as illustrated by the blue dashed curve in Fig. S1(a) and by the systematic temperature evolution in Fig. 2(c) of the main text. At 0.7 GPa and 0.3 K, for example, the best two-gap BTK fit yields superconducting gaps $\Delta_1 \simeq 0.40$ meV and $\Delta_2 \simeq 0.18$ meV. The corresponding gap-to-T_c ratios, $2\bar{\Delta}_i/k_B T_c$, are $\simeq 3.2$ for the larger gap and $\simeq 1.4$ for the smaller gap, indicating that the smaller gap is comparatively weakly coupled and most likely induced via interband pairing interactions rather than determining T_c on its own. The full set of BTK fitting parameters for the single-gap and two-gap models, including the barrier strength Z, weighting factor w, and broadening parameter Γ, is summarized in Table S1.

Type	T (K)	Z	w (weighting factor)	$\Delta_{s,large}$ (meV)	$\Delta_{s,small}$ (meV)	Γ
s-wave (one-gap)	0.3	0.5	N/A	0.4	N/A	0.12
s+s wave (two-gap)	0.3	0.5	0.5	0.4	0.18	0.12

Table S1. Fitting parameters for single-gap and two-gap s-wave BTK models used to analyze the normalized differential conductance of $MoTe_2$ at 0.3 K and 0.7 GPa in the T_d phase. Here, T is the measurement temperature, Z is the dimensionless barrier strength, w is the weighting factor between the two gaps, $\Delta_{s,\mathrm{large}}$ and $\Delta_{s,\mathrm{small}}$ are the large and small superconducting gaps, and Γ is the quasiparticle broadening parameter.

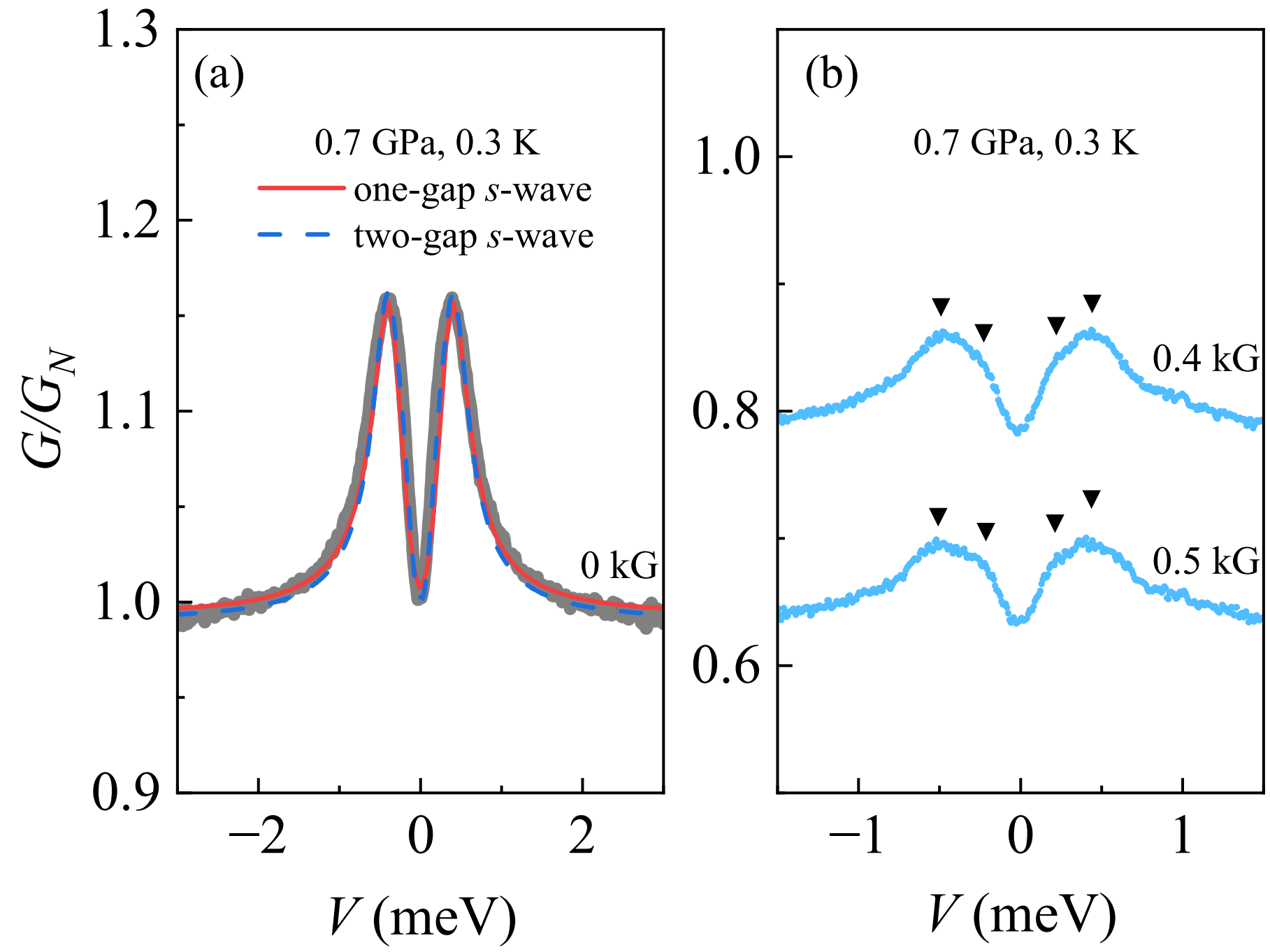

Fig. S1. **(a)** Normalized differential conductance of $MoTe_2$ at 0.7 GPa in the T_d phase, referenced to the 3.8 K data and measured at 0.3 K and 0 kG. Gray symbols denote the experimental PCAR spectrum, while the red solid and blue dashed curves represent the best least-squares fits obtained using single-gap and two-gap s-wave BTK models, respectively. The parameters are given in Table S1. **(b)** Normalized differential conductance of $MoTe_2$ under magnetic fields. At 0.4 and 0.5 kG, the two-gap feature becomes pronounced (black arrows), necessitating a two-gap scenario.

S1.2.2. Temperature- and Field-Dependent BTK Fitting Parameters at 0.7 GPa

To quantitatively characterize the two-gap superconductivity in the T_d phase at 0.7 GPa, we performed systematic BTK fits to the normalized differential conductance as a function of temperature and magnetic field. All spectra were analyzed within the same two-gap s-wave BTK framework used in Secs. S1.1.1-S1.1.3, with the barrier strength Z, weighting factor w, and quasiparticle broadening parameter Γ constrained to remain nearly constant across each series.

The temperature evolution of the fitting parameters at 0.7 GPa is summarized in Table S2. The larger gap $\Delta_{s,\mathrm{large}}$ stays close to $\sim$0.40 meV from 0.3 K up to about 1.5 K and then decreases toward zero as T approaches T_c, whereas the smaller gap $\Delta_{s,\mathrm{small}}$ is rapidly suppressed and essentially vanishes above $\sim$2 K. This contrasting behavior of the two gaps, together with the nearly temperature-independent Z, w, and Γ, is a hallmark of multiband superconductivity with finite interband coupling.

The magnetic-field evolution of the BTK parameters at 0.3 K and 0.7 GPa is presented in Table S3. With increasing field, both gaps are progressively reduced: $\Delta_{s,\mathrm{large}}$ decreases from $\sim$0.40 meV at zero field to zero near 2 kG, while $\Delta_{s,\mathrm{small}}$ is even more fragile and is already suppressed around 1 kG. At the same time, the broadening parameter Γ increases, reflecting enhanced pair breaking and quasiparticle scattering in the mixed state. These systematic trends in Tables S2 and S3, together with the PCAR spectra and H_{c2} analysis, provide a consistent and quantitative description of the two-gap s-wave superconductivity in high-pressure T_d-$MoTe_2$.

Type	T (K)	Z	w (weighting factor)	$\Delta_{s,\mathrm{large}}$ (meV)	$\Delta_{s,\mathrm{small}}$ (meV)	Γ
s+s wave (two-gap)	0.3	0.5	0.5	0.40	0.18	0.12
	0.9	0.5	0.5	0.40	0.10	0.12
	1.5	0.5	0.5	0.40	0.04	0.12
	1.8	0.5	0.5	0.37	0.02	0.12
	2.1	0.5	0.5	0.33	0.00	0.12
	2.5	0.5	0.5	0.22	0.00	0.12
	2.9	0.5	0.5	0.08	0.00	0.12
	3.3	0.5	0.5	0.00	0.00	0.12

Table S2. Temperature-dependent fitting parameters of the two-gap s-wave BTK model for the normalized differential conductance of $MoTe_2$ at 0.7 GPa in the T_d phase. Here, Z is the dimensionless barrier strength, w is the weighting factor between the two gaps, and Γ is the quasiparticle broadening parameter.

Type	$\mu_0 H$ (kG)	Z	w (weighting factor)	$\Delta_{s,\mathrm{large}}$ (meV)	$\Delta_{s,\mathrm{small}}$ (meV)	Γ
s+s wave (two-gap)	0.0	0.5	0.5	0.40	0.18	0.12
	0.2	0.5	0.5	0.40	0.16	0.13
	0.4	0.5	0.5	0.39	0.15	0.20
	0.5	0.5	0.5	0.37	0.13	0.22
	1.0	0.5	0.5	0.25	0.00	0.22
	2.0	0.5	0.5	0.00	0.00	0.22

Table S3. Magnetic-field-dependent fitting parameters of the two-gap s-wave BTK model for the normalized differential conductance of $MoTe_2$ at 0.3 K and 0.7 GPa in the T_d phase. As in Table S2, Z denotes the dimensionless barrier strength, w the weighting factor between the two gaps, and Γ the quasiparticle broadening parameter.

S1.2.3. Werthamer-Helfand-Hohenberg (WHH) Analysis of the Upper Critical Field

We analyze the temperature dependence of the upper critical field $H_{c2}(T)$ at 0.7 GPa within the Werthamer-Helfand-Hohenberg (WHH) framework for multi-gap superconductors [3–5]. Figure S2 shows the reduced upper critical field $h = H/H_{c2}(0)$ as a function of the reduced temperature $t = T/T_c$, together with the best fits obtained from single-gap (dashed line) and two-gap (solid line) WHH calculations.

In the absence of strong interband scattering, the reduced field $h(t)$ for a two-gap superconductor obeys

$$a_0 \left[\ln(t) + U\left(\frac{\tilde{h}}{t}\right)\right]\left[\ln(t) + U\left(\frac{\eta\tilde{h}}{t}\right)\right] + a_1\left[\ln(t) + U\left(\frac{\tilde{h}}{t}\right)\right] + a_2\left[\ln(t) + U\left(\frac{\eta\tilde{h}}{t}\right)\right] = 0 \quad \text{(S10)}$$

where $\tilde{h} = hD_1/2\Phi_0$, $U(x) = \psi(x + 1/2) - \psi(x)$, and $\psi(x)$ is the digamma function. The coefficients are given by $a_0 = 2(\lambda_{11}\lambda_{22} - \lambda_{12}\lambda_{21})$, $a_1 = 1 + (\lambda_{11} - \lambda_{22})/\lambda_0$, $a_2 = 1 - (\lambda_{11} - \lambda_{22})/\lambda_0$, and $\lambda_0 = [(\lambda_{11} - \lambda_{22})^2 + 4\lambda_{12}\lambda_{21}]^{1/2}$. Here λ_{ii} and λ_{ij} denote the intra- and interband BCS coupling constants $(i \neq j)$, D_k is the in-plane diffusivity of band k, and $\eta = D_2/D_1$ [3, 4].

Using this formalism, we find that the single-gap WHH curve systematically underestimates H_{c2} near T_c and fails to reproduce the pronounced negative curvature of $H_{c2}(T)$ [dashed line in Fig. S2]. In contrast, a two-gap WHH fit with interband coupling strengths $\lambda_{11} = 0.38$, $\lambda_{22} = 0.36$, and $\lambda_{12} = \lambda_{21} = 0.02$ (solid line) provides an excellent description of $h(t)$ over the entire temperature range, capturing both the initial slope close to T_c and the low-temperature behavior. The characteristic downward curvature of $H_{c2}(T)$ near T_c is therefore naturally explained by multi-gap effects rather than by a single-gap scenario.

Disorder-induced enhancements of H_{c2}, often observed in low-quality samples [6], are unlikely to play a dominant role here. Our $MoTe_2$ crystals exhibit a large residual resistivity ratio $\rho_{300\mathrm{K}}/\rho_{2\mathrm{K}} \approx 240$, indicative of high sample quality. Taken together with previous μSR and PCAR studies [7, 8], the two-gap WHH analysis in the T_d phase provides strong evidence that superconductivity in high-pressure T_d-$MoTe_2$ is intrinsically of multi-gap character.

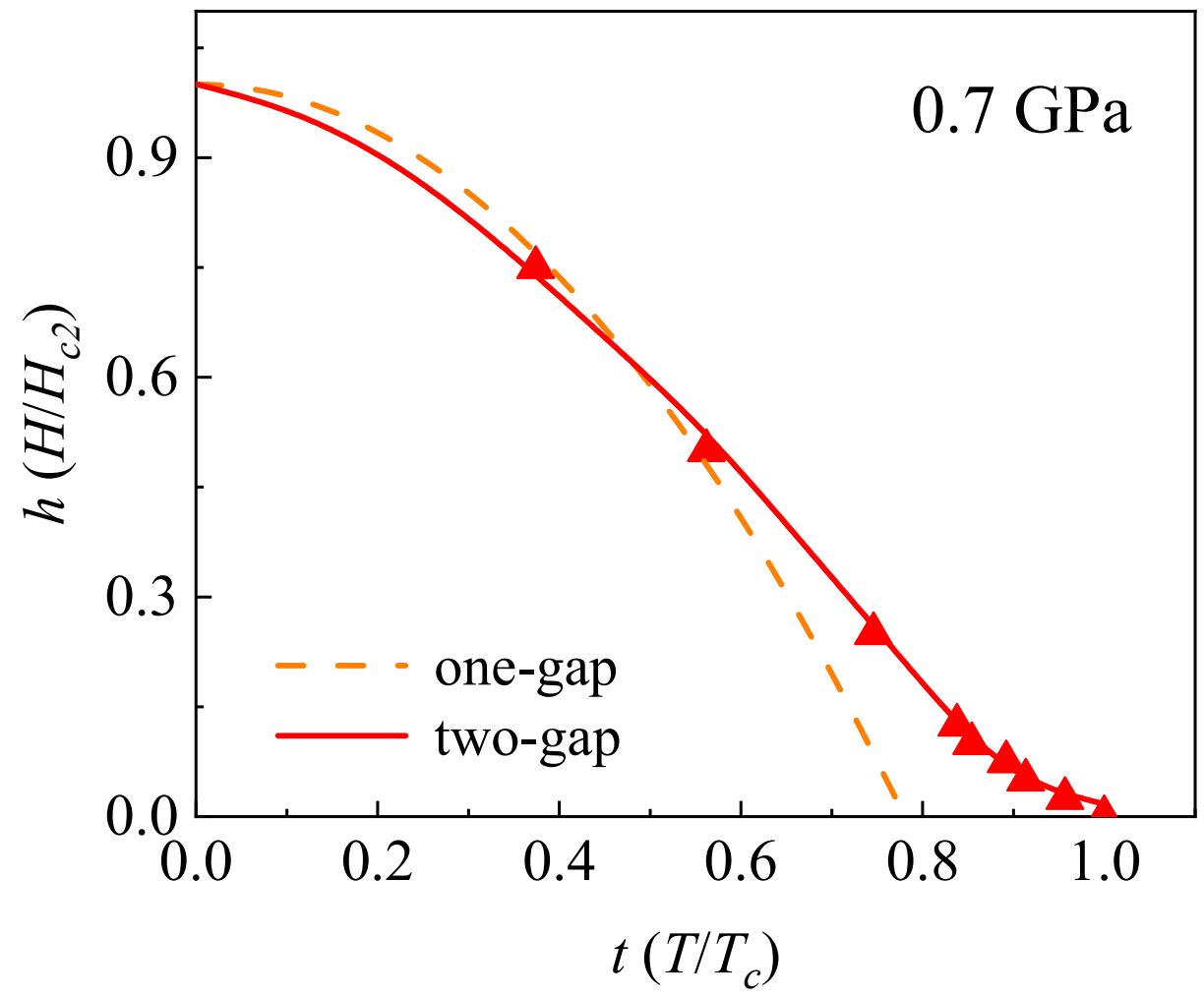

Fig. S2. Two-gap WHH analysis of the upper critical field at 0.7 GPa. Reduced upper critical field $h = H/H_{c2}(0)$ plotted as a function of reduced temperature $t = T/T_c$ (symbols). Dashed and solid lines represent the best fits obtained from single-band and two-band WHH calculations, respectively. The pronounced negative curvature of $H_{c2}(T)$ near T_c, which is well captured only by the two-gap WHH curve, indicates a multi-gap superconducting state in this pressure regime.

S1.2.4. Alternative PCAR Evidence for Two-Gap Superconductivity at 1.2 GPa

To verify that the multi-gap superconductivity in the T_d phase is not specific to a particular pressure setting, we performed additional PCAR measurements at a higher pressure of 1.2 GPa. Figures S3(a-e) summarize the representative spectra and the corresponding two-gap analysis.

Figure S3(a) shows the temperature evolution of the normalized differential conductance at 1.2 GPa, referenced to the 3.8 K normal-state spectrum. Pronounced double-peak structures persist up to temperatures close to T_c, and all spectra are well described by a two-gap s-wave BTK model (black curves). As at 0.7 GPa, a single-gap fit systematically fails to capture the detailed lineshapes, confirming that the two-gap structure is robust against pressure.

The magnetic-field dependence of the conductance at 0.3 K is presented in Figs. S3(b,c). With increasing field, the conductance peaks develop clear two gap-edge features, highlighted by black arrowheads in Fig. S3(c), reflecting the distinct field responses of the large and small gaps. These features are again faithfully reproduced by the two-gap BTK fits, and the spectra remain incompatible with a single-gap scenario.

From the BTK analysis, we extract the temperature and field dependence of the two superconducting gaps, shown in Figs. S3(d) and (e), respectively. In Fig. S3(d), the solid and dashed lines represent the solutions of the two-gap BCS gap equations with and without interband coupling using the theoretical calculations based on multi-gap model for a type-II Weyl-semimetal-derived superconducting system [see detailed derivation in Sec. S2]. As in the 0.7 GPa case, finite interband coupling is required to reproduce the long tail of the smaller gap toward T_c. Figure S3(e) displays the magnetic-field dependence of the gap amplitudes, which approximately follow the theoretical calculations based on the multi-gap model for a type-II Weyl-semimetal-derived superconducting system. Solid lines correspond to the case where both gaps share a common H_{c2}, while dashed lines assume distinct values of H_{c2} for the large and small gaps. The better agreement with the latter indicates different upper critical fields for the two bands.

Overall, the persistence of two distinct gaps and their characteristic temperature and field evolution at 1.2 GPa demonstrate that the multi-gap superconducting state in T_d-$MoTe_2$ is robust over a finite pressure range, consistent with the behavior reported at 0.7 GPa in the main text and with previous μSR and PCAR studies.

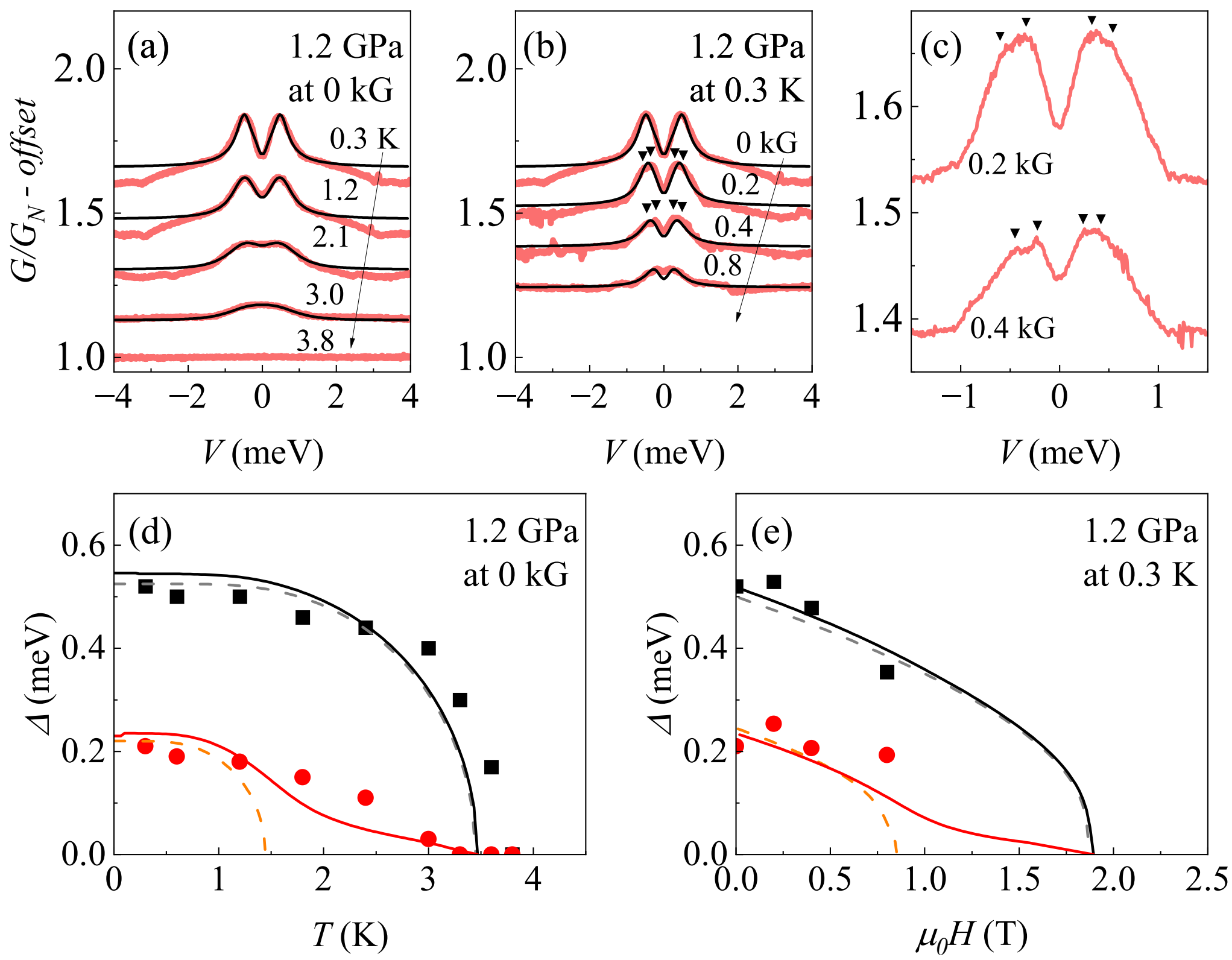

Fig. S3. Alternative experimental data for $MoTe_2$ in the T_d phase at 1.2 GPa. **(a)** Temperature evolution of the normalized differential conductance, referenced to the 3.8 K data. Black curves are best fits using a two-gap s-wave BTK model; spectra are vertically offset for clarity with respect to the 0.3 K, 0 kG curve. **(b)** Magnetic-field evolution of the normalized differential conductance at 0.3 K. The two gap-edge features in the conductance peaks, indicated by black arrowheads, reflect the distinct field responses of the large and small gaps; curves are vertically shifted for clarity. **(c)** Magnetic-field evolution of the normalized differential conductance at 0.3 K, focusing on the two-gap feature (black arrows), is shown for 0.2 kG and 0.4 kG. **(d)** Temperature dependence of the two superconducting gaps. Solid and dashed lines represent theoretical calculations based on a coupled multi-gap model for a type-II Weyl-semimetal-derived superconducting system. The pronounced tail of the smaller gap persisting up to T_c arises from finite interband coupling between the two superconducting bands. **(e)** Magnetic-field dependence of the two superconducting gaps. The gap amplitudes follow closely the theoretical calculations based on the multi-gap model for a type-II Weyl-semimetal-derived superconducting system.

S1.2.5. Temperature- and Field-Dependent BTK Fitting Parameters at 1.2 GPa

To confirm that the multi-gap superconductivity in the T_d phase is robust against pressure, we have carried out a detailed BTK analysis of the PCAR spectra at a higher pressure of 1.2 GPa. As in the 0.7 GPa case [Tables S2 and S3], all spectra are analyzed within the two-gap s-wave BTK framework, using a single set of barrier and broadening parameters for each series.

The temperature-dependent fitting parameters are summarized in Table S4. The barrier strength $Z \simeq 0.51$, weighting factor $w \simeq 0.46$, and broadening $\Gamma = 0.14$ remain essentially constant over the entire temperature range, indicating that the evolution of the spectra is governed primarily by the superconducting gaps. The larger gap, $\Delta_{s,\mathrm{large}}$, stays close to ~ 0.5 meV at low temperatures and gradually decreases toward zero as T approaches $T_c \approx 3.8$ K, while the smaller gap, $\Delta_{s,\mathrm{small}}$, is reduced more rapidly and vanishes already near $T \sim 3.3$ K. This contrasting behavior of the two gaps mirrors the trends observed at 0.7 GPa and again points to multiband superconductivity with finite interband coupling.

The magnetic-field-dependent BTK parameters at 0.3 K and 1.2 GPa are listed in Table S5. With increasing field, both $\Delta_{s,\mathrm{large}}$ and $\Delta_{s,\mathrm{small}}$ are progressively suppressed, while the quasiparticle broadening Γ increases from 0.14 to 0.21, reflecting enhanced pair breaking and scattering in the mixed state. The fact that two distinct gaps with different field dependences are required to describe the spectra reinforces the conclusion drawn from Fig. S3 that the two-gap structure persists at 1.2 GPa and is thus robust over a finite pressure range in the T_d phase of $MoTe_2$.

Type	T (K)	Z	w (weighting factor)	$\Delta_{s,large}$ (meV)	$\Delta_{s,small}$ (meV)	Γ
s+s wave (two-gap)	0.3	0.51	0.46	0.51	0.17	0.14
	0.6	0.51	0.46	0.5	0.17	0.14
	1.2	0.51	0.46	0.5	0.17	0.14
	1.8	0.51	0.46	0.46	0.15	0.14
	2.4	0.51	0.46	0.44	0.11	0.14
	3.0	0.51	0.46	0.4	0.03	0.14
	3.3	0.51	0.46	0.3	0	0.14
	3.6	0.51	0.46	0.17	0	0.14
	3.8	0.51	0.46	0	0	0.14

Table S4. Temperature-dependent fitting parameters of the two-gap s-wave BTK model for the normalized differential conductance of $MoTe_2$ at 1.2 GPa in the T_d phase. As in Table S2, Z denotes the dimensionless barrier strength, w the weighting factor between the two gaps, and Γ the quasiparticle broadening parameter.

Type	$\mu_0 H$ (kG)	Z	w (weighting factor)	$\Delta_{s,large}$ (meV)	$\Delta_{s,small}$ (meV)	Γ
s+s wave (two-gap)	0	0.51	0.46	0.51	0.17	0.14
	0.2	0.51	0.46	0.49	0.1	0.16
	0.4	0.51	0.46	0.36	0.15	0.21
	0.8	0.51	0.46	0.25	0.14	0.21

Table S5. Magnetic-field-dependent fitting parameters of the two-gap s-wave BTK model for the normalized differential conductance of $MoTe_2$ at 0.3 K and 1.2 GPa in the T_d phase. As in Table S2, Z denotes the dimensionless barrier strength, w the weighting factor between the two gaps, and Γ the quasiparticle broadening parameter.

S1.2.6. Revealing Multi-Gap Pairing via Magnetic-Field-Dependent PCAR

The multi-gap character of superconductivity in the high-pressure T_d phase becomes particularly evident in the magnetic-field-dependent PCAR spectra. As shown in Fig. 2(d), the two-gap-edge features of each conductance peak are progressively enhanced upon increasing the magnetic field up to 0.5 kG, as indicated by the black arrowheads. A similar field evolution is observed at 1.2 GPa [Figs. S3(b,c)], demonstrating that the characteristic two-gap response is robust over a wide pressure range.

The distinct field evolution indicates that the two SC gaps respond differently to an applied magnetic field, as expected for a multi-gap superconductor with finite but weak interband coupling. At low fields, the smaller gap is suppressed more rapidly, whereas at higher fields it remains finite by being effectively dragged by the larger gap through interband coupling. This mechanism prevents the smaller gap from closing at its own intrinsic H_{c2} and instead produces a long-tailed suppression with increasing field. Consequently, the two-gap character becomes more visible under field, even when the two peaks are only weakly resolved at zero field. Field-dependent gap magnitudes at 0.7 and 1.2 GPa [Fig. 2(f) and Fig. S3(e)] consistently support this picture and suggest that the interband coupling may be slightly stronger at 1.2 GPa than at 0.7 GPa.

The magnetic-field dependence of the spectra is fully consistent with this interpretation. The multi-gap character already becomes apparent under relatively small fields ($\mu_0 H \lesssim 0.5$ kG), as highlighted by the arrows in Fig. 2(d), and the evolution of the two gaps with magnetic field is summarized in Fig. 2(e). The corresponding gap magnitudes $\Delta_i(H)$ are shown in Fig. 2(f), where the solid and dashed lines are the theoretical calculations based on the multi-gap model for a type-II Weyl-semimetal-derived superconducting system. In one case the two gaps are constrained to share a common H_{c2}, while in the other they are allowed to have distinct H_{c2} values for comparison. The latter description provides a better account of the data and is naturally understood in terms of two bands with different orbital limiting fields that are nevertheless coupled by interband pairing.

Similar field-induced enhancement of multi-gap signatures has been reported in other superconductors, including MgB_2 and the iron-based compound $Ba(Fe,Co)_2As_2$ [9–11]. Our field-dependent BTK analysis yields gap magnitudes, with $\bar{\Delta}_1(0) \approx 0.4$ meV and $\bar{\Delta}_2(0) \approx 0.18$ meV at 0.7 GPa and 0.3 K, consistent with previous μSR measurements reporting $\bar{\Delta}_1(0) \approx 0.43$ meV and $\bar{\Delta}_2(0) \approx 0.11$ meV at 1 GPa in T_d-$MoTe_2$ [7, 12]. Taken together, the magnetic-field-dependent PCAR spectra and the extracted gap magnitudes provide strong, complementary evidence for intrinsic two-gap superconductivity in high-pressure T_d-$MoTe_2$.

This multi-gap nature, together with finite interband coupling, has important implications for the effective BCS coupling ratios extracted from experiments. In multi-gap superconductors, deviations from the

single-band BCS value $2\Delta/k_BT_c = 3.53$ are an expected consequence of interband coupling. Near T_c, the linearized gap equations show that the transition temperature is governed by a collective pairing eigenmode associated with the largest eigenvalue of the interband coupling matrix, which can enhance T_c beyond the intraband pairing strength of any individual band. By contrast, at zero temperature the nonlinear gap equations redistribute the pairing amplitude among the bands, such that a stronger band partially sustains the gap of a weaker one. As a result, the individual zero-temperature gap magnitudes are reduced relative to the enhanced T_c, and the ratios $2\Delta_i(0)/k_BT_c$ can naturally fall below the single-band BCS value without implying anomalously weak pairing, even within a weak-coupling BCS framework [11].

Consistent with this picture, reduced effective BCS ratios have been reported in a variety of multi-gap superconductors, including $Nb_2Pd_{0.81}S_5$ (1.6 and 3.1) [13], V_3Si (2.0 and 2.8) [14], $FeSe_{0.5}Te_{0.5}$ (2.96) [15], $LuNi_2B_2C$ (3.2) [16], and 122-type iron pnictide superconductors (3.1) [17]. These examples illustrate that reduced BCS ratios are a common feature of multi-gap superconductivity arising from interband coupling and band-dependent pairing strengths.

S1.3. BTK Analysis for Superconducting Pairing Symmetries in the 1T′ Phase

S1.3.1. Quantitative Comparison of BTK Fits for Different Pairing Symmetries

To quantify how well different superconducting pairing symmetries reproduce the PCAR spectra in the high-pressure 1T′ phase, we perform a systematic residual analysis of the BTK fits shown in Fig. S4. For this analysis we use the same PCAR spectrum as in the main-text Fig. 3(b), i.e., the 1T′-phase data taken at 2.2 GPa and $T = 0.3$ K, and fit it with three representative models: anisotropic s-wave, d-wave, and mixed $s{+}p$-wave pairing.

Figures S4(a-c) compare the experimental conductance (purple curves) with the corresponding BTK calculations (black curves) for the anisotropic s-, d-, and $s{+}p$-wave scenarios, respectively. The blue-shaded areas at the bottom of each panel represent the squared deviation between the data and the BTK fit,

$$\Delta G^2(V) = \left[G_{\text{data}}(V) - G_{\text{BTK}}(V)\right]^2 , \tag{S11}$$

which we use as a measure of the local fitting error. The total fitting error for each pairing symmetry is then obtained by integrating $\Delta G^2(V)$ over bias voltage,

$$\text{Error} = \int dV \Delta G^2(V), \tag{S12}$$

and the resulting values are summarized in Fig. S4(d).

Within the class of BTK models we have considered, the PCAR spectra in the 1T′ phase are most consistently captured by the $s{+}p$-wave gap [Fig. 3(c) of the main text]. Both Fig. 3(c) and Fig. S4 demonstrate that the deviations between the BTK fits and the experimental data are substantially smaller for the $s{+}p$-wave model than for the anisotropic s-wave or d-wave models. In particular, the integrated squared error in Fig. S4(d) is minimized for the $s{+}p$-wave fit, highlighting that the mixed $s{+}p$-wave pairing provides the best description of the 2.2 GPa, 1T′-phase, 0.3 K spectrum of Fig. 3(b). This residual analysis therefore supports the conclusion that the single peak near zero bias in the 1T′ phase is most naturally accounted for by an $s{+}p$-wave superconducting state, rather than by a purely anisotropic s-wave or d-wave gap.

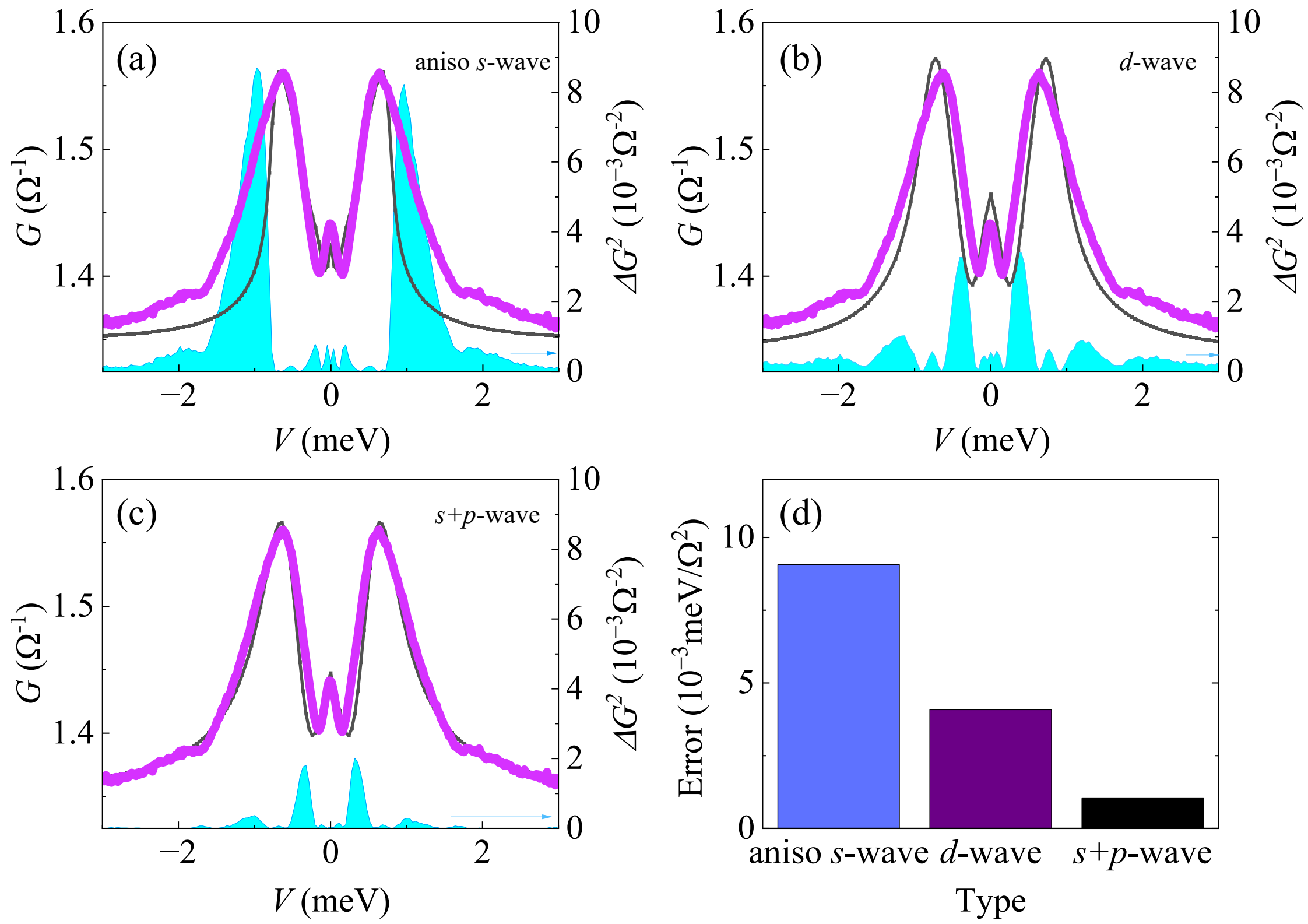

Fig. S4. Analysis of BTK fits for different pairing symmetries in the high-pressure 1T′ phase. **(a-c)** BTK fits (black curves) to the same PCAR spectrum as in the main-text Fig. 3(b), measured in the 1T′ phase at 2.2 GPa and $T = 0.3$ K, for anisotropic s-, d-, and mixed s+p-wave pairing, respectively, compared with the experimental conductance (purple curves). The anisotropy parameters α for the anisotropic s-wave, d-wave, and s+p-wave cases are $\pi/4$, $\pi/14$, and 0, respectively. The blue-shaded regions at the bottom of each panel represent the squared deviation between the data and the fit, $\Delta G^2(V) = \left(G_{\text{data}} - G_{\text{BTK}}\right)^2$, providing a measure of the local fitting error. **(d)** Integrated fitting error, $\int dV\, \Delta G^2(V)$, for each pairing symmetry, showing that the s+p-wave scenario yields the smallest total deviation. This quantitative comparison demonstrates that, within the BTK models considered, the mixed s+p-wave pairing most consistently reproduces the 1T′-phase PCAR spectrum, supporting the s+p-wave interpretation of the single peak near zero bias.

S1.3.2. BTK comparison of s- and p-wave pairing in the 1T′ phase

To elucidate the pairing symmetry in the pressure-induced 1T′ phase, we first analyze the PCAR spectra at 2.2 GPa and various temperatures using the simplest single-gap BTK models. Figures S5(a) and (b) show the temperature evolution of the normalized differential conductance, measured at zero field in the 1T′ phase, fitted with a conventional isotropic s-wave gap and a single-gap p-wave gap, respectively. Colored curves denote the experimental spectra, while black lines represent the corresponding BTK calculations. The fits are obtained by adjusting the gap amplitude, barrier strength, and quasiparticle broadening at each temperature, with the tunneling angle fixed to $\alpha = \pi/1.35$ in the p-wave case, where α denotes the orientation of the soft point contact relative to the gap anisotropy.

A fully gapped isotropic s-wave state fails to reproduce the pronounced zero-bias peak observed at low temperatures [Fig. S5(a)]. Although the overall magnitude of the coherence peaks can be matched, the s-wave BTK curve always underestimates the conductance around zero bias and cannot account for the sharp peak near zero bias. The single-gap p-wave model [Fig. S5(b)] better captures the enhancement of conductance near zero bias but simultaneously distorts the lineshape around the side peaks, leading to systematic discrepancies in the intermediate-bias region. Furthermore, the finite α employed in this analysis is distinct from the experimental condition in which α = 0.

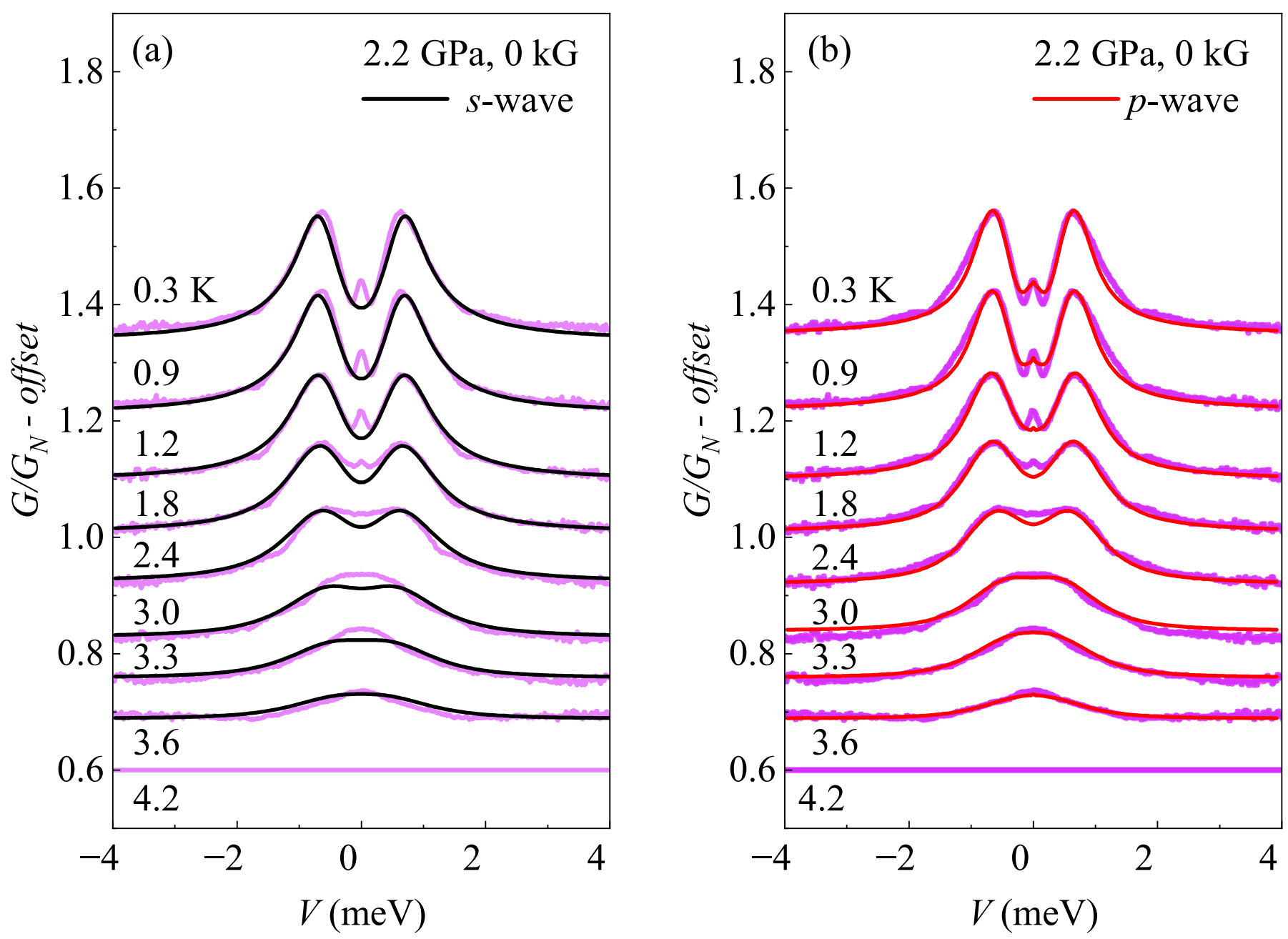

Fig. S5. BTK comparison of s- and p-wave pairing in the pressure-induced 1T′ phase. **(a,b)** Temperature evolution of the normalized differential conductance in the 1T′ phase at 2.2 GPa and zero field, fitted with (a) a single-gap isotropic s-wave BTK model and (b) a single-gap p-wave BTK model with tunneling angle fixed to $\alpha = \pi/1.35$. Colored curves denote the experimental spectra, while solid black lines represent the corresponding BTK fits.

S1.3.3. BTK Simulations of Competing Gap Symmetries in the 1T′ Phase

To test whether the single peak at zero-bias and accompanying side peaks observed in the pressure-induced 1T′ phase can be explained by alternative pairing symmetries within a conventional BTK framework, we simulated PCAR spectra for anisotropic s-wave and $d_{x^2-y^2}$-wave gaps and compared them with the experimental data at 2.2 GPa [Fig. S6].

Figure S6(a) displays BTK simulations for an anisotropic s-wave order parameter, calculated for several polar tunneling angles $\alpha = \pi/2$, $\pi/4$, $\pi/8$, and $\pi/16$, where α denotes the orientation of the soft point contact relative to the gap anisotropy. Along the nodal direction of the anisotropic s-wave gap ($\alpha = \pi/4$, red curve), the simulated conductance shows a modest single peak near zero-bias superimposed between double conductance maxima. For the other angles ($\alpha = \pi/2$, $\pi/8$, and $\pi/16$), the spectra are dominated by well-defined double peaks with only a weak or absent zero-bias enhancement. Figure S6(b) directly compares the $\alpha = \pi/4$ simulation with the temperature-dependent PCAR spectra at 2.2 GPa. Although the nodal anisotropic s-wave state can generate a peak at zero-bias, it fails to reproduce the detailed line shape, temperature evolution, and relative spectral weight as accurately as the s+p-wave BTK fits in Fig. 3(b) of the main text, disfavoring a purely anisotropic s-wave interpretation.

Figure S6(c) shows BTK simulations for a $d_{x^2-y^2}$-wave gap for the same set of tunneling angles. Along the antinodal direction ($\alpha = 0$, black dashed curve), the simulated spectrum exhibits conventional double coherence peaks without a pronounced peak at zero-bias, mimicking a nodeless s-wave-like response. Closer to the nodal direction ($\alpha = \pi/4$, red curve), the $d_{x^2-y^2}$-wave simulation produces a strong ZBP accompanied by characteristic dips near the gap edges. However, for all intermediate angles between nodal and antinodal directions, the simulated line shapes deviate significantly from the measured spectra, as summarized in Fig. S6(d), where the temperature-dependent PCAR spectra are compared with a representative $d_{x^2-y^2}$-wave fit at $\alpha = \pi/14$. It should be noted that, in all cases considered here, the finite α used in this analysis deviates from the experimental condition of $\alpha = 0$.

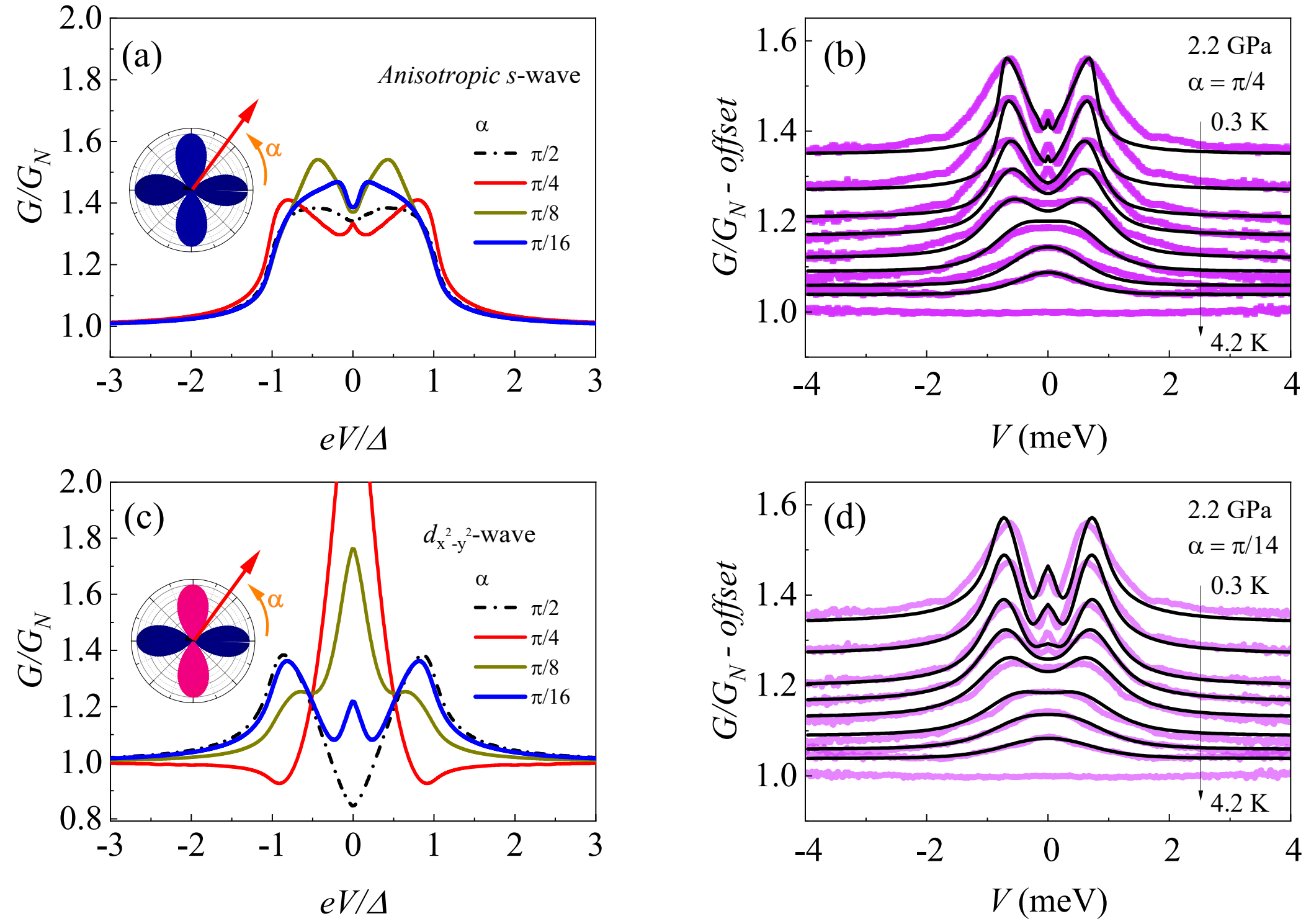

Fig. S6. Blonder-Tinkham-Klapwijk (BTK) analysis of competing gap symmetries in the 1T′ phase. **(a,c)** Simulated PCAR spectra for (a) anisotropic s-wave and (c) $d_{x^2-y^2}$-wave pairing states, calculated for several polar tunneling angles $\alpha = \pi/2, \pi/4, \pi/8$, and $\pi/16$. The angle α denotes the orientation of the soft point contact relative to the gap structure. In (a), $Z = 0.42$ and $\Gamma = 0$ meV, while in (c), $Z = 0.69$ and $\Gamma = 0.13$ meV. **(b,d)** Temperature-dependent PCAR spectra at 2.2 GPa (colored curves) fitted by (b) anisotropic s-wave and (d) $d_{x^2-y^2}$-wave BTK models. In (b) and (d), the representative tunneling angles are $\alpha = \pi/4$ and $\alpha = \pi/14$, respectively. For clarity, all spectra are vertically offset relative to the 0.3 K curve.

S1.3.4. BTK Parameterization of s+p-Wave Superconductivity at 2.2 GPa

To quantify the mixed-parity superconducting state proposed for the 1T′ phase, we performed systematic BTK fits of the PCAR spectra at 2.2 GPa using an s+p-wave gap function, as in Fig. 3(b) of the main text. The resulting fitting parameters are summarized in Table S6 for the temperature evolution and in Table S7 for the magnetic-field evolution.

Table S6 lists the temperature dependence of the s- and p-wave gap amplitudes, Δ_s and Δ_p, together with the effective barrier strength Z, weighting factor w, and quasiparticle broadening parameter Γ. The parameters $Z \simeq 0.6$, $w \simeq 0.5$, and $\Gamma \simeq 0.24$ remain essentially constant over the entire temperature range, indicating that the evolution of the spectra is governed predominantly by the gaps themselves. At low temperatures (0.3-1.2 K), both components are sizable, with $\Delta_s \approx 0.6$ meV and $\Delta_p \approx 0.4$ meV, showing that the p-wave contribution is comparable in magnitude to the s-wave part. As T approaches $T_c \approx 4.2$ K, the two gaps decrease in tandem and eventually vanish together, reflecting a strongly mixed order parameter in which the s- and p-wave components are locked by spin-orbit coupling rather than forming two independent bands.

Table S7 summarizes the magnetic-field dependence of the BTK parameters at 2.2 GPa and 0.3 K. As the field is increased from zero toward H_{c2}, both Δ_p and Δ_s are gradually suppressed, while the effective broadenings Γ_p and Γ_s increase, consistent with enhanced pair breaking and quasiparticle scattering in the mixed state. Importantly, the two gaps exhibit a similar field dependence and remain of comparable magnitude throughout, indicating that the ZBCP and finite-bias peaks arise from a single condensate rather than two decoupled order parameters. This behavior is in line with the analysis of the field-dependent spectra in Figs. 4(d-f) of the main text and with the systematic failure of purely anisotropic s-wave and d-wave BTK models [Figs. S4 and S6]. Overall, the temperature and field evolution of the s+p-wave BTK parameters provide quantitative support for an unconventional SC state in the pressure-induced 1T′ phase of $MoTe_2$.

Type	T (K)	Z	w (weighting factor)	Δ_p (meV)	Δ_s (meV)	Γ
s+p wave	0.3	0.6	0.5	0.4	0.6	0.24
	0.6	0.6	0.5	0.4	0.6	0.24
	1.2	0.6	0.5	0.41	0.59	0.24
	1.8	0.6	0.5	0.43	0.57	0.24
	2.4	0.6	0.5	0.4	0.5	0.24
	3	0.6	0.5	0.37	0.4	0.24
	3.3	0.6	0.5	0.3	0.28	0.24
	3.6	0.6	0.5	0.22	0.23	0.24
	4.2	0.6	0.5	0.01	0.01	0.24

Table S6. Temperature-dependent fitting parameters of the s+p-wave BTK model for the normalized differential conductance of $MoTe_2$ at 2.2 GPa in the 1T′ phase. As in Table S2, Z denotes the dimensionless barrier strength, w the weighting factor between the two gaps, and Γ the quasiparticle broadening parameter.

Type	T (K)	Z	w (weighting factor)	Δ_p (meV)	Δ_s (meV)	Γ_p/Γ_s
s+p wave	0.3	0.6	0.5	0.4	0.6	0.24
	0.3	0.6	0.5	0.40	0.58	0.31/0.33
	0.3	0.6	0.5	0.39	0.49	0.35/0.37
	0.3	0.6	0.5	0.37	0.39	0.37/0.39
	0.3	0.6	0.5	0.26	0.26	0.39/0.4
	0.3	0.6	0.5	0	0	0

Table S7. Magnetic-field-dependent fitting parameters of the s+p-wave BTK model for the normalized differential conductance of $MoTe_2$ at 2.2 GPa in the 1T′ phase. As in Table S2, Z denotes the dimensionless barrier strength, w the weighting factor between the two gaps, and Γ the quasiparticle broadening parameter.

S1.4. Systematic Exclusion of Extrinsic Mechanisms for the Single Peak near Zero-Bias

S1.4.1. Broad Zero-Bias Conductance Hump in the Thermal Regime

Not all point contacts operate in the ballistic or diffusive spectroscopic regime assumed in standard PCAR analysis. When the effective contact diameter becomes large compared with the inelastic mean free path, or when the current density approaches the critical current density J_c of the constriction, the junction crosses over to a thermal regime. In this limit, the injected power $P = IV$ is dissipated locally, leading to a substantial increase of the electronic temperature in the contact region above the bath temperature. As a result, the differential conductance is governed primarily by self-heating and critical-current effects rather than by the energy-dependent quasiparticle spectrum of the superconductor.

A characteristic signature of the thermal regime is that the measured differential conductance is dominated by self-heating and critical-current effects rather than by spectroscopic Andreev-reflection processes. In this limit, the injected power is dissipated locally and the conductance primarily reflects the temperature dependence of the bulk transport properties of the sample. As a consequence, the zero-bias conductance $G(0)$ is expected to vary smoothly and monotonically with temperature, closely tracking the inverse of the electrical resistivity. In bias-dependent spectra, this behavior is typically manifested as a broad zero-bias conductance maximum accompanied by a suppression of conductance at finite bias, instead of sharp, well-resolved gap-edge features. Close to the critical current, the voltage drop across the constriction becomes strongly nonlinear and the conductance tends to saturate, resulting in a wide hump centered at $V = 0$ with no clear spectroscopic structure. Similar non-spectroscopic zero-bias anomalies arising from point-contact heating and critical-current effects have been reported in superconductors with relatively large normal-state resistivity [18, 19].

Figure S7(a) shows a representative example of such a non-spectroscopic thermal regime observed in the present system. At $T = 0.3$ K, the differential conductance $G(V)$ exhibits a wide zero-bias hump flanked by shallow minima at finite bias and lacks well-defined Andreev-reflection features. This line shape is fully consistent with transport dominated by local heating and critical-current effects, and is clearly distinct from the spectra analyzed in the main text.

Further evidence distinguishing thermal and spectroscopic regimes is provided by the temperature dependence of the zero-bias conductance. Figure S7(b) compares $G(0)$ with the electrical resistivity $\rho(T)$. While the resistivity (solid symbols, right ordinate) decreases monotonically upon cooling through the superconducting transition, the zero-bias conductance (open symbols, left ordinate) exhibits a qualitatively different behavior. In particular, $G(0)$ does not evolve monotonically with decreasing temperature below

T_c, in clear contrast to the inverse-resistivity scaling expected for point-contact spectroscopy operating in the thermal regime. In the thermal limit, $G(0)$ is governed primarily by local Joule heating and therefore closely tracks the temperature dependence of the bulk resistivity. The pronounced deviation observed here, especially the non-monotonic evolution of $G(0)$ below T_c, indicates that the point contact is not operating in the thermal regime and that the zero-bias conductance reflects intrinsic spectroscopic contributions.

Consistent with this conclusion, the magnetic-field-dependent PCAR spectra at 2.2 GPa and 0.3 K shown in Fig. 4(d) do not display a broad, field-sensitive zero-bias hump, ruling out a heating-induced origin of the zero-bias conductance peak discussed in the main text.

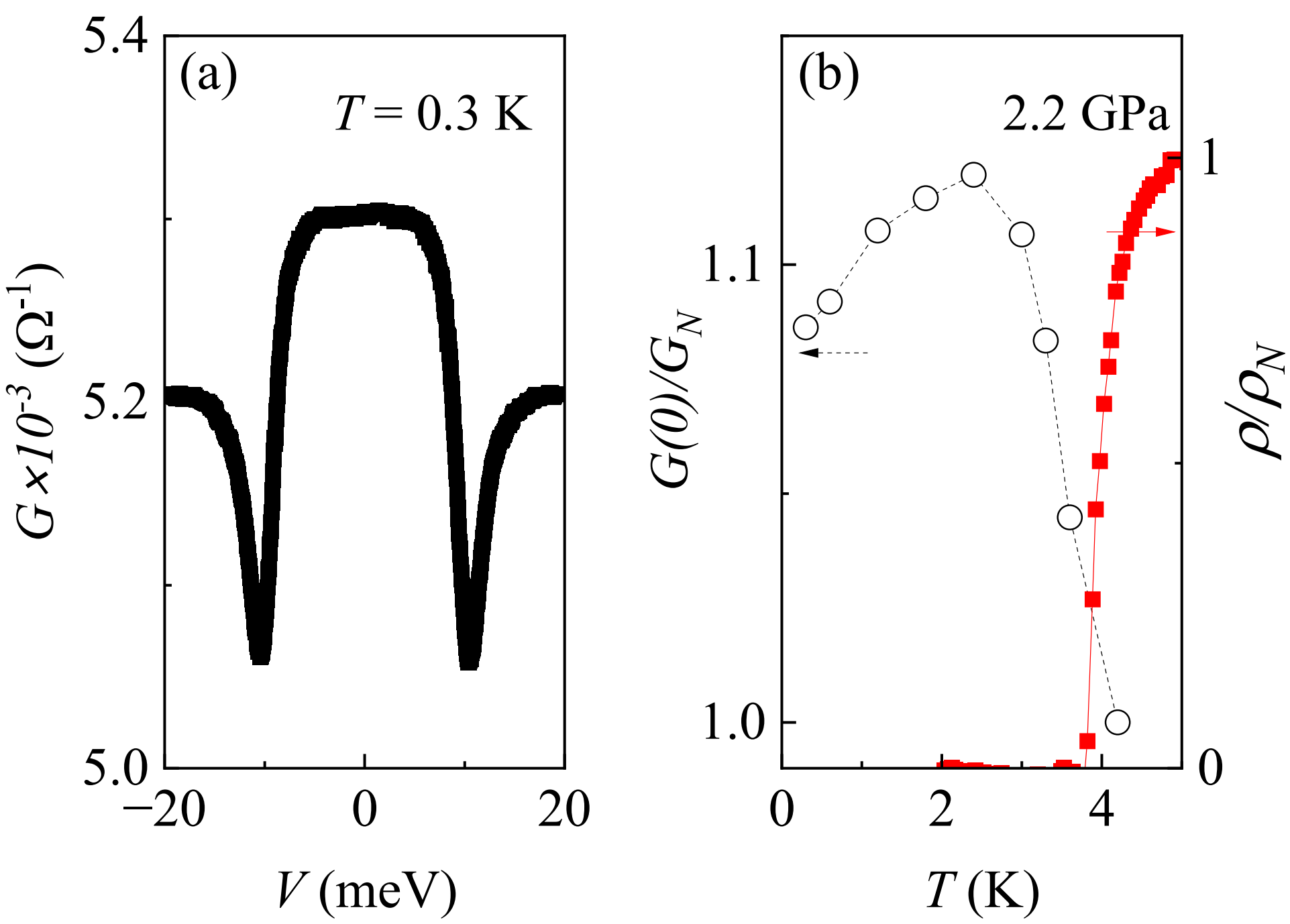

Fig. S7. **(a)** Differential conductance $G(V)$ measured at $T = 0.3$ K for a point contact operating in the thermal (non-spectroscopic) regime. The spectrum exhibits a broad zero-bias conductance maximum with suppressed conductance at finite bias and no sharp Andreev-reflection features, consistent with transport dominated by local heating and critical-current effects rather than ballistic point-contact spectroscopy. **(b)** Zero-bias differential conductance $G(0)$ (open symbols) shown as a function of temperature (left ordinate), together with the electrical resistivity $\rho(T)$ (solid symbols, right ordinate) magnified near the superconducting phase transition.

S1.4.2. Excluding a Two-Gap s-wave Origin of the Single Peak near Zero-Bias

A second possible explanation for a single near-zero-bias conductance peak is a two-gap s-wave scenario in which one of the gaps is extremely small. In such a case, the larger gap produces the broad Andreev-reflection shoulders at finite bias, while the very small gap generates a narrow low-energy feature that can merge into an apparent single peak near zero bias at elevated temperatures. To quantify this possibility, we have performed BTK simulations for a model with two isotropic s-wave gaps, $\Delta_{\rm large}$ and $\Delta_{\rm small}$, using representative barrier strength Z and quasiparticle broadening Γ.

Figure S8(a) shows the simulated conductance spectra at $T = 3.3$ K for various gap ratios $\Delta_{\rm large}/\Delta_{\rm small}$. A pronounced single peak near zero bias appears only when the gap disparity is extreme: the larger gap must exceed the smaller one by at least a factor of ~ 7 at 3.3 K in order for the inner peaks associated with $\Delta_{\rm small}$ to overlap and form an apparently single central maximum. For more modest gap ratios, the two-gap structure remains clearly resolved, with distinct shoulders or multiple peaks in $G(V)$.

The role of temperature is illustrated in Fig. S8(b), which plots the temperature evolution of the spectra for a fixed ratio $\Delta_{\rm large}/\Delta_{\rm small} = 10$. At low temperatures, the two sets of Andreev features from the large and small gaps are well separated. Only when the temperature is significantly increased does thermal smearing wash out the structure associated with the small gap and produce a single, relatively sharp peak at $V = 0$. Thus, a two-gap s-wave scenario requires both an unrealistically large gap disparity and elevated temperatures to mimic a single near-zero-bias peak.

Our experimental parameters are far from this regime. At 2.2 GPa, the BTK analysis of the point-contact spectra yields a p-wave gap that is approximately 75% of the s-wave gap [Fig. 4(c)], implying a gap ratio of only $\Delta_s/\Delta_p \approx 1.3$ rather than $\gtrsim 7$. Moreover, the key measurements are performed down to $T = 0.3$ K, where thermal broadening is minimal. Under these conditions, our simulations show that a purely two-gap s-wave model cannot generate a sharp single zero-bias peak [see Fig. S8(b)]. Consistent with this conclusion, extensive BTK fits [Figs. S4-S6] systematically fail to reproduce the observed spectra, whereas the mixed s+p-wave model provides the most satisfactory and physically consistent description. We therefore exclude multi-gap s-wave superconductivity as the origin of the zero-bias peak reported in the main text.

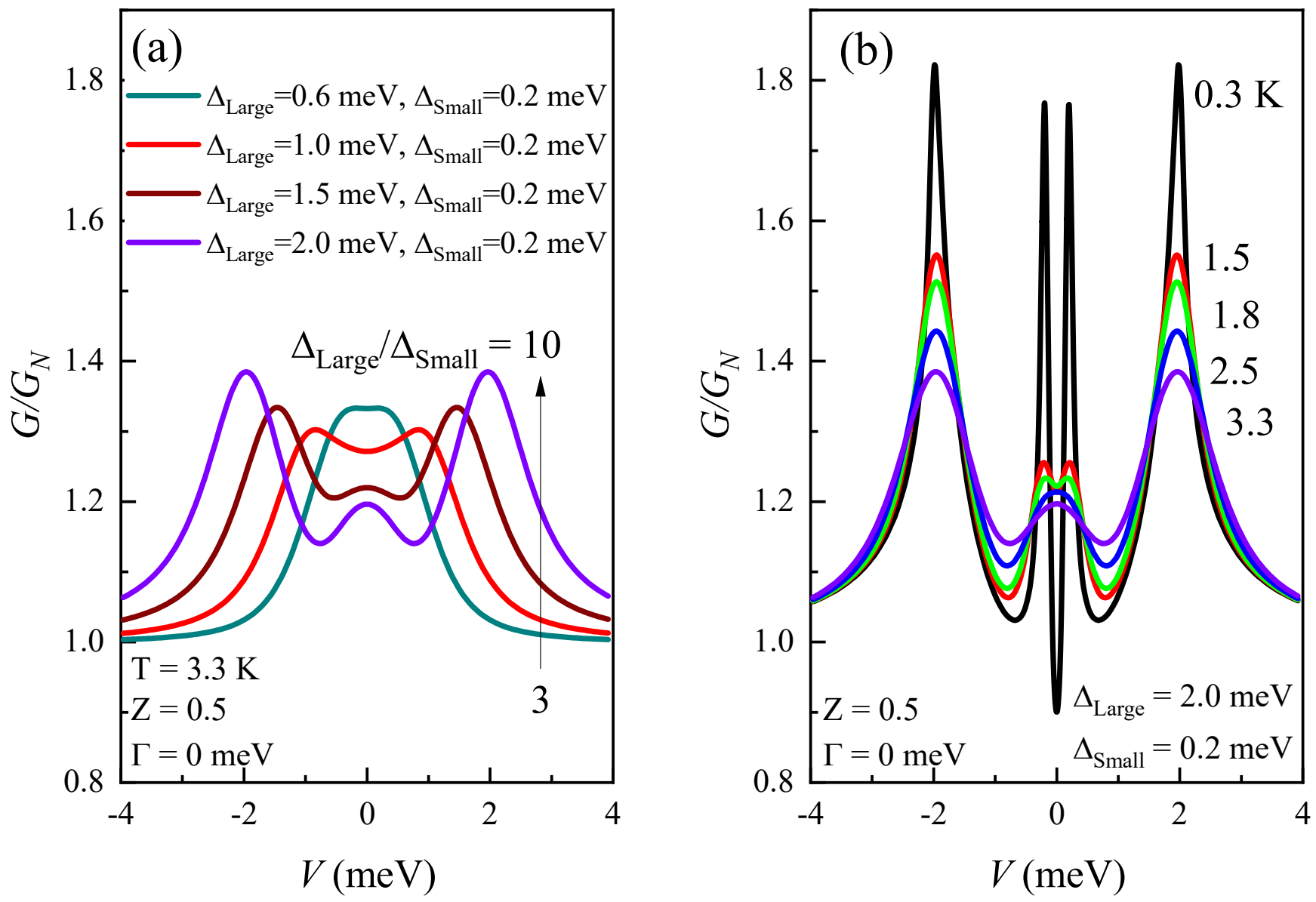

Fig. S8. **(a)** Simulated BTK spectra for a superconductor with two isotropic s-wave gaps, showing the evolution of the normalized conductance G/G_N at $T = 3.3$ K as the gap ratio $\Delta_{\rm large}/\Delta_{\rm small}$ is increased. A pronounced single peak near zero bias emerges only for very large gap disparities. **(b)** Simulated temperature dependence of the two-gap s-wave spectra for a fixed ratio $\Delta_{\rm large}/\Delta_{\rm small} = 10$, demonstrating that the appearance of a zero-bias peak requires both an extreme gap ratio and elevated temperature, conditions that are not realized in the present experiments.

S1.4.3. Ruling out impurity-induced origins of the Single Peak near Zero-Bias

A sharp peak at zero bias can have qualitatively different origins depending on the superconducting gap structure. In a nodal superconductor, such a feature may be an intrinsic manifestation of Andreev bound states associated with sign-changing order parameters. By contrast, in a fully gapped (nodeless) superconductor, a zero-bias peak is more naturally attributed to in-gap bound states generated by magnetic impurities or other extrinsic defects [see Sec. S1.4.3 and Refs. [20–23]]. A key distinction between these scenarios is their response to a magnetic field: impurity-induced bound states undergo Zeeman splitting into spin-up and spin-down levels, causing the zero-bias peak to split into two finite-bias peaks, whereas an intrinsic Andreev-reflection feature in a nodal state is expected to remain unsplit over a finite field range.

In our high-pressure point-contact spectra, the zero-bias peak observed in the 1T′ phase remains sharp and unsplit under applied magnetic fields, and is only gradually suppressed in amplitude across the entire field range [Figs. 4(d,e)]. This behavior rules out a magnetic-impurity origin, which would generically produce a Zeeman doublet instead of a single central peak. Moreover, unlike experiments where only a single broad maximum is present, our spectra in Figs. 4(a,d) display two well-resolved Andreev conductance peaks in addition to the zero-bias feature. The coexistence of these double peaks with a narrow central enhancement is indicative of a clean, nearly ballistic point contact with strong Andreev reflection, making it unlikely that the observed structures are dominated by disorder or non-ideal junction effects.

To further test the robustness and universality of the zero-bias peak, we have examined multiple point contacts on different crystals and at different pressures. Figure S9 compares spectra from two representative contacts. At 0.6 GPa in the T_d phase [Fig. S9(a)], only the double Andreev peaks characteristic of multi-gap s-wave superconductivity are observed; there is no enhancement at zero bias. By contrast, at 1.6 GPa in the 1T′ phase [Fig. S9(b)], a clear zero-bias peak develops between the two Andreev maxima, even though the overall contact conditions and interface quality differ from those of the main dataset in Fig. 3(a). Similar behavior is found for other contacts at 1.4 GPa and 2.2 GPa: while the relative height of the central peak can vary from contact to contact, a finite zero-bias enhancement consistently appears once the system is driven into the 1T′ phase.

This systematic pressure dependence is difficult to reconcile with an impurity-driven or sample-specific mechanism. Extrinsic impurities or accidental junction configurations would not be expected to track the structural phase boundary. However, as summarized in Fig. 3(a), there is no zero-bias peak throughout the entire T_d phase, and it appears only after the transition into the 1T′ phase is completed. The emergence of the zero-bias peak therefore closely correlates with the pressure-induced change in the normal-state topology from a type-II Weyl semimetal to a higher-order topological phase, and with the accompanying evolution of

the pairing symmetry in the superconducting state. The modest contact-to-contact variations in peak height are naturally interpreted as differences in interface transparency, while the very presence of the zero-bias peak is a robust property governed by the intrinsic high-pressure superconducting state of $MoTe_2$.

Taken together, the absence of Zeeman splitting, the selective appearance of the peak only in the 1T′ phase, and its reproducibility across independent contacts and samples provide compelling evidence that the zero-bias conductance peak is an intrinsic, pressure-tuned feature of the superconducting state rather than an artifact of impurities or non-ideal junction configurations.

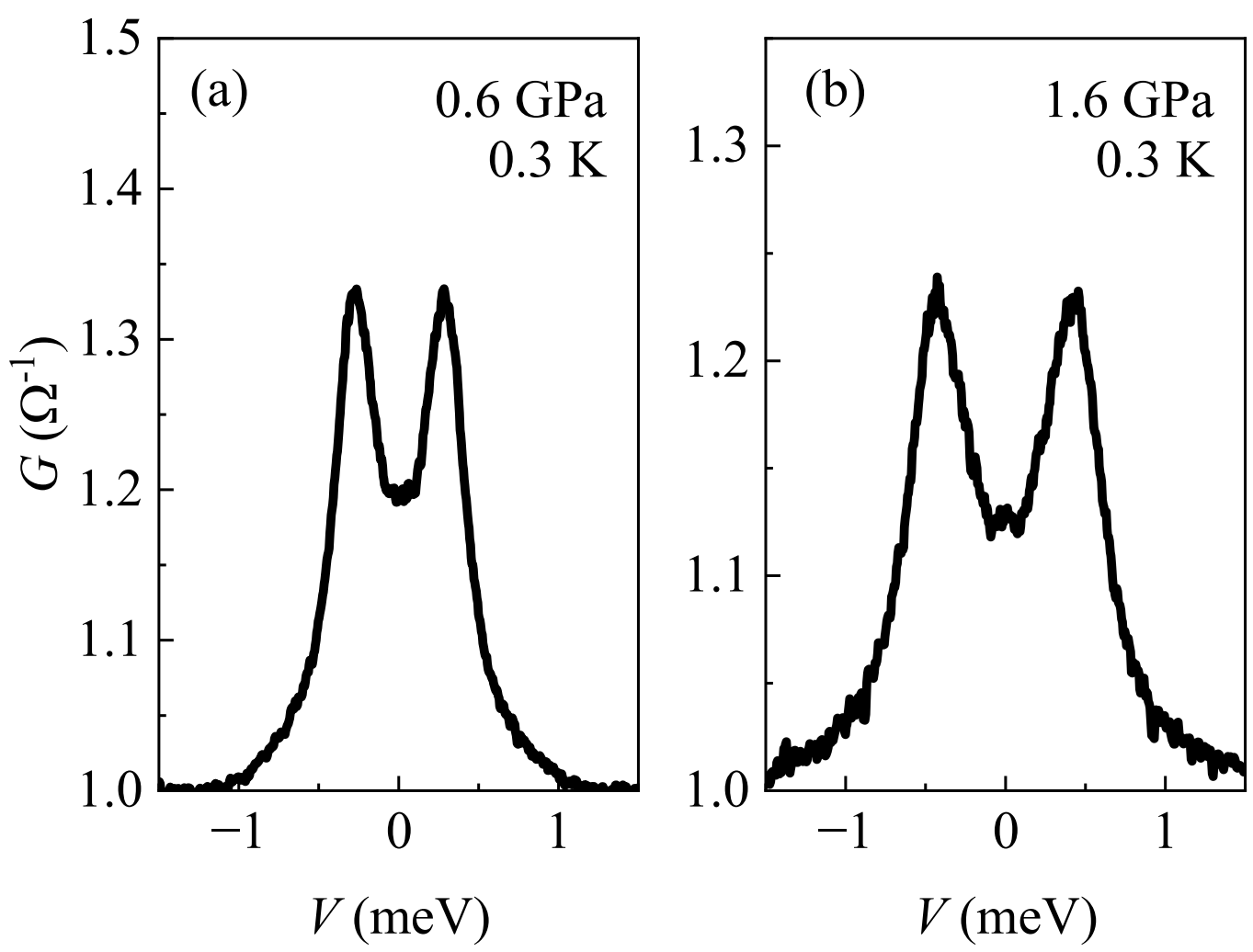

Fig. S9. Evolution of the single near-zero-bias conductance peak between the double Andreev maxima for two different point contacts. **(a)** Spectrum at 0.6 GPa in the T_d phase, showing only the double Andreev peaks expected for multi-gap s-wave superconductivity and no enhancement at zero bias. **(b)** Spectrum at 1.6 GPa in the 1T′ phase, where a sharp zero-bias peak develops between the two Andreev peaks. The appearance of the central peak only after the pressure-driven phase transition from T_d to 1T′ transition supports its intrinsic origin in the high-pressure superconducting state.

S2. THEORETICAL ANALYSIS FOR SUPERCONDUCTING T_d PHASE

In this section, we present the theoretical framework used to interpret the superconducting properties of $MoTe_2$ in the T_d phase. Following previous work [24, 25], we start from a high-symmetry T_0 phase from which the experimentally relevant low-temperature structures T_d and $1T'$ are obtained via distinct symmetry-breaking distortions. In the T_0 phase the low-energy electronic structure near the Γ point consists of a conventional parabolic band and a Dirac cone, and the system respects both inversion and time-reversal symmetries, i.e., it is PT symmetric. Breaking inversion symmetry drives the T_0 to T_d phase transition and converts the Dirac cone into a pair of Weyl cones, whereas a shear-mode distortion produces the centrosymmetric $1T'$ phase.

In the following, we first construct minimal tight-binding (TB) models for the normal-state band structures of the T_0 and T_d phases, emphasizing the emergence of Weyl cones from an underlying Dirac semimetal. We then use this normal-state description as the starting point for an effective Bogoliubov-de Gennes (BdG) theory of superconductivity, from which we derive coupled two-band gap equations for the T_d phase and analyze the temperature and magnetic-field dependence of the superconducting order parameters for comparison with the experimental data in the main text.

S2.1. Minimal tight-binding model for the T_d phase

To capture the essential normal-state physics in the T_d phase we construct a minimal TB model in which a parabolic band hybridizes with a Dirac-like band inherited from the T_0 phase. We begin with a TB Hamiltonian proposed previously for the centrosymmetric T_0 phase of $MoTe_2$ [24–27]. This model serves as a convenient starting point from which the noncentrosymmetric T_d phase and its Weyl cones can be obtained by introducing inversion-symmetry-breaking terms.

In the PT-symmetric T_0 structure, the low-energy Dirac Hamiltonian near the Γ point can be written schematically as

$$H_{\text{Dirac}}(\mathbf{k}) = (m_0 + \Sigma_{i=x,y,z} t_i \cos k_i)\, \tau_z + \lambda_x \sin k_x \tau_y \mu_y + \lambda_z \sin k_z \tau_x, \tag{S13}$$

where τ_i and μ_j are Pauli matrices acting on different orbital degrees of freedom, while σ_k acts on spin. The parameters t_i denote nearest-neighbor hopping amplitudes, and λ_j encode spin-orbit-coupled hoppings. The corresponding dispersion is

$$E_{\text{Dirac}} = \pm\sqrt{(m_0 + \Sigma_{i=x,y,z} t_i \cos k_i)^2 + \lambda_x^2 \sin^2 k_x + \lambda_z^2 \sin^2 k_z},$$

which realizes a Dirac semimetal with two Dirac points located along the k_y axis. This Dirac system respects inversion symmetry $P = \tau_z$ and time-reversal symmetry $\Theta = i\tau_z\sigma_y K$, where K denotes complex conjugation.

S2.1.1. Inversion-symmetry breaking and Weyl nodes in the T_d phase

When inversion symmetry is broken and the system enters the orthorhombic noncentrosymmetric T_d phase, density-functional-theory (DFT) calculations show that the Dirac points split into four Weyl nodes located near the Fermi level [25, 27]. This splitting is driven by inversion-symmetry-breaking mass terms that lift the Kramers degeneracy and gap the Dirac cone along specific momentum directions. To capture this effect we introduce inversion breaking parameters $\delta_{x,z}$ (as listed in Table S8) and obtain an effective Weyl Hamiltonian

$$H^0_{\mathrm{W}}(\mathbf{k}) = (m_0 + \Sigma_{i=x,y,z} t_i \cos k_i)\,\tau_z + \lambda_x \sin k_x \tau_y \mu_y + \lambda_z \sin k_z \tau_x + \delta_x \tau_y \mu_y \sigma_z + \delta_z \tau_x \sigma_z. \quad \text{(S14)}$$

The symmetry-allowed inversion-breaking mass terms δ_i that gap the Dirac cone along the k_i direction ($i = x, z$) are summarized in Table S8. Notably, there is no symmetry-allowed mass term δ_y that simultaneously breaks inversion while preserving time-reversal symmetry. The band structure resulting from Eq. (S14) exhibits four Weyl cones with dispersion

$$E^0_{\text{Weyl}} = \pm\sqrt{(m_0 + \Sigma_{i=x,y,z} t_i \cos k_i)^2 + (\lambda_x \sin k_x \pm \delta_x)^2 + (\lambda_z \sin k_z \pm \delta_z)^2}.$$

DFT and experimental studies further establish that the T_d phase of $MoTe_2$ realizes a type-II Weyl semimetal, in which the Weyl cones are strongly tilted, and the Fermi surface becomes open rather than pointlike [25, 28–30]. To incorporate this effect, we add a momentum-dependent tilt term along the k_y direction and obtain the full Weyl Hamiltonian

$$\begin{aligned} H_{\mathrm{W}}(\mathbf{k}) \;=\; & (\alpha \cos k_y + \beta) + (m_0 + \Sigma_{i=x,y,z} t_i \cos k_i)\,\tau_z + \lambda_x \sin k_x \tau_y \mu_y + \lambda_z \sin k_z \tau_x \quad \text{(S15)} \\ & + \delta_x \tau_y \mu_y \sigma_z + \delta_z \tau_x \sigma_z, \end{aligned}$$

where α controls the magnitude of the tilt and β accounts for a shift of the Weyl nodes along k_y induced by inversion-breaking terms. The corresponding dispersion is

$$E_{\text{Weyl}} = (\alpha \cos k_y + \beta) \pm \sqrt{(m_0 + \Sigma_{i=x,y,z} t_i \cos k_i)^2 + (\lambda_x \sin k_x \pm \delta_x)^2 + (\lambda_z \sin k_z \pm \delta_z)^2}.$$

For sufficiently strong tilt, specifically when $\alpha > t_y$, the Weyl cones overtilt and the system enters the type-II regime [30]. In this work we use this minimal model as the normal-state starting point for constructing an effective Bogoliubov-de Gennes (BdG) description of superconductivity in the T_d phase.

The SC T_d phase is then modeled by a BdG Hamiltonian that combines the tilted Weyl band of Eq. (S15) with an additional parabolic band at the Γ point, both subject to s-wave pairing. This two-band setting naturally leads to multi-gap superconductivity, as discussed below.

Type	
δ_x	$\tau_y\sigma_0$, $\tau_y\mu_y\sigma_{x,y,z}$
δ_z	$\tau_x\sigma_{x,y,z}$, $\tau_x\mu_y\sigma_0$

Table S8. Matrix representations for possible inversion-symmetry-breaking mass terms δ_i for $i = x, z$ that split the Dirac cones into Weyl nodes. Here, τ_i and μ_j act on orbital degrees of freedom, and σ_k acts on spin.

S2.2. Coupled two-gap equation in the T$_d$ phase

To connect the minimal band-structure model of Sec. S2.1. to the experimentally observed evolution of the superconducting gaps, we derive a coupled two-band gap equation for the T$_d$ phase. For clarity, we retain only the Weyl cone closest to the Fermi level and include an additional parabolic band crossing the Fermi level. These two bands form the basis of a minimal two-gap superconducting model that captures the essential multi-gap behavior in the low-pressure T$_d$ phase.

We denote by $a_{k,s}$ and $c_{k,s}$ the annihilation operators for electrons with momentum k and spin s in the parabolic and Weyl bands, respectively. The mean-field pairing interaction is written as

$$H_{\text{int}} = U a^\dagger_{k,\uparrow} a^\dagger_{-k,\downarrow} a_{-k,\downarrow} a_{k,\uparrow} + V c^\dagger_{k,\uparrow} c^\dagger_{-k,\downarrow} c_{-k,\downarrow} c_{k,\uparrow} + W \left(a^\dagger_{k,\uparrow} a^\dagger_{-k,\downarrow} c_{-k,\downarrow} c_{k,\uparrow} + \text{h.c.} \right),$$

where U and V are the intraband pairing interactions for the parabolic and Weyl bands, respectively, and W describes the interband (proximity-type) coupling between them.

Introducing SC order parameters on each band, we define

$$\begin{aligned} \Delta_1 &= U \sum_k \left\langle a_{-k,\downarrow} a^\dagger_{k,\uparrow} \right\rangle + W \sum_k \left\langle c_{-k,\downarrow} c_{k,\uparrow} \right\rangle , \\ \Delta_2 &= V \sum_k \left\langle c_{-k,\downarrow} c^\dagger_{k,\uparrow} \right\rangle + W \sum_k \left\langle a_{-k,\downarrow} a_{k,\uparrow} \right\rangle , \end{aligned}$$

where Δ_1 and Δ_2 are the SC gaps associated with the parabolic band ($i = 1$) and the Weyl band ($i = 2$), respectively. For band dispersions measured from the chemical potential $\bar{\xi}_{k,i} = \lambda_{k,i} + \xi_{k,i}$ with $\lambda_{k,i} = -\lambda_{-k,i}$ and $\xi_{k,i} = \xi_{-k,i}$, the quasiparticle energies are given as $E_{k,i,\pm} = \lambda_{i,k} \pm E_{k,i} = \lambda_{i,k} \pm \sqrt{\xi^2_{k,i} + |\Delta_i|^2}$.

The mean-field free energy can be written as

$$F = \sum_{i,k,s} \left[\xi_{k,i} - \frac{1}{\beta} \ln \left(2 \cosh \frac{\beta E_{k,i,s}}{2} \right) \right] + \frac{V \left|\Delta_1\right|^2 + U \left|\Delta_2\right|^2 - 2W \text{Re} \Delta_1^* \Delta_2}{UV - W^2}, \tag{S16}$$

where $s = \pm$. Minimizing this free energy with respect to Δ_i^*, i.e., enforcing $\partial_{\Delta_i^*} F = 0$, yields the coupled self-consistent gap equations

$$\begin{aligned} \Delta_1 &= U \sum_{k,s} \frac{\Delta_1}{4sE_{k,1}} \tanh \frac{\beta E_{k,1,s}}{2} + W \sum_{k,s} \frac{\Delta_2}{4sE_{k,2}} \tanh \frac{\beta E_{k,2,s}}{2}, \\ \Delta_2 &= V \sum_{k,s} \frac{\Delta_2}{4sE_{k,2}} \tanh \frac{\beta E_{k,2,s}}{2} + W \sum_{k,s} \frac{\Delta_1}{4sE_{k,1}} \tanh \frac{\beta E_{k,1,s}}{2} \end{aligned}$$

We solve these equations numerically to obtain the temperature dependence of the two gaps $\Delta_1(T)$ and $\Delta_2(T)$ in the T$_d$ phase. The resulting gap functions reproduce the experimental temperature evolution extracted from the PCAR spectra [Fig. 2 of the main text], in particular, the long tail of the smaller gap up to T_c, which is naturally explained by the weak but finite interband coupling W.

To model the magnetic-field dependence, we include a Zeeman term B such that

$$\bar{\lambda}_{k,i} = \lambda_{k,i} - B.$$

Fixing the temperature at $T = 0.3$ K, we track the suppression of Δ_1 and Δ_2 with increasing B. The resulting behavior is in good agreement with the field evolution extracted from the PCAR spectra and is consistent with the phenomenological form $\Delta_i(H) \sim (H_{c2,i} - H)^{1/2}$ used in the main text.

For the 1T$'$ phase, where a p-wave component is induced via a bulk-to-surface proximity effect, we apply the above formalism to the parabolic band to determine the s-wave gap and then relate the induced p-wave amplitude to the s-wave order parameter through an analytically derived ratio. Due to the inversion symmetry of the system, $\lambda_{k,i} = 0$ for the 1T$'$ phase. The detailed relation between the proximity-induced p-wave pairing and the bulk s-wave gap is discussed in the next section.

S3. THEORETICAL ANALYSIS FOR SUPERCONDUCTING 1T′ PHASE

We now turn to the monoclinic 1T′ phase of $MoTe_2$, which has been proposed to host higher-order band topology. Our objective is to show how a second-order topological insulator (SOTI) in the normal state can, via a momentum-space proximity effect, give rise to a third-order topological superconductor (TOTSC) supporting Majorana corner states. To this end we first analyze a lattice SOTI Hamiltonian appropriate for the 1T′ structure and derive its hinge states both analytically and numerically. In the following sections we then incorporate superconducting pairings within a BdG framework, compute a $\mathbb{Z}_8$ bulk topological index, and explicitly derive Majorana corner modes using Jackiw-Rebbi-type arguments in both cylindrical and spherical geometries.

S3.1. Second-Order Topological Insulator: Normal State Model

To describe the 1T′ phase, we adopt a low-energy TB model in which the effect of the shear distortion relative to the high-symmetry T_0 structure is encoded through spin-orbit coupling (SOC) and symmetry-allowed mass terms [26, 27]. In the T_0 phase the low-energy spectrum near the Γ point consists of a parabolic band and a Dirac cone, and the system is PT symmetric. SOC gaps out the Dirac cone, while additional mass terms drive a double band inversion, realizing an SOTI [26, 27, 31]. The resulting SOTI band gives rise to surface states that form two copies of a helical Dirac cone with spin-momentum locking [27] [Fig. 5(a)], which in a finite geometry host one-dimensional gapless hinge states [Fig. 5(b)], a hallmark of higher-order topology [32]. Here we summarize the lattice Hamiltonian, its symmetries, and the emergence of hinge modes.

S3.1.1. Lattice SOTI Hamiltonian and Symmetries

We begin with the SOTI Hamiltonian introduced to describe the monoclinic 1T′ phase of $MoTe_2$ [26, 27]. The bulk Hamiltonian takes the form

$$\begin{aligned} H_{\text{SOTI}}(\mathbf{k}) \;=\; & \left(m_0 + \Sigma_{i=x,y,z} t_i \cos k_i\right) \tau_z + \lambda_x \sin k_x \tau_y \mu_y + \lambda_z \sin k_z \tau_x \\ & + \lambda_y \sin k_y \tau_y \mu_z \sigma_z + m_1 \tau_z \mu_z + m_2 \mu_x . \end{aligned} \tag{S17}$$

Here, τ_i and μ_j are Pauli matrices acting on two independent orbital subspaces, and σ_k acts on spin. The parameters t_i denote nearest-neighbor hoppings, while λ_j encode spin-orbit or orbital-dependent hoppings. The mass terms m_1 and m_2 are symmetry-allowed perturbations that play a crucial role in converting a topological insulator into an SOTI by gapping the surfaces while leaving hinge states gapless.

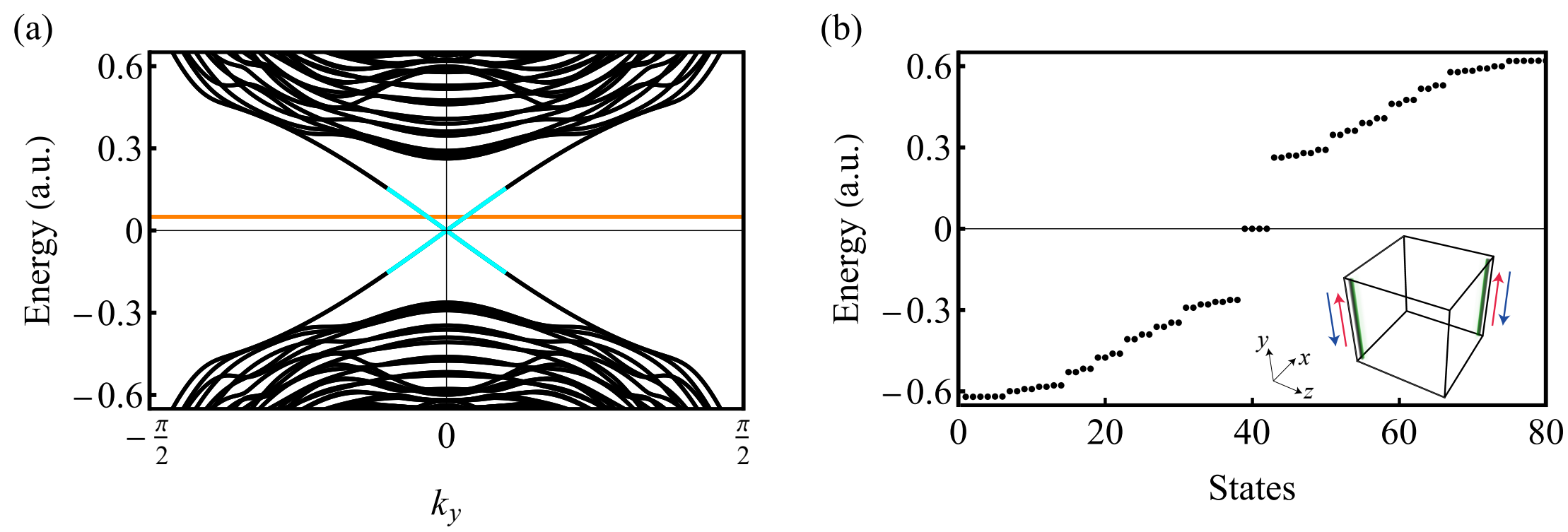

Fig. S10. **(a)** TB band structure (black lines) for a finite SOTI system with open boundaries along x and z and periodic boundary conditions along y. The orange horizontal line marks the chemical potential $\mu = 0.05$ used in Fig. 5 of the main text. Analytical hinge dispersions obtained from the surface theory (blue lines) overlap with the numerical results near zero energy. **(b)** Zoom-in of the eigenvalues around zero energy at $k_y = 0$. Four zero-energy states appear, corresponding to topological hinge modes, whose real-space probability distributions are shown in the inset (darker color indicates higher density). Each hinge hosts a Kramers' pair, leading to helical hinge channels. Parameters: $t_x = t_z = 1$, $t_y = 2$, $\lambda_x = \lambda_z = 1$, $\lambda_y = 0.5$, $m_0 = -3$, and $m_1 = m_2 = 0.3$.

The SOTI model respects inversion symmetry $P = \tau_z$, time-reversal symmetry $\Theta = i\tau_z\sigma_y K$, a twofold rotation $C_2 = i\tau_z\sigma_x$ about the y axis, and a corresponding mirror symmetry $\sigma_h = i\sigma_x$. These symmetries reflect the monoclinic crystal structure of 1T′-$MoTe_2$ and constrain both bulk topology and boundary states. The Hamiltonian therefore satisfies

$$
\begin{aligned}
P H_{\text{SOTI}}(\mathbf{k}) P^\dagger &= H(-\mathbf{k}), \\
\Theta H_{\text{SOTI}}(\mathbf{k}) \Theta^\dagger &= H(-\mathbf{k}), \\
C_2 H_{\text{SOTI}}(k_x, k_y, k_z) C_2^\dagger &= H(-k_x, k_y, -k_z), \\
\sigma_h H_{\text{SOTI}}(\mathbf{k}) \sigma_h^\dagger &= H(k_x, -k_y, k_z).
\end{aligned}
$$

When implemented on a finite lattice with open boundaries along x and z and periodic boundary conditions along y, this Hamiltonian hosts gapless hinge states localized along the y-directed edges, as shown in Fig. S10, and reported in previous studies [26, 27].

S3.1.2. Analytical Derivation of Topological Hinge States

To gain further analytical insight into the hinge modes of Fig. S10, we follow Ref. [33] and derive them from an effective surface theory. We consider a cylindrical geometry that is finite in x and z but periodic along y, so that k_y remains a good quantum number. Introducing cylindrical coordinates in the xz plane, we parametrize

$$(k_x, k_y, k_z) = (k_\perp \sin\phi + k_\parallel \cos\phi,\ k_y,\ k_\perp \cos\phi - k_\parallel \sin\phi),$$

where $k_\perp$ and $k_\parallel$ denote momenta normal and tangential to the surface, and ϕ is the polar angle in the xz plane.

Linearizing Eq. (S17) around the Γ point and replacing $k_\perp$ with $-i\partial_r$ for $\lambda_x = \lambda_z = 1$ leads to an effective Dirac equation $H_D = M(r)\tau_z - i\partial_r(\cos\phi\,\tau_x + \sin\phi\,\tau_y\mu_y)$, where $M(r)$ is a Dirac mass that changes sign across the surface. The bulk (r inside the cylinder) is topological and the outside region is trivial. We then look for surface-localized states of the form $|\psi_i\rangle = \mathcal{N}\exp[-\int dr\, M(r)]|u_i\rangle$, with $\mathcal{N}$ being a normalization constant and $|u_i\rangle$ an r-independent spinor. Projecting the bulk Hamiltonian onto these surface modes yields the effective surface Hamiltonian. The projected surface Hamiltonian $[H_{\text{Surf}}(\mathbf{k})]_{ij} = \langle\psi_i|H_{\text{SOTI}}(\mathbf{k})|\psi_j\rangle$ takes the form

$$\begin{aligned} H_{\text{Surf}}(\mathbf{k}) \ &= \ -k_\parallel\tau_y - (m_2\cos\phi - m_1\sin\phi)\,(\cos\phi\tau_x + \sin\phi\tau_z) \\ &\quad +\lambda_y \sin k_y\,(\sin\phi\tau_x - \cos\phi\tau_z)\,\sigma_z. \end{aligned} \tag{S18}$$

The corresponding surface dispersion is

$$E = \pm\sqrt{k_\parallel^2 + \lambda_y^2\sin^2 k_y + (m_2\cos\phi - m_1\sin\phi)^2}. \tag{S19}$$

Gapless hinge states appear when the surface mass vanishes, i.e., when the last term in Eq. (S19) vanishes. This yields the condition

$$m_2\cos\phi - m_1\sin\phi = 0 \tag{S20}$$

which fixes the angle ϕ at which the hinge lines are located on the cylinder. When this condition is satisfied, the surface becomes gapless along specific directions, and the hinge-state dispersion reduces to

$$E = \pm\sqrt{k_\parallel^2 + \lambda_y^2\sin^2 k_y}, \tag{S21}$$

in agreement with the numerical spectrum in Fig. S10(a).

S3.1.3. Effective Surface Hamiltonian: Two Copies of a Helical Dirac Cone

The surface theory may be viewed as a pair of helical Dirac cones subject to a mass term that changes sign across certain directions, leading to hinge-localized zero modes. To make the helical nature of the surface explicit, we set $m_2 \cos\phi - m_1 \sin\phi = 0$ and choose, for example, $\phi = \pi$, so that $k_\parallel \equiv k_x$. Expanding near $k_y = 0$, the surface Hamiltonian in Eq. (S18) reduces to the Dirac Hamiltonian

$$H_D(k_x, k_y) = k_x \tau_y + \lambda_y k_y \tau_z \sigma_z. \tag{S22}$$

After a suitable unitary transformation U, this surface Hamiltonian can be brought into the form

$$H'_D = U H_D U^\dagger = \tau_z \left(k_x \sigma_y - \lambda_y k_y \sigma_x \right), \tag{S23}$$

where

$$U = \frac{1}{2} \begin{pmatrix} -1 & 1 & -1 & -1 \\ 1 & 1 & -1 & 1 \\ -1 & -1 & -1 & 1 \\ -1 & 1 & 1 & 1 \end{pmatrix}.$$

In this representation the helical spin-momentum locking of the surface states is manifest. These helical modes form the essential ingredient for generating effective p-wave pairing on the surface via proximity to a conventional s-wave superconductor [34, 35], as discussed below.

S3.2. Proximity-Induced Surface s+p-wave Topological Superconductor

In the 1T′ phase, the coexistence of a bulk s-wave superconducting band and higher-order topological (SOTI) bands provides a natural setting for unconventional proximity effects. In particular, the helical Dirac surface states and hinge channels of the SOTI [Sec. S3.1] play the same role as the surface of a three-dimensional topological insulator, where it is well established that p-wave correlations can be induced when an s-wave superconductor is proximitized to the surface states [34–37]. In our case, single-particle tunneling between the bulk s-wave band and the SOTI band implements an intrinsic bulk-to-surface self-proximity effect. The bulk condensate induces both s- and p-wave components in the effective surface pairing. In this subsection, we formulate a minimal BdG model for this mechanism and analytically derive the induced pairing channels via a Schrieffer-Wolff transformation.

S3.2.1. Model System: Bulk s-wave Band Coupled to SOTI Bands

To describe superconductivity in the 1T′ phase, we consider a minimal model in which a conventional s-wave SC band hybridizes with the SOTI bands. The full BdG Hamiltonian is written as

$$H_{\text{Tot}}(\mathbf{k}) = \begin{pmatrix} H_{\text{SC}}(\mathbf{k}) & H_{\text{I}} \\ H_{\text{I}}^{\dagger} & H_{\text{SOTI}}^{\text{BdG}}(\mathbf{k}) \end{pmatrix}. \tag{S24}$$

Here, $H_{\text{SC}}(\mathbf{k})$ describes a low-energy s-wave superconductor near the Γ point,

$$H_{\text{SC}}(\mathbf{k}) = \left(-k_y^2 + \varepsilon_0\right)\xi_z + \Delta_{s\text{-wave}}\xi_x\tau_x\mu_z\sigma_y,$$

where $\Delta_{s\text{-wave}}$ is the intrinsic s-wave pairing amplitude, and ξ_i are Pauli matrices in Nambu space. The SOTI contribution $H_{\text{SOTI}}^{\text{BdG}}(\mathbf{k})$ is obtained by writing Eq. (S17) in Nambu form and expanding near the Γ point,

$$H_{\text{SOTI}}^{\text{BdG}}(\mathbf{k}) = -\mu\xi_z + M\xi_z\tau_z + k_x\tau_y\mu_y + k_z\tau_x + \lambda_y k_y\xi_z\tau_y\mu_z\sigma_z + m\left(\xi_z\tau_z\mu_z + \xi_z\mu_x\right),$$

where $M = m_0 + t_x + t_y + t_z$ is an effective Dirac mass, and for simplicity we take $M = 1$, $\lambda_x = \lambda_z = 1$, and $m_1 = m_2 \equiv m$. The matrices ξ_i, τ_j, μ_k, and σ_l act on Nambu, orbital, orbital, and spin sectors, respectively. The coupling between the SC and SOTI sectors is described by H_{I}, which we choose as

$$H_{\text{I}} = \sum_{j,s}\lambda\left(a_{j,s}^{\dagger}c_{j,s} + c_{j,s}^{\dagger}a_{j,s}\right), \tag{S25}$$

where $a_{j,s}$ and $c_{j,s}$ are annihilation operators for electrons with spin s on orbital j in the s-wave and SOTI sectors, respectively, and λ is the hybridization strength, treated as a small perturbative parameter.

S3.2.2. Schrieffer-Wolff Transformation and Induced Bulk Pairings

To obtain the effective SC pairing generated in the SOTI sector by proximity to the s-wave band, we treat $H_{\rm I}$ as a perturbation and perform a Schrieffer-Wolff (SW) transformation [38]. We decompose $H_{\rm Tot}(\mathbf{k})$ into an unperturbed part $H_0(\mathbf{k})$ and a perturbation V,

$$H_{\rm Tot}(\mathbf{k}) = H_0(\mathbf{k}) + V = \begin{pmatrix} H_{\rm SC}(\mathbf{k}) & 0 \\ 0 & H^{\rm BdG}_{\rm SOTI}(\mathbf{k}) \end{pmatrix} + \begin{pmatrix} 0 & H_{\rm I} \\ H_{\rm I}^\dagger & 0 \end{pmatrix}.$$

The SW method seeks a unitary transformation $U = e^S$ such that $V + [S, H_0(\mathbf{k})] = 0$, where S is anti-Hermitian and of the same order in λ as V. To the leading order of λ, the transformed Hamiltonian

$$H'_{\rm Tot}(\mathbf{k}) = U H_{\rm Tot}(\mathbf{k}) U^\dagger \approx H_0(\mathbf{k}) + \frac{1}{2}[S, V] = \begin{pmatrix} H'_{\rm SC}(\mathbf{k}) & 0 \\ 0 & H^{\rm BdG'}_{\rm SOTI}(\mathbf{k}) \end{pmatrix},$$

is block-diagonal in the SC and SOTI sectors, where $H'_{\rm SC}$ and $H^{\rm BdG'}_{\rm SOTI}$ are dressed Hamiltonians. Writing

$$S = \begin{pmatrix} A & B \\ C & D \end{pmatrix},$$

with blocks A, B, C, D of the same size as $H_{\rm SC}$, $H_{\rm I}$, $H_{\rm I}^\dagger$, and $H^{\rm BdG}_{\rm SOTI}$, the condition $V + [S, H_0] = 0$ leads to

$$V + \begin{pmatrix} A & B \\ C & D \end{pmatrix} H_0(\mathbf{k}) - H_0(\mathbf{k}) \begin{pmatrix} A & B \\ C & D \end{pmatrix} = 0.$$

This simplifies into

$$\begin{pmatrix} [A, H_{\rm SC}(\mathbf{k})] & B H^{\rm BdG}_{\rm SOTI}(\mathbf{k}) - H_{\rm SC}(\mathbf{k}) B + H_{\rm I} \\ C H_{\rm SC}(\mathbf{k}) - H^{\rm BdG}_{\rm SOTI}(\mathbf{k}) C + H_{\rm I}^\dagger & [D, H^{\rm BdG}_{\rm SOTI}(\mathbf{k})] \end{pmatrix} = 0,$$

which determines B and C while constraining A and D. The transformed Hamiltonian can then be written as

$$H'_{\rm tot}(\mathbf{k}) \approx H_0(\mathbf{k}) + \frac{1}{2} \begin{pmatrix} B H_{\rm I}^\dagger - H_{\rm I} C & A H_{\rm I} - H_{\rm I} D \\ D H_{\rm I}^\dagger - H_{\rm I}^\dagger A & C H_{\rm I} - H_{\rm I}^\dagger B \end{pmatrix} = \begin{pmatrix} H'_{\rm SC}(\mathbf{k}) & 0 \\ 0 & H^{\rm BdG'}_{\rm SOTI}(\mathbf{k}) \end{pmatrix}. \quad \text{(S26)}$$

Requiring the off-diagonal blocks of Eq. (S26) to vanish provides additional constraints that fully determine S. In practice, solving these equations yields an effective SOTI BdG Hamiltonian

$$H^{\rm BdG'}_{\rm SOTI}(\mathbf{k}) = \tilde{H}^{\rm BdG}_{\rm SOTI}(\mathbf{k}) + H^{\rm BdG}_s + H^{\rm BdG}_p(\mathbf{k}),$$

where $\tilde{H}_{\text{SOTI}}^{\text{BdG}}$ is the SOTI BdG Hamiltonian dressed by normal-state corrections, and H_s^{BdG} and H_p^{BdG} provide the proximity-induced s-wave and p-wave pairings, respectively. To the leading order of λ, we obtain two s-wave and three p-wave pairing gaps in the bulk SOTI sector,

$$\begin{aligned} H_s^{\text{BdG}} &= \Delta_{s1}\xi_x\left(\tau_x+\tau_y\mu_y\right)\sigma_y-\Delta_{s2}\xi_x\tau_x\mu_z\sigma_y \\ H_p^{\text{BdG}}(\mathbf{k}) &= -\Delta_{p1}(k_x-k_z)\xi_y\mu_y\sigma_y+k_y\xi_x\left[\Delta_{p2}\tau_0-\Delta_{p3}\left(\tau_z+\mu_x\right)\right]\sigma_x, \end{aligned}$$

with effective amplitudes

$$\begin{aligned} \Delta_{s1} &= \frac{2m\Delta\lambda^2\left[1+4m^4+2\mu^2-3\mu^4+4m^2\left(-1+\mu^2\right)\right]}{\left[4m^4+(-1+\mu^2)^2-4m^2\left(-1+\mu^2\right)\right]^2} \\ \Delta_{s2} &= \frac{\Delta\lambda^2\left[8m^6-4m^4\left(1+\mu^2\right)+\left(-1+\mu^2\right)^2\left(1+\mu^2\right)-2m^2\left(1-10\mu^2+\mu^4\right)\right]}{\left[4m^4+(-1+\mu^2)^2-4m^2\left(-1+\mu^2\right)\right]^2} \\ \Delta_{p1} &= \frac{2m\Delta\lambda^2\left[1+4m^4+2\mu^2-3\mu^4+4m^2\left(-1+\mu^2\right)\right]}{\left[4m^4+(-1+\mu^2)^2-4m^2\left(-1+\mu^2\right)\right]^2} \\ \Delta_{p2} &= \frac{2\Delta\lambda^2\lambda_y\mu\left[4m^4-4m^2\left(-3+\mu^2\right)+\left(-1+\mu^2\right)^2\right]}{\left[4m^4+(-1+\mu^2)^2-4m^2\left(-1+\mu^2\right)\right]^2} \\ \Delta_{p3} &= \frac{4\Delta\lambda^2\lambda_y m\mu\left(1+2m^2-\mu^2\right)}{\left[4m^4+(-1+\mu^2)^2-4m^2\left(-1+\mu^2\right)\right]^2}, \end{aligned}$$

where we have set $\Delta\equiv\Delta_{s\text{-wave}}$. Here we retain only the leading terms of Δ in the weak-coupling limit $\Delta\ll 1$. The corresponding fermion bilinears are listed in Table I of the main text, and form the basis of the effective s+p-wave pairing used in our phenomenology. Importantly, this derivation shows that the proximity effect does not merely induce a trivial on-site s-wave gap on the SOTI bands; instead, it naturally generates a hierarchy of s- and p-wave channels whose relative strengths are controlled by the microscopic parameters m, μ, λ and λ_y.

S3.2.3. Effective Induced BdG Hamiltonian and Surface $s+p$-wave Pairing

In what follows, we focus on the effective superconducting SOTI obtained by combining the BdG-expanded SOTI Hamiltonian with the induced pairing terms H_s^{BdG} and H_p^{BdG}. Normal-state corrections generated by the SW transformation are subleading and do not modify the bulk topological index; for clarity we neglect them here. The resulting induced BdG Hamiltonian for the 1T′ phase can be written as

$$\begin{aligned} H^{\mathrm{BdG}}(\mathbf{k}) = &-\mu\,\xi_z + \left(m_0 + \sum_i t_i \cos k_i\right)\xi_z\tau_z + \sin k_x \tau_y\mu_y + \sin k_z\tau_x + \lambda_y \sin k_y\,\xi_z\tau_y\mu_z\sigma_z \\ &+ m\left(\xi_z\tau_z\mu_z + \xi_z\mu_x\right) + \Delta_{s1}\,\xi_x\left(\tau_x + \tau_y\mu_y\right)\sigma_y - \Delta_{s2}\,\xi_x\tau_x\mu_z\sigma_y \\ &- \Delta_{p1}(\sin k_x - \sin k_z)\,\xi_y\mu_y\sigma_y + \sin k_y\xi_x\left[\Delta_{p2}\tau_0 - \Delta_{p3}\left(\tau_z + \mu_x\right)\right]\sigma_x. \end{aligned} \tag{S27}$$

Projecting Eq. (S27) onto the cylindrical surface [Sec. S3.2.5] yields a surface BdG Hamiltonian $H_{\mathrm{surf}}^{\mathrm{BdG}}(\mathbf{k})$ in which two s-wave pairings (Δ_{s1}, Δ_{s2}) and one p-wave pairing (Δ_{p3}) survive, while the other two p-wave channels (Δ_{p1}, Δ_{p2}) vanish under the projection. Thus, the bulk s-wave superconductivity induces a combined $s+p$-wave pairing on the SOTI surface.

The calculated temperature and magnetic-field dependence of the induced s- and p-wave gaps reproduce the key features of the experimental PCAR spectra [Figs. 4(c,f)]: both components share a common critical temperature T_c, while the p-wave contribution is more fragile, being readily suppressed by inversion-symmetry breaking or by an applied magnetic field. This agreement supports our interpretation that the observed surface $s+p$-wave response in the 1T′ phase originates from a bulk-to-surface self-proximity effect in a higher-order topological band structure.

S3.2.4. Topological $\mathbb{Z}_8$ Index and Robustness of the TOTSC Phase

To characterize the bulk topology of the SC 1T′ phase, we evaluate the $\mathbb{Z}_8$ higher-order topological index κ defined in Eq. (1) of the main text [31, 39, 40]. It is expressed in terms of parity eigenvalues of occupied states at the time-reversal-invariant momenta (TRIM) as

$$\kappa = \frac{1}{4} \sum_{K \in \text{TRIM}} \left[N^{+}(K) - N^{-}(K) \right] \mod 8, \tag{S28}$$

where $N^{\pm}(K)$ counts the number of occupied states with even/odd inversion parity at each TRIM point K. We have computed these parity eigenvalues both for the full Hamiltonian $H_{\text{Tot}}(\mathbf{k})$ of Eq. (S24) and for the induced BdG Hamiltonian of Eq. (S27); the results are summarized in Figs. S11(a,b). In both cases we find $\kappa = 4$, which corresponds to a TOTSC phase. This equality confirms that integrating out the SC band via the SW transformation does not change the bulk topology, and that moderate variations of the pairing amplitudes do not drive the system out of the TOTSC phase.

We have further verified the robustness of the TOTSC phase against simplifications of the pairing structure. In the main text and in the analytical derivations below we often set $\Delta_{s1} = \Delta_{p1} = 0$ for clarity, retaining only Δ_{s2} and Δ_{p2}, Δ_{p3} as the dominant pairing channels. The parity analysis shows that the index remains $\kappa = 4$ under this truncation, confirming that the reduced s+p-wave pairing structure still lies in the same topological phase as the full induced BdG Hamiltonian. This justifies the use of the simplified model for analytical calculations while preserving the essential higher-order topology.

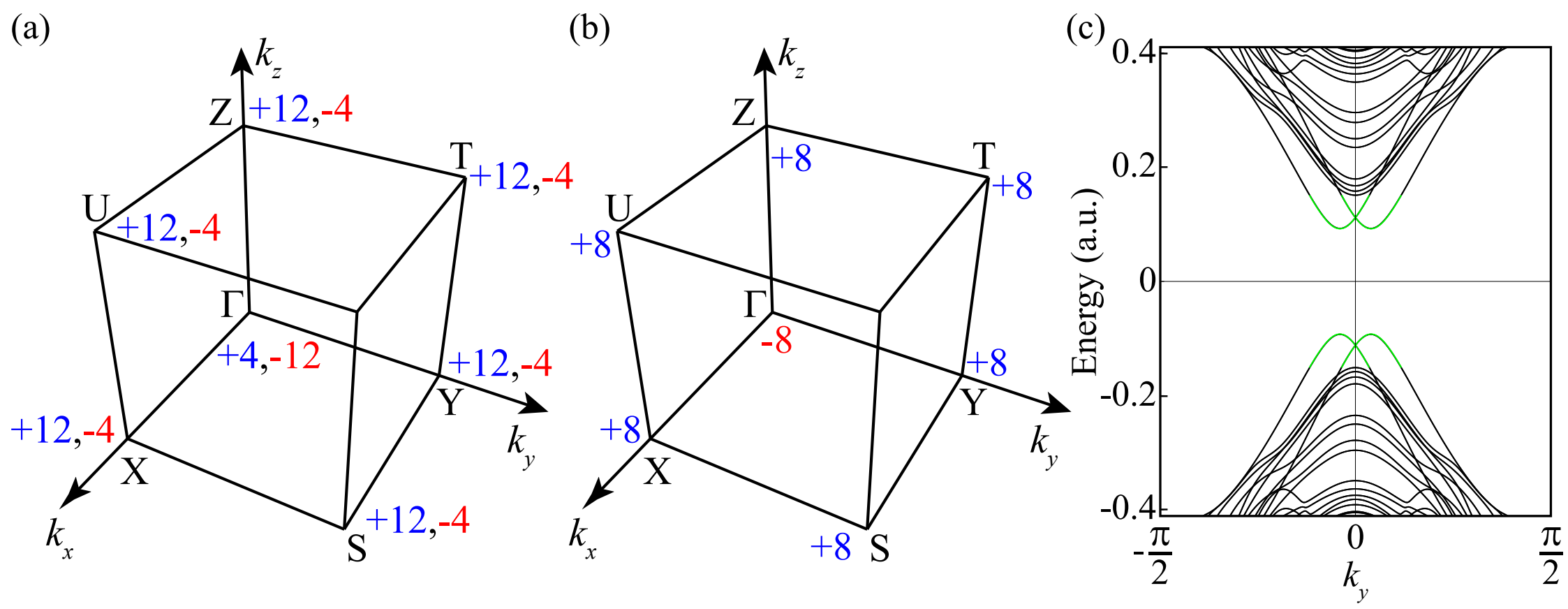

Fig. S11. **(a)** Parity eigenvalues of the occupied states at the TRIM points for the full Hamiltonian $H_{\text{Tot}}(\mathbf{k})$ in Eq. (S24). **(b)** Parity eigenvalues at the TRIM points for the induced BdG Hamiltonian $H^{\text{BdG}}(\mathbf{k})$ in Eq. (S27). In both cases the $\mathbb{Z}_8$ index is $\kappa = 4$, indicating a third-order topological SC phase. **(c)** Band structure of the finite SC SOTI system with 15×15 unit cells in the xz plane and periodic boundary conditions along y (black lines). The analytically derived hinge dispersion (green line) matches the numerical hinge bands near zero energy. Common parameters: $t_x = t_z = 1$, $t_y = 2$, $\lambda_x = \lambda_z = 1$, $\lambda_y = 0.5$, $m_0 = -3$, $m_1 = m_2 = 0.3$, and $\mu = 0.05$. For (a), we use $\lambda = 0.1$ and $\Delta_{s\text{-wave}} = 0.2$; for (b,c), $\Delta_{s1} = \Delta_{p1} = 0.03$, $\Delta_{s2} = 0.1$, $\Delta_{p2} = 0.05$, and $\Delta_{p3} = 0.04$.

S3.2.5. Hinge Hamiltonian and Majorana Physics in Cylindrical Geometry

We now derive the hinge Hamiltonian of the SC SOTI in a cylindrical geometry, following the strategy used for the normal-state SOTI. We impose periodic boundary conditions along y so that k_y remains a good quantum number, and treat k_x and k_z as differential operators normal and tangential to the surface when constructing the surface theory.

For clarity we set $\Delta_{s1} = \Delta_{p1} = 0$. As discussed above, this simplification does not affect the bulk topological index and only introduces small quantitative corrections to the surface gaps. The effect of reinstating these terms is addressed in Sec. S3.2.6.

Starting from Eq. (S27), the leading-order Dirac equation that governs surface-localized states near Γ is again $H_D = M(r)\xi_z\tau_z - i\partial_r(\cos\phi\,\tau_x + \sin\phi\,\tau_y\mu_y)$, with $M(r)$ a Dirac mass that changes sign across the surface. Using the same ansatz $|\psi_i\rangle = \mathcal{N}e^{-\int dr\,M(r)}|u_i\rangle$ for surface-localized solutions, we obtain the induced SC surface Hamiltonian

$$\begin{aligned} H^{\text{BdG}}_{\text{Surf}}(\mathbf{k}) \;=\; & -\mu\xi_z + k_\parallel\xi_z\sigma_y + m(\cos\phi - \sin\phi)\xi_z\left(\cos\phi\sigma_x + \sin\phi\sigma_z\right) \\ & -\lambda_y \sin k_y \tau_z\left(\cos\phi\sigma_z - \sin\phi\sigma_x\right) + \Delta_{s2}\xi_y\tau_y\left(\sin\phi\sigma_x - \cos\phi\sigma_z\right) \\ & +\Delta_{p3}\sin k_y\xi_x\tau_x\left(\sigma_0 - \sin^2\phi\sigma_x + \sin\phi\cos\phi\sigma_z\right), \end{aligned} \tag{S29}$$

which shows the interplay between the helical hinge channels and the induced s- and p-wave pairings.

We now solve for the hinge-localized states using the Jackiw-Rebbi method [41], treating $k_\parallel$ as $-i\partial_\parallel$ and viewing $m(\cos\phi - \sin\phi)$ as a mass term that changes sign across the hinge. At $\phi = \frac{\pi}{4}$, the resulting one-dimensional hinge BdG Hamiltonian is

$$H^{\text{BdG}}_{\text{Hinge}}(k_y) \;=\; -\mu\xi_z + \lambda_y\sin k_y\sigma_z + \Delta_{s2}\xi_y\sigma_y + \frac{\sqrt{2}+1}{\sqrt{2}}\Delta_{p3}\sin k_y\xi_x\sigma_x. \tag{S30}$$

The hinge dispersion then reads

$$E_{\text{Hinge}} = \pm\sqrt{\left(\lambda_y\sin k_y \pm \mu\right)^2 + \left(\Delta_{s2} \pm \frac{\sqrt{2}+1}{\sqrt{2}}\Delta_{p3}\sin k_y\right)^2}.$$

Crucially, the coexistence of the Δ_{s2} and Δ_{p3} terms in the hinge Hamiltonian indicate the effective s+p-wave character of the surface superconductivity that is probed in our PCAR measurements. We thus obtain a pair of gapped helical hinge modes, whose gap is controlled by a combination of the induced s-wave and momentum-dependent p-wave pairings.

S3.2.6. Majorana Corner States in Cylindrical Geometry

To obtain the explicit form of the Majorana corner states shown in Fig. 5 of the main text, we now solve the Jackiw-Rebbi problem for a finite hinge segment. For convenience we define $\Delta_s \equiv \Delta_{s2}$ and $\Delta_p \equiv \frac{\sqrt{2}+1}{\sqrt{2}}\Delta_{p3}$, so that Eq. (S30) becomes

$$\bar{H}^{\text{BdG}}_{\text{Hinge}}(k_y) \;=\; -\mu\xi_z + \lambda_y \sin k_y \sigma_z + \Delta_s \xi_y \sigma_y + \Delta_p \sin k_y \xi_x \sigma_x. \tag{S31}$$

We consider a hinge of finite length, $-\frac{L}{2} < y < \frac{L}{2}$, with corners at $y = \pm\frac{L}{2}$. In this geometry k_y is no longer a good quantum number, and the approximate $\sin k_y \simeq k_y$ is $-i\partial_y$ along the hinge. We take the pairing amplitudes Δ_s and Δ_p to be constants along the hinge that vanishes at the corners. Seeking zero-energy bound states of $\bar{H}^{\text{BdG}}_{\text{Hinge}}(-i\partial_y)$, we use an ansatz $|\psi_i\rangle = e^{\int dy\, X(y)}|u_i\rangle$ and obtain four solutions for $X(y)$,

$$X(y) = \pm\left[\frac{\Delta_s\lambda_y - \mu\Delta_p}{\lambda_y^2 + \Delta_p^2} \pm i\frac{\lambda_y\mu + \Delta_s\Delta_p}{\lambda_y^2 + \Delta_p^2}\right] y. \tag{S32}$$

Solutions with positive (negative) real part of $X(y)$ for the upper (lower) corner at $y = \frac{L}{2}$ ($y = -\frac{L}{2}$). Imposing particle-hole symmetry, we construct Jackiw-Rebbi-type solutions for the top and bottom corners,

$$\psi^{\pm}_T(y) = \exp\left[\frac{\Delta_s\lambda_y - \mu\Delta_p}{\lambda_y^2 + \Delta_p^2}\left(-y + \frac{L}{2}\right)\right]\begin{pmatrix} e^{i\theta_-} \\ \pm e^{-i\theta_+} \\ \pm e^{-i\theta_-} \\ e^{i\theta_+} \end{pmatrix}, \tag{S33}$$

$$\psi^{\pm}_B(y) = \exp\left[\frac{\Delta_s\lambda_y - \mu\Delta_p}{\lambda_y^2 + \Delta_p^2}\left(y + \frac{L}{2}\right)\right]\begin{pmatrix} e^{i\theta_+} \\ \pm e^{-i\theta_-} \\ \pm e^{-i\theta_+} \\ e^{i\theta_-} \end{pmatrix}, \tag{S34}$$

where $\theta_\pm = \frac{\lambda_y\mu + \Delta_s\Delta_p}{\lambda_y^2 + \Delta_p^2} y \pm \frac{\pi}{4}$. The spinors $\psi^{\pm}_T$ and $\psi^{\pm}_B$ form Kramers' pairs of Majorana corner states at the top and bottom ends of the hinge [42, 43]. They obey particle-hole symmetry $C\psi^{\pm}_{T,B} = \pm\psi^{\pm}_{T,B}$ and time-reversal symmetry $\Theta\psi^{\pm}_{T,B} = \mp i\psi^{\mp}_{T,B}$, with $C = \xi_x K$ and $\Theta = -i\sigma_y K$. By inspection, $\left[\psi^{\pm}_{T,B}(y)\right]^C = \pm\psi^{\pm}_{T,B}(y)$, confirming their Majorana nature. Therefore, the finite hinge in the cylindrical geometry hosts a pair of time-reversal-related Majorana modes at each end, realizing the characteristic boundary signature of a TOTSC. These analytical solutions reproduce the numerically obtained corner-localized modes shown in the main text.

S3.2.7. Effect of Additional Pairing Terms in Cylindrical Geometry

In Sec. S3.2.4 and in the main text we set $\Delta_{s1} = \Delta_{p1} = 0$ for simplicity. Here we discuss the impact of restoring these terms. As before, we find the Dirac equation to obtain the localized states at the surface from the induced BdG Hamiltonian of Eq. (S27) in the presence of Δ_{s1} and Δ_{p1} terms. In this case, the Dirac equation near the Γ point is given as $H_D = M(r)\xi_z\tau_z - i\partial_r(\cos\phi\tau_x + \sin\phi\tau_y\mu_y) - \Delta_{p1}(\sin k_x - \sin k_z)\xi_y\mu_y\sigma_y$ in the cylindrical geometry.

The trial wavefunctions for the surface localized wavefunctions have the similar form of $|\psi_i^\pm\rangle = \mathcal{N}\exp\left[-\int dr M^\pm(r)\right]|u_i^\pm\rangle$ and satisfies the equation of $H_D|\psi_i^\pm\rangle = 0$. However, due to the additional Δ_{p1} term, there is a slight change of the solutions for the surface-localized wavefunctions:

$$M^\pm(r) = M(r) \pm \epsilon + O(\epsilon^2),$$

where ϵ is a small constant proportional to Δ_{p1}. Using these surface wavefunctions, the resulting surface Hamiltonian to the leading order of ϵ is given by

$$\begin{aligned}
\tilde{H}^{\text{BdG}}_{\text{Surf}}(\mathbf{k}) \;=\; & -\mu\xi'_z - k_\parallel\xi'_z\tau_y - m(\cos\phi - \sin\phi)\left(\cos\phi\tau'_x - \sin\phi\tau'_z\right) \\
& +\lambda_y \sin k_y\left(\cos\phi\tau'_z + \sin\phi\tau'_x\right)\sigma'_z + \Delta_{s2}\xi'_x\left(\cos\phi\tau'_x - \sin\phi\tau'_z\right) \\
& +\Delta_{p3}\sin k_y\xi'_y\tau'_y\sigma'_z + \Delta_{p3}\sin k_y\sin\phi\xi'_x\left(\sin\phi\tau'_z - \cos\phi\tau'_x\right)\sigma_z \\
& +\Delta_{s1}(\sin\phi + \cos\phi)\xi'_y\tau'_y \\
& +2\epsilon\left[m(\cos\phi - \sin\phi)\xi'_x\left(\sin\phi\tau'_z - \cos\phi\tau'_x\right) + \lambda_y\sin k_y\xi'_x\left(\sin\phi\tau'_x + \cos\phi\tau'_z\right)\sigma'_z\right].
\end{aligned} \tag{S35}$$

Here, the Pauli matrices ξ'_i, τ'_j and σ'_k represent another basis and hence do not indicate the previous physical degree of freedom and differ from ξ_i, τ_j and σ_k. However, under a unitary transformation, Eq. (S35) transforms into the same form with the previous surface Hamiltonian in Eq. (S29) except for the new terms proportional to Δ_{s1} and 2ϵ in the last two lines of Eq. (S35).

With this new surface Hamiltonian, we analytically calculate the hinge Hamiltonian and its dispersion.

$$\tilde{E}_{\text{Hinge}} = \pm\sqrt{(\lambda_y\sin k_y \pm \mu)^2 + \left(\Delta_{s2} - \sqrt{2}\Delta_{s1} \pm \frac{\sqrt{2}+1}{\sqrt{2}}\Delta_{p3}\sin k_y\right)^2}.$$

Moreover, we have obtained the Majorana corner states using the hinge Hamiltonian.

Under such transformation, these terms act as additional pairing terms for the surface Hamiltonian and therefore there is a difference between the analytical value of the hinge and the TB results when $\Delta_{p1} \neq 0$. The difference is small and can be seen in Fig. S11(c). Thus, the inclusion of Δ_{s1} and Δ_{p1} only leads to quantitative renormalizations of the hinge gap and the spatial profile of the modes, leaving the qualitative existence and topology of the Majorana corner states unchanged.

S3.2.8. Majorana Corner States in Spherical Geometry

Finally, we generalize the above analysis to a spherical geometry, which is particularly convenient for visualizing higher-order topology. We again construct a surface Dirac theory and derive the hinge and corner states on a sphere. As in the cylindrical case, we set $\Delta_{s1} = \Delta_{p1} = 0$ for simplicity; including them only leads to small quantitative changes and does not modify the topology.

Using spherical coordinates, we write (k_x, k_y, k_z) in terms of (k_r, k_θ, k_ϕ), where k_r is radial and k_θ, k_ϕ are tangential momenta. On a spherical surface, k_θ and k_ϕ are good quantum numbers, while k_r becomes $-i\partial_r$. We choose the polar angle θ from the y axis and the azimuthal angle ϕ from the z axis, consistent with the main-text convention.

Linearizing Eq. (S27) near Γ and taking k_r as $-i\partial_r$ leads to a surface Dirac equation similar in structure to the cylindrical case but expressed in spherical coordinates. When taking $\lambda_x = \lambda_y = \lambda_z = 1$ for simplicity, the resulting Dirac equation of Eq. (S27) near the Γ point is given as

$$H_D = M(r)\xi_z\tau_z - i\partial_r(\sin\theta\cos\phi\tau_x + \sin\theta\sin\phi\tau_y\mu_y + \cos\theta\xi_z\tau_y\mu_z\sigma_z).$$

Projecting onto the surface-localized states yields a spherical surface Hamiltonian, from which we can again derive hinge-like one-dimensional structures and their corner-localized Majorana modes:

$$\begin{aligned} H_{\text{Surf}}^{\text{BdG}'}(\mathbf{k}) &= -\mu\xi_z + \tau_z\left[(k_\theta\cos\phi - k_\phi\cos\theta\sin\phi)\,\sigma_z - (k_\theta\sin\phi + k_\phi\cos\theta\cos\phi)\,\sigma_x\right] + k_\phi\sin\theta\xi_z\sigma_y \\ &\quad + m\sin\theta(\cos\phi - \sin\phi)\left[\sin\theta\xi_z\left(\cos\phi\sigma_x + \sin\phi\sigma_z\right) + \cos\theta\sigma_y\right] \\ &\quad + \Delta_{s2}\sin\theta\xi_y\tau_y\left(\sin\phi\sigma_x - \cos\phi\sigma_z\right) + \Delta_{p3}\sin^2\theta\cos\theta\cos\phi\xi_y\tau_y\sigma_y \\ &\quad + \Delta_{p3}k_\theta\sin\theta\xi_x\tau_x\left[\sigma_0 - \left(1 - \sin^2\theta\cos^2\phi\right)\sin^2\phi\sigma_x + \sin^2\theta\sin\phi\cos\phi\sigma_z\right], \end{aligned} \tag{S36}$$

and we use this Hamiltonian to find the dispersion of the hinges.

The hinge states and their energy-momentum dispersion relation can be obtained by solving the surface Hamiltonian using the Jackiw-Rebbi method [41] after a transformation of k_ϕ to $-i\partial_\phi$. Due to the nature of the polar and azimuthal angle regarding the spherical coordinate being $0 \leq \theta \leq \pi$ and $0 \leq \phi \leq 2\pi$, respectively, we first need to solve the hinge as two different curves along $\phi = \frac{\pi}{4}$ and $\phi = \frac{5\pi}{4}$, where the mass inversion occurs due to the second line of Eq. (S36). We then combine the two curves and treat the hinge Hamiltonian as a 1-dimensional circle along a polar coordinate with a polar angle $-\pi \leq \theta \leq \pi$. Solving the Jackiw-Rebbi differential equation along the surface with $m(\cos\phi - \sin\phi)$ being the mass term,

the hinge dispersions at $\frac{\pi}{4}$ and $\frac{5\pi}{4}$ are given by

$$
\begin{aligned}
H_{\frac{\pi}{4}}^{\mathrm{BdG}'}(k_\theta) &= -\mu\xi_z + (k_\theta + i\eta(\theta)\cos\theta)\,\sigma_z + \Delta_{s2}\sin\theta\xi_y\sigma_y \\
&\quad + \frac{\sqrt{2}+1}{\sqrt{2}}\Delta_{p3}\sin\theta\,(k_\theta + i\eta(\theta)\cos\theta)\,\xi_x\sigma_x, \qquad \text{(S37)}\\
H_{\frac{5\pi}{4}}^{\mathrm{BdG}'}(k_\theta) &= -\mu\xi_z + (k_\theta + i\eta(\theta)\cos\theta)\,\sigma_z - \Delta_{s2}\sin\theta\xi_y\sigma_y \\
&\quad - \frac{\sqrt{2}+1}{\sqrt{2}}\Delta_{p3}\sin\theta\,(k_\theta + i\eta(\theta)\cos\theta)\,\xi_x\sigma_x, \qquad \text{(S38)}
\end{aligned}
$$

where $\eta(\theta)$ is

$$
\eta(\theta) = m\left(\frac{e^{\sqrt{2}m\sin\theta} m\sin\theta\,{}_0\tilde{F}_1\left(;2;2m^2\sin^2\theta\right)\sqrt{\frac{2}{\pi}}}{{}_0\tilde{F}_1\left(;1;2m^2\sin^2\theta\right)^{3/2}} + \frac{e^{\sqrt{2}m\sin\theta}}{\sqrt{\pi\,{}_0\tilde{F}_1\left(;1;2m^2\sin^2\theta\right)}}\right),
$$

with ${}_0\tilde{F}_1\left(;x;z\right)$ being the regularized confluent hypergeometric function ${}_0F_1\left(;x;z\right)/\Gamma(x)$. Combining the two Hamiltonians and considering the hinge as a 1-dimensional circle with polar angles $-\pi \le \theta \le \pi$ and setting $\Delta_s \equiv \Delta_{s2}$, and $\Delta_p \equiv \frac{\sqrt{2}+1}{\sqrt{2}}\Delta_{p3}$, the resulting Dirac equation derived from the hinge Hamiltonian is

$$
\begin{aligned}
\bar{H}_{\mathrm{Hinge}}^{\mathrm{BdG}'}(\theta) &= \begin{cases} H_{\frac{5\pi}{4}}^{\mathrm{BdG}'}(\theta), & -\pi \le \theta \le 0 \\ H_{\frac{\pi}{4}}^{\mathrm{BdG}'}(\theta), & 0 \le \theta \le \pi \end{cases} \qquad \text{(S39)}\\
&= -\mu\xi_z - i\left[\partial_\theta - \eta(\theta)\cos\theta\right]\sigma_z + \Delta_s\sin\theta\xi_y\sigma_y - i\Delta_p\sin\theta\left[\partial_\theta - \eta(\theta)\cos\theta\right]\xi_x\sigma_x.
\end{aligned}
$$

Using the same methodology used to find the Jackiw-Rebbi solution of the Dirac equation derived via Eq. (S31), the ansatz of $|\psi_i\rangle = \exp\left[\int d\theta X(\theta)\right]|u_i\rangle$ being the localized states on the corner is used. Plugging this ansatz into the zero-energy eigenvalue equation $\bar{H}_{\mathrm{Hinge}}^{\mathrm{BdG}'}(-i\partial_y)|\psi_i\rangle = 0$, we find the four following solutions for $X(\theta)$,

$$
X(\theta) = \eta(\theta)\cos\theta \pm \left[\frac{\Delta_s\sin\theta - \mu\Delta_p\sin\theta}{1+\Delta_p^2\sin^2\theta} \pm i\frac{\mu + \Delta_s\Delta_p\sin^2\theta}{1+\Delta_p^2\sin^2\theta}\right],
$$

The phase is equal to Eq. (S32) when $\theta = \pi/2$ and $\lambda_y = 1$. Considering the convergence of the localized states, the corner state at the north pole is given by the $X(\theta)$ terms with the positive real part, while the corner state at the south pole given by the $X(\theta)$ terms with a negative real part, respectively. As the solution should satisfy the particle-hole symmetry, a linear combination $|\psi_i\rangle$ with different $X(\theta)$'s will be a Jackiw-Rebbi solution. The resulting Jackiw-Rebbi solutions give the wavefunctions for the top $\psi_N^{\pm}(\theta)$ and

wavefunctions for the bottom $\psi_S^{\pm}(\theta)$ as

$$\psi_N^{\pm}(\theta) = e^{\int d\theta\left[\frac{\Delta_s \sin\theta - \mu\Delta_p \sin\theta}{1+\Delta_p^2 \sin^2\theta} + \eta(\theta)\cos\theta\right]} \begin{pmatrix} e^{i\alpha_-} \\ \pm e^{-i\alpha_+} \\ \pm e^{-i\alpha_-} \\ e^{i\alpha_+} \end{pmatrix}, \tag{S40}$$

$$\psi_S^{\pm}(\theta) = e^{\int d\theta\left[-\frac{\Delta_s \sin\theta - \mu\Delta_p \sin\theta}{1+\Delta_p^2 \sin^2\theta} + \eta(\theta)\cos\theta\right]} \begin{pmatrix} e^{i\alpha_+} \\ \pm e^{-i\alpha_-} \\ \pm e^{-i\alpha_+} \\ e^{i\alpha_-} \end{pmatrix}, \tag{S41}$$

where $\alpha_{\pm} = \pm\frac{\pi}{4} + \int d\theta \left[\frac{\mu + \Delta_s\Delta_p \sin^2\theta}{1+\Delta_p^2 \sin^2\theta}\right]$. The wavefunctions $\psi_N^{\pm}(\theta)$ and $\psi_S^{\pm}(\theta)$ are Majorana Kramers' pairs, respectively [42, 43]. It should also be noted that these Majorana Kramers' pairs cannot create either a wavefunction of pure particle or hole, which is clear when we consider the form of the wavefunctions. The wavefunctions each satisfy the particle-hole symmetry $C\psi_{N,S}^{\pm} = \pm\psi_{N,S}^{\pm}$ for the charge-conjugation operator $C = \xi_x K$, where K is a conjugation operator. The wavefunctions satisfy the particle-hole symmetry $C\psi_{N,S}^{\pm} = \pm\psi_{N,S}^{\pm}$ and time-reversal symmetry $\Theta\psi_{N,S}^{\pm} = \mp i\psi_{N,S}^{\mp}$ for the charge-conjugation and time-reversal operators ($C = \xi_x K$ and $\Theta = -i\sigma_y K$ with K being a conjugation operator). When examining the wavefunctions, it can be seen that $\left[\psi_{N,S}^{\pm}(\theta)\right]^C = \pm\psi_{N,S}^{\pm}(\theta)$, indicating that the wavefunctions are indeed a Majorana Kramers' pair. In the spherical geometry, these solutions describe pairs of Majorana modes localized at the north and south poles, providing a complementary and fully three-dimensional picture of the same TOTSC phase that appears as hinge-terminated corner modes in the cylindrical geometry. The wavefunctions for the respective Majorana corners states agree with the findings given in the main text.

[1] Daghero, D. & Gonnelli, R. Probing multiband superconductivity by point-contact spectroscopy. Superconductor Science and Technology **22**, 043001 (2010).

[2] Daghero, D., Tortello, M., Ummarino, G. & Gonnelli, R. Directional point-contact andreev-reflection spectroscopy of fe-based superconductors: Fermi surface topology, gap symmetry, and electron–boson interaction. Reports on Progress in Physics **74**, 124509 (2011).

[3] Werthamer, N., Helfand, E. & Hohenberg, P. Temperature and Purity Dependence of the Superconducting Critical Field, H_{c2}. III. Electron Spin and Spin-Orbit Effects. Physical Review B **147**, 295 (1966).

[4] Bhoi, D. et al. Interplay of charge density wave and multiband superconductivity in 2*H-*Pd_xTaSe_2. Scientific Reports **6**, 24068 (2016).

[5] Gurevich, A. Enhancement of the upper critical field by nonmagnetic impurities in dirty two-gap superconductors. Physical Review B **67**, 184515 (2003).

[6] Ledvij, M. & Dovrosavljević-Grujić, L. Positive curvature in perpendicular critical field of high-temperature superconductors. Modern Physics Letters B **4**, 471 (1990).

[7] Guguchia, Z. et al. Signatures of the topological s^{+-} superconducting order parameter in the type-II Weyl semimetal T_d-$MoTe_2$. Nature Communications **8**, 1082 (2017).

[8] Naidyuk, Y. et al. Surface superconductivity in the Weyl semimetal $MoTe_2$ detected by point contact spectroscopy. 2D Materials **5**, 045014 (2018).

[9] Szabó, P. et al. Evidence for two-gap superconductivity in $Ba_{0.55}$ $K_{0.45}$ Fe_2 As_2 from directional point-contact Andreev-reflection spectroscopy. Physical Review B **79**, 012503 (2009).

[10] Daghero, D., Tortello, M., Pecchio, P., Stepanov, V. & Gonnelli, R. Point-contact Andreev-reflection spectroscopy in anisotropic superconductors: the importance of directionality. Low Temperature Physics **39**, 199–210 (2013).

[11] Suhl, H., Matthias, B. & Walker, L. Bardeen-cooper-schrieffer theory of superconductivity in the case of overlapping bands. Physical Review Letter **3**, 552 (1959).

[12] Uemura, Y. J. Commonalities in phase and mode. Nature materials **8**, 253–255 (2009).

[13] Park, E. et al. Spectroscopic evidence for two-gap superconductivity in the quasi-1D chalcogenide $Nb_2Pd_{0.81}S_5$. J. Phys.: Condens. Matter **30**, 165401 (2018).

[14] Ding, S. et al. Surface structure and multi-gap superconductivity of V3Si (111) revealed by scanning tunneling microscopy. Quantum Frontier **2**, 3 (2023).

[15] Ding, S. et al. The energy gap of the compound $FeSe_{0.5}Te_{0.5}$ determined by specific heat and Point Contact Spectroscopy. Solid State Communications **220**, 21 (2015).

[16] Wilde, D. Y. et al. Scanning Tunneling Microscopy Observation of a Square Abrikosov Lattice in $LuNi_2B_2C$. Phys. Rev. Lett. **78**, 22 (1997).

[17] Zhang, X. et al. Evidence of a universal and isotropic 2 Δ/k BTC ratio in 122-type iron pnictide superconductors over a wide doping range. Physical Review B **82**, 020515(R) (2010).

[18] Sheet, G., Mukhopadhyay, S. & Raychaudhuri, P. Role of critical current on the point-contact andreev reflection spectra between a normal metal and a superconductor. Physical Review B **69**, 134507 (2004).

[19] Wang, H., Ma, L. & Wang, J. Tip-induced or enhanced superconductivity: a way to detect topological superconductivity. Science Bulletin **63**, 1141–1150 (2018). URL `https://www.sciencedirect.com/science/article/pii/S2095927318303712`.

[20] Manna, S. et al. Signature of a pair of majorana zero modes in superconducting gold surface states. Proceedings of the National Academy of Sciences **117**, 8775–8782 (2020).

[21] Chen, J. et al. Quantum effects and phase tuning in epitaxial hexagonal and monoclinic $MoTe_2$ monolayers. ACS Nano **11**, 3282–3288 (2017).

[22] Wang, H. et al. Observation of superconductivity induced by a point contact on 3D Dirac semimetal Cd_3As_2 crystals. Nature Materials **15**, 38–42 (2016).

[23] Aggarwal, L. et al. Unconventional superconductivity at mesoscopic point contacts on the 3D Dirac semimetal Cd_3As_2. Nature Materials **15**, 32–37 (2016).

[24] Huang, F.-T. et al. Polar and phase domain walls with conducting interfacial states in a weyl semimetal mote2. Nature communications **10**, 4211 (2019).

[25] Singh, S., Kim, J., Rabe, K. M. & Vanderbilt, D. Engineering Weyl phases and nonlinear Hall effects in T_d-$MoTe_2$. Physical review letters **125**, 046402 (2020).

[26] Ezawa, M. Second-order topological insulators and loop-nodal semimetals in transition metal dichalcogenides XTe_2 (X= Mo, W). Scientific Reports **9**, 5286 (2019).

[27] Wang, Z., Wieder, B. J., Li, J., Yan, B. & Bernevig, B. A. Higher-Order Topology, Monopole Nodal Lines, and the Origin of Large Fermi Arcs in Transition Metal Dichalcogenides XTe_2 (X= Mo, W). Physical Review Letters **123**, 186401 (2019).

[28] Xu, N. et al. Evidence of a Coulomb-interaction-induced Lifshitz transition and robust hybrid Weyl semimetal in T_d-$MoTe_2$. Physical review letters **121**, 136401 (2018).

[29] Jiang, J. et al. Signature of type-II Weyl semimetal phase in $MoTe_2$. Nature communications **8**, 13973 (2017).

[30] Soluyanov, A. A. et al. Type-II Weyl Semimetals. Nature **527**, 495–498 (2015).

[31] Huang, S.-J., Park, K. & Hsu, Y.-T. Hybrid-order topological superconductivity in a topological metal 1T'-$MoTe_2$. npj Quantum Materials **9**, 21 (2024).

[32] Schindler, F. et al. Higher-order topological insulators. Science Advance **4**, eaat0346 (2018).

[33] Schindler, F. Dirac equation perspective on higher-order topological insulators. Journal of Applied Physics **128** (2020).

[34] Fu, L. & Kane, C. L. Superconducting proximity effect and Majorana fermions at the surface of a topological insulator. Physical Review Letters **100**, 096407 (2008).

[35] Stanescu, T. D., Sau, J. D., Lutchyn, R. M. & Sarma, S. D. Proximity effect at the superconductor–topological insulator interface. Physical Review B **81**, 241310 (2010).

[36] Zhang, P. et al. Observation of topological superconductivity on the surface of an iron-based superconductor. Science **360**, 182–186 (2018).

[37] Qin, W., Gao, J., Cui, P. & Zhang, Z. Two-dimensional superconductors with intrinsic p-wave pairing or nontrivial band topology. Science China Physics, Mechanics & Astronomy **66**, 267005 (2023).

[38] Schrieffer, J. R. & Wolff, P. A. Relation between the Anderson and Kondo Hamiltonians. Physical Review **149**, 491 (1966).

[39] Ahn, J. & Yang, B.-J. Higher-order topological superconductivity of spin-polarized fermions. Physical Review Research **2**, 012060 (2020).

[40] Khalaf, E., Po, H. C., Vishwanath, A. & Watanabe, H. Symmetry indicators and anomalous surface states of topological crystalline insulators. Physical Review X **8**, 031070 (2018).

[41] Jackiw, R. & Rebbi, C. Solitons with fermion number $1/2$. Physical Review D **13**, 3398 (1976).

[42] Haim, A. & Oreg, Y. Time-reversal-invariant topological superconductivity in one and two dimensions. Physics Reports **825**, 1–48 (2019).

[43] Zhang, F., Kane, C. & Mele, E. Time-reversal-invariant topological superconductivity and majorana kramers pairs. Physical review letters **111**, 056402 (2013).
