# Supplementary material for: Symmetry-controlled multi-gap superconductivity and higher-order topological phases of MoTe2
Source: Nat Commun. 2026 May 19;17:7685. doi: 10.1038/s41467-026-72368-x (PMC13434778; doi:10.1038/s41467-026-72368-x)
Supplement: Supplementary file 2 [file 41467_2026_72368_MOESM2_ESM.pdf]

nature portfolio

Peer Review File

Symmetry-Controlled Multi-gap Superconductivity and Higher-Order Topological Phases of MoTe$_2$

Corresponding Author: Professor Sangmo Cheon

This file contains all reviewer reports in order by version, followed by all author rebuttals in order by version.

Version 0:

Reviewer comments:

Reviewer #1

(Remarks to the Author)
Referee report

The manuscript "Evidence of surface p-wave superconductivity and higher-order topology in MoTe2“ by Lee et al. addresses a non conventional superconductivity in two topological phases controlled by the pressure in the transition metal dichalcogenide MoTe2. The subject is hot and important. The paper based on the point-contact Andreev reflection (PCAR) spectroscopy measurements at high pressures which is a demending technique concludes that the lower pressure Td phase is characterized by two superconducting energy gaps of s+- symmetry while the higher pressure 1T’ phase presents o contribution of p-wave surface superconductivity along with s-wave bulk component. The experiment of the soft point contact at high pressure is interesting and generally capable to shed a light on the superconducting parameter in such a non trivial system but the quality of the obtained data and their analysis do not provide a firm evidence on the claimed superconducting order parameters which is needed in a such high profile journal. Then, I do not recommend a publication of the paper. The issues are the following

• The double conductance peaks in the differential conductance observed at 0.7 GPa below Tc, as shown Fig. 2(a), is consistent with the single s-wave SC gap but also with two gaps with the gaps ratio 2Δ/kTc of 3.2 and 1.4. Obviously the more fit parameters the better fit but here a single gap is apparently satisfactory and the two gaps has unusual strength. In two gaps superconductors one gap is usually smaller and another one larger than the BCS limit.
• For the two-gap BTK fit all other parameters as Zi, Γi, weight of particular gap as well as some measure of quality of the two gap fit in comparison with the single-gap one are missing.
• Indeed, the shape of the spectra taken at 0.5 kG reminds a presence of two gaps (the same holds for Fig. S2(b) but again any quantitative analysis of the fit or even fit is missing (SI). As a paradox the experimental data taken at 0.5 kG seems to be more “two-gap like” than the fit but without the fit parameters is is difficult to make any conclusions.
• The field dependence of two gaps in Fig. 2© is generally not correctly obtained as the junction is obviously in the vortex state which is difficult to model.
• The temperature dependence of the two gaps given in the inset of Fig. 2© lacks any explanation how the intraband and interband couplings are accounted for in the fit.
• The temperature dependence of the upper critical magnetic field: Any details on experimental data are missing, how obtained, how defined. There is good fit by two gaps. Is there any distinction between s+- and s++?
• Even if we accept a two gap superconductivity in the Td phase, how can the authors distinguish their s+- and s++ version? They just recall Refs. 27 and 28 to support s+-…
• Fig. S1 in Suppl. Information claims temperature evolution but this is only the spectrum at 0.3 K with two undistinguishable fits.
• At higher pressure above 1.2 GPa (Fig. 3 (d)) a new type of spectra have been observed with the zero-bias conductance peak (ZBCP) which seems to be neither from a thermal effect nor an effect of magnetic impurity (no split under the field). But ZBCP is rapidly suppressed by a relatively small magnetic field of 1 kG (0.1 T). If this is due to a p-wave superconductivity it is strange.
• The authors analyse different gap symmetries in the Supplementary Information and compare the simulated spectra with the measured ones. The main conclusion is that the best fit is by a combination of the s-wave gap and the p-wave gap (Fig. 3(a), black not blue lines) but the details of this fit are missing. There is no trace of two gaps in the form of two pairs of peaks in thenraw data. What is the temperature dependence of these two gaps, do they have a common and what Tc? Etc.

Reviewer #2

(Remarks to the Author)
In this work, the authors studied the superconducting properties of the Td phase and 1T' phase of $MoTe_2$. Using soft-point contact Andreev reflection spectroscopy, the authors claimed that they observed s±-wave pairing in the Td phase. As pressure increases, the material undergoes a phase transition into the 1T' phase. In the 1T' phase, a zero-bias conductance peak was observed. Theoretical analysis further suggests that there are Majorana hinge modes in the 1T' superconducting phase.

In the experiment, the presence of two-gap superconductivity is convincing. However, there is no experimental evidence that the pairing is s±-wave. The observation of disorder-induced reduction of Tc in Td-$MoTe_2$ (as observed in previous works) is not strong enough to claim that $MoTe_2$ is in the s±-pairing state. When the charge carrier density is not high, it is reasonable to expect that Tc can be sensitive to changes in disorder strength. Based on the current experimental observations, the authors can only claim that Td-$MoTe_2$ is a two-gap superconductor but cannot claim that it is a s+- -wave superconductor.

The second major experimental finding of the authors is the appearance of the zero-bias conductance peak observed in the 1T' phase. The authors claim that the zero-bias conductance peak can be explained by s+p-wave pairing. I am not convinced by this claim. Zero-bias peaks can be observed in many multigap superconductors. In the fitting, I suspect that the authors used a small p-wave pairing gap and a large s-wave gap in the model calculation. In this case, the zero-bias peak emerges because of the smallness of the p-wave gap. In a multigap superconductor, a zero-bias peak can emerge without p-wave pairing. When one pairing gap from some Fermi pockets is much smaller than the other pairing gap from other Fermi pockets, a zero-bias peak can emerge. I am not convinced that the observed zero-bias conductance peak was induced by s+p-wave pairing.

Furthermore, the authors claimed that there are Majorana hinge modes in the material. There is no evidence for this claim. To claim the existence of hinge states, one probe the tunnelling conductance at the hinges. This kind of experiments are not performed by the authors.

The experimental observations of the authors are not entirely trivial. However, I am not convinced that there is evidence of p-wave superconductivity in $MoTe_2$. There is also no evidence of higher-order topology in the material. I do not recommend the publication of this work in Nature Communications.

Version 1:

Reviewer comments:

Reviewer #1

(Remarks to the Author)
The authors have addressed the issues raised in the initial reports and have made several changes to the manuscript, which certainly improve its quality. However, I do not believe the paper is ready for publication in Nature Communications.

Regarding the orthorhombic Td phase, the authors now provide more detailed parameters for their two-gap fit and have moved most of this section to the Supplementary Information. Nevertheless, some uncertainties remain. In the tables, does γ represent the weight of a specific gap? If so, is it associated with the smaller gap in both cases—s-wave in the Td phase (Tables S1 and S2) and s+p in the 1T' phase (Tables S3 and S4)? In Section S1.5, this weight is denoted as w—is this consistent?

In my previous report, I raised concerns about the unusual coupling strength when both gaps are below the BCS limit. This point has not yet been discussed, even though I am unaware of any other examples of such behavior.

I have acknowledged that the spectra in a magnetic field indicate the presence of two gaps, whereas the zero-field data do not. However, this discrepancy still requires discussion. For instance, a similar phenomenon was observed in MgB_2, where, under dominant c-axis tunneling, only a small gap on the π-band was detected in zero field. However, under a magnetic field, this small gap was rapidly suppressed, revealing a larger gap with a small weight that persisted up to a higher upper critical field (Physica C 385 (2003) 244).

Figure S3 presents the spectra at 1.2 GPa with two-gap fits at different temperatures, but neither the data nor the fits are tabulated or compared with the gaps at 0.7 GPa. This comparison is necessary.

Additionally, there is an incorrect statement in Section II, Results:
"These results are consistent with the previously reported topologically nontrivial superconductivity under ambient pressure [Refs. 30,31]."
However, Ref. 31 presents only single-gap spectra with $2\Delta/k_BT_c \sim 3.7$, and Ref. 30 explicitly states that the μSR experiment lacks phase/sign-of-the-gap resolution, merely speculating about topological issues based on sensitivity to disorder. Therefore, the authors should be more cautious when making claims about topologically nontrivial superconductivity.

On superconductivity in monoclinic 1T′ phase

At the pressure above 1.4 GPa in the spectrum a central peak emerges in the PCAR spectra.
In the revised manuscript, the authors perform a thorough analysis of the possible origins of the zero-bias conductance peak and conclude that s+p-wave gap is the most probable explanation for the spectra taken at 2.2. GPa. At the same time, they show that p-wave component is not intrinsic but rather induced from bulk to surfaceas evidenced by the similar temperature and field behavior of the s and p components.

Fig. 3(d) presents the spectrum after subtracting the s-wave contribution, but the resulting p-wave component appears very different from that shown in Fig. 2(c)

Also question arises what happened to the two s-wave gaps observed in the orthorhombic Td phase?

On page 8 it is stated "However, an anisotropic s-wave scenario is improper due to the ill-fitting, as shown in Fig. S4(a).". However, it appears that Fig. S4(a) actually uses an isotropic s-wave gap, not anisotropic one.

Additionally, Figures 2 and S5 are identical.

Reviewer #3

(Remarks to the Author)
The authors report on high-pressure point-contact Andreev reflection (PCAR) spectroscopy measurements in $MoTe_2$, aiming to uncover unconventional superconductivity and possible higher-order topological phases. In particular, they claim the observation of two-gap s-wave superconductivity in the Td phase and proximity-induced surface p-wave superconductivity in the high-pressure 1T′ phase, further supported by theoretical modeling suggesting the emergence of third-order topological superconductivity (TOTSC) and associated Majorana hinge modes. The manuscript addresses an important and timely topic at the interface of superconductivity and topological materials. The experimental technique, soft point-contact spectroscopy under quasi-hydrostatic pressure, is technically demanding and well-suited for exploring pressure-induced phases. However, I have several concerns regarding the robustness of the conclusions and the strength of the evidence presented.

The main experimental claim of the manuscript is the observation of a zero-bias conductance peak (ZBCP) in the 1T′ phase, interpreted as evidence for surface p-wave superconductivity. While the BTK fits using an s + p-wave model can reproduce the ZBCP, the distinction from alternative multigap scenarios—such as two s-wave gaps with a very small Δ_2—is not fully convincing. As Referee 2 also noted, ZBCPs can arise in multigap superconductors without requiring unconventional pairing. The authors argue that the observed ZBCP is inconsistent with such a scenario based on the gap-size ratios and their temperature dependence. While this interpretation is plausible, it would be significantly strengthened by additional diagnostics, such as angular conductance anisotropy or impurity sensitivity, to more rigorously constrain the pairing symmetry. Furthermore, in Fig. 2(c), the authors apply a two-gap s + p-wave model, which introduces a large number of fitting parameters. This raises concerns about potential overfitting and reduces the credibility of the analysis. What are the fitting uncertainties? Are the fits robust under reasonable variation of parameters? Additionally, to dismiss other possible origins of the ZBCP, such as heating effects, the authors argue that "in this case, the ZBCP is generally broad and exhibits sensitivity to weak magnetic fields due to the critical current and high normal-state resistivity…" This statement is not sufficiently compelling. The entire dataset appears to be derived from a single sample. Given the central claim regarding surface p-wave superconductivity, it is essential to demonstrate reproducibility. I strongly recommend that the authors provide data from at least one additional sample to rule out sample-specific or contact-dependent artifacts.

The observed suppression of both s- and p-wave features under relatively modest magnetic fields is used to argue that the p-wave gap is induced via proximity to bulk s-wave superconductivity, not intrinsic. This is a reasonable interpretation and consistent with the theoretical model. However, the fragility of the ZBCP under field also means that the associated topological protection (if any) is weak, and further experiments would be necessary to confirm the unconventional nature of the state.

The theoretical analysis is technically sound and comprehensive, involving a BdG treatment of a SOTI band structure and demonstrating how a third-order topological superconducting state can emerge. However, no direct experimental evidence of hinge or corner-localized states is presented. The claim of Majorana corner states therefore remains speculative and should be framed accordingly—as a theoretical proposal rather than an experimental observation.

While the manuscript presents intriguing results and offers a novel perspective on pressure-induced topological superconductivity in $MoTe_2$, I find that the central claims—particularly those concerning p-wave superconductivity and higher-order topology—are not yet supported by sufficiently direct experimental evidence. Although the observed zero-bias conductance peak (ZBCP) is suggestive, it is not unique to p-wave pairing, and no spatially resolved measurements of hinge or corner states are provided. The theoretical framework is rigorous, but remains predictive rather than demonstrative. I cannot support publication in its current form, though I believe the work may be suitable after substantial experimental reinforcement.

Version 2:

Reviewer comments:

Reviewer #1

(Remarks to the Author)
The authors have addressed the most of issues I raised, corrected the mistakes and made several changes to the manuscript. With this I recommend it for publication in Nature Communications.

Reviewer #3

(Remarks to the Author)
The authors have undertaken a substantial revision of the manuscript and provided a thorough and detailed response to the previous reports. The revised version is significantly clearer and better organized, and the distinction between experimentally established results and theoretically motivated interpretations has been sharpened. Overall, my concerns have been satisfactorily addressed, and I support the publication of this work.

Open Access This Peer Review File is licensed under a Creative Commons Attribution 4.0 International License, which permits use, sharing, adaptation, distribution and reproduction in any medium or format, as long as you give appropriate credit to the original author(s) and the source, provide a link to the Creative Commons license, and indicate if changes were made.
In cases where reviewers are anonymous, credit should be given to 'Anonymous Referee' and the source.
The images or other third party material in this Peer Review File are included in the article's Creative Commons license, unless indicated otherwise in a credit line to the material. If material is not included in the article's Creative Commons license and your intended use is not permitted by statutory regulation or exceeds the permitted use, you will need to obtain permission directly from the copyright holder.
To view a copy of this license, visit https://creativecommons.org/licenses/by/4.0/

Response to Referee 1

The manuscript "Evidence of surface p-wave superconductivity and higher-order topology in $MoTe_2$" by Lee et al. addresses a non-conventional superconductivity in two topological phases controlled by the pressure in the transition metal dichalcogenide $MoTe_2$. The subject is hot and important. The paper based on the point-contact Andreev reflection (PCAR) spectroscopy measurements at high pressures which is a demending technique concludes that the lower pressure T_d phase is characterized by two superconducting energy gaps of $s^{\pm}$ symmetry while the higher pressure $1T'$ phase presents a contribution of p-wave surface superconductivity along with s-wave bulk component. The experiment of the soft point contact at high pressure is interesting and generally capable to shed a light on the superconducting parameter in such a non-trivial system but the quality of the obtained data and their analysis do not provide a firm evidence on the claimed superconducting order parameters which is needed in a such high profile journal. Then, I do not recommend a publication of the paper. The issues are the following

Thank you for your comprehensive review and insightful feedback on our manuscript exploring the superconducting order of $MoTe_2$. We appreciate your acknowledgment of the importance of our work in advancing the understanding of this extensively studied compound and its superconducting order parameter, stating *"addresses a non-conventional superconductivity in two topological phases controlled by the pressure in the transition metal dichalcogenide $MoTe_2$. The subject is hot and important."* Referee 1 has specifically requested an analysis of the superconducting order parameter for T_d and $1T'$. In response, we present a comprehensive analysis detailing our approach and supporting evidence, leading us to conclude that the superconducting gap in the T_d phase exhibits a two-gap structure, while the $1T'$ phase shows proximity-effect-induced surface $s + p$ superconductivity.

1. The double conductance peaks in the differential conductance observed at 0.7 GPa below T_c, as shown Fig. 2(a), is consistent with the single s-wave SC gap but also with two gaps with the gaps ratio $2\Delta/k_BT_c$ of 3.2 and 1.4. Obviously the more fit parameters the better fit but here a single gap is apparently satisfactory and the two gaps has unusual strength. In two gaps superconductors one gap is usually smaller and another one larger than the BCS limit.

Thank you for your valuable feedback. We agree with the reviewer's suggestion that using more parameters could have enhanced the clarity of our experimental results, and a single gap is satisfactory for describing temperature evolution differential conductance. However, at 0.7 GPa, as shown in Figure S2 in Supplemental Information, the application of a magnetic field indeed confirmed the presence of two distinct gaps, which is not explained by single gap superconductivity. Moreover, other point contact spectroscopy and penetration experiments at ambient pressure report a two-gap nature in the T_d phase [R1, R2]. Therefore, our analysis utilized a two-gap model, with the fitting results presented in the Supplementary Information. In particular, the magnitudes of the two gaps are similar with the other work [R1, R2].

Based on this reply, we provide the detailed parameters of the two-gap model in Tables S2 and S3 of the Supplementary Information.

[R1] Guguchia, Z. et al. Signatures of the topological $s^{\pm}$ superconducting order parameter in the type-II Weyl semimetal T_d-$MoTe_2$. Nature Communications **8**, 1082 (2017).

[R2] Naidyuk, Y. et al. Surface superconductivity in the Weyl semimetal $MoTe_2$ detected by point contact spectroscopy. 2D Materials **5**, 045014 (2018).

2. For the two-gap BTK fit all other parameters as Z_i, Γ_i, weight of particular gap as well as some measure of quality of the two gap fit in comparison with the single-gap one are missing.

Thank you for this comment. We added the detailed fitting parameters in Tables S1, S2, and S3 of the Supplementary Information for one- and two-gap models.

3. Indeed, the shape of the spectra taken at 0.5 kG reminds a presence of two gaps (the same holds for Fig. S2(b) but again any quantitative analysis of the fit or even fit is missing (SI). As a paradox, the experimental data taken at 0.5 kG seems to be more "two-gap like" than the fit, but without the fit parameters, it is difficult to make any conclusions.

Thank you for this comment. As commented in Reply 1, the two gap features are similar to previous works [R1,R2], and we are glad that the Referee agrees with our claim of a two-gap system. For clarity, in accordance with the comment, we have included the fitting parameters in Tables S1-S3 of the Supplementary Information. The detailed fitting model is described in Sec. S1.5 of the Supplementary Information [R3, R4].

In particular, for the magnetic-field-dependent PCAR data and their two-gap features, the revised manuscript demonstrates the raw data and its fitting at Fig. S2(b), where the explicit fitting parameters can be seen in Table S3. Therefore, the main characteristics of the two-gap feature are well-captured. Note that Fig. S2 in the revised manuscript was Fig. 2 in the previous manuscript.

Regarding the paradox related to the more "two-gap like" data, we agree there exists a subtle deviation between experimental data and fit at 0.5 kG. However, because the main characteristics of the two-gap feature for various ranges of magnetic fields are well-captured in our analysis, we do not consider such details, which is beyond our work.

[R3] Daghero, D. and Gonnelli, R. Probing multiband superconductivity by point-contact spectroscopy. Superconductor Science and Technology **22**, 043001 (2010).

[R4] Daghero, D., Tortello, M., Ummarino, G. and Gonnelli, R. Directional point-contact Andreev-reflection spectroscopy of Fe-based superconductors: Fermi surface topology, gap symmetry, and electron–boson interaction. Reports on Progress in Physics **74**, 124509 (2011).

4. The field dependence of two gaps in Fig. 2(c) is generally not correctly obtained as the junction is obviously in the vortex state which is difficult to model.

We thank the Referee for this comment. We acknowledge the inherent challenges in accurately modeling the gap variations under magnetic fields for a type II superconductor. We agree that it is hard to describe magnetic-field-induced gaps in the presence of vortices. However, we intended to provide a comparative analysis of the gap as a function of a magnetic field to guide eyes using the conventional fitting, $\Delta \sim (H_c\text{-}H)^{1/2}$ used in various works of literature [R5,R6]. Furthermore, the specific influence of magnetic fields on the gap in a type II superconductor is a substantial topic in its own right and falls outside the scope of our current study. Moreover, as discussed in Reply 3, Fig. 2(c) intended to show consistency with the reported two-gap nature [R1,R2] and somewhat dilutes our main claim regarding the emergence of a p-wave pairing on the surface of the $MoTe_2$. Therefore, we move Fig. 2(c) to Fig. S2(c) and the related discussion to the Supplementary Information. We also added an explanation regarding the information fitting in the caption.

We have added Ref [R5,R6] and revised the caption of Fig. S2(c) as follows:

(c) Two superconducting gaps as a function of the magnetic field. The dashed lines are fit with $\Delta \sim (H_c\text{-}H)^{1/2}$. The inset shows two superconducting gaps as a function of temperature. The solid and dashed lines represent temperature-dependent gaps as a function of temperature from one-gap and two-gaps BCS models, respectively.

[R5] Morenzoni E. et al., Field dependence of the superconducting gap inYPd_2Sn: a μSR and NMR study J. Phys.: Conf. Ser. **551** 012027 (2014).

[R6] Gross F. et al., Anomalous temperature dependence of the magnetic field penetration depth in superconducting UBe_{13} Z. Phys. B **64** 175 (1986).

5. The temperature dependence of the two gaps given in the inset of Fig. 2(c) lacks any explanation how the intraband and interband couplings are accounted for in the fit.

Thank you for this valuable comment. We agree that, as the Referee commented, there was a lack of explanation of how the intraband and interband couplings are accounted for in the fit. For the detail explanation about the temperature dependence of the two gaps, given in the inset of Fig. 2(c), we added Ref [R7,R8] with an explanation in the Supplemental information, as shown below.

"Within the framework of the BCS model, the general formula for the superconducting gap as a function of temperature is represented by the following expression:

$$\Delta_k = -\Sigma_l \omega_{kl} \frac{\Delta_l}{2E_l} \tanh \frac{\beta E_l}{2} \quad \text{(R1)}$$

where $E_l = (\xi_l^2 + \Delta_l)^{1/2}$, ω_{kl} is the interaction potential, and $\beta = 1/k_b T$, respectively.
For two-gaps superconductors, temperature-dependent gap follows the below simpler expression:

$$\begin{bmatrix} \Delta_1 \\ \Delta_2 \end{bmatrix} = \frac{1}{2} \begin{bmatrix} \omega_{11} & \omega_{12} \\ \omega_{21} & \omega_{22} \end{bmatrix} \begin{bmatrix} \Delta_1 \tanh \frac{\beta E_1}{2} / E_1 \\ \Delta_2 \tanh \frac{\beta E_2}{2} / E2 \end{bmatrix} \quad \text{(R2)}$$

where ω_{ii} and ω_{ij} are intraband and interband coupling when $i, j \in \{1, 2\}$, $i \neq j$. Δ_1 and Δ_2 can be calculated numerically.
Futhermore, for conventional single-gap superconductors ($\omega = \omega_{kl}$ and $\Delta = \Delta_l = \Delta_k$), temperature-dependent gap follows the below simpler expression:

$$\frac{1}{N(0)} = \omega \int_0^{\hbar\omega_c} \frac{\tanh \frac{1}{2}\beta(\xi^2 + \Delta^2)^{1/2}}{(\xi^2 + \Delta^2)^{1/2}} \mathrm{d}\xi \quad \text{(R3)}$$

where N_0 and w_c are the density of states at the Fermi level for electrons of one spin orientation and phonon spectrum cutoff, respectively. For weak-coupling $\hbar w_c \gg k_b T_c$. Δ can be calculated numerically."

The fitting parameters ω_{kl} are $\omega_{11} = 0.39$, $\omega_{12} = \omega_{21} = 0.005$, and $\omega_{22} = 0.29$, which is given in Sec. 1.2 in the revised Supplementary Information.

[R7] Suhl, H. and Matthias, B. T. and Walker, L. R Bardeen-Cooper-Schrieffer theory of superconductivity in the case of overlapping bands. Physical Review Letter **3**, 552, (1959)

[R8] Tinkham, M. Introduction to Superconductivity (mMcGraw-Hill Book, 1996)

6. The temperature dependence of the upper critical magnetic field: Any details on experimental data are missing, how obtained, how defined. There is good fit by two gaps. Is there any distinction between $s^{\pm}$ and s^{++}? Even if we accept a two gap superconductivity in the T$_d$ phase, how can the authors distinguish their $s^{\pm}$ and s^{++} version? They just recall Refs. 27 and 28 to support $s^{\pm}$.

We thank the referee for this valuable comment. Regarding the details of the temperature dependence of the upper critical magnetic field, we have provided more detailed fitting information regarding fitting parameters and their meaning in Sec. S1.4 in the Supplementary Information.

About the issue regarding distinguishing the $s^{\pm}$ and s^{++} gap symmetries, it remains challenging and currently has not been distinguished directly in this material. For the point-contact spectroscopy experiment, the phase-sensitive measurement is necessary to distinguish the two phases, which is cutting edge and has yet to be reported experimentally. Consequently, we also cannot confirm that the gap is neither $s^{\pm}$ nor s^{++}, but only that the system is a two-gap system. Our data verifies the two-gap nature of the T$_d$ phase, providing critical evidence for the reliability of our pressure-dependent experiments. This observation underscores the significance of confirming the two-gap nature of the T$_d$ phase as a means to validate the effectiveness of pressure testing methodologies.

Therefore, we moved the detailed discussion related to the two-gap feature and upper critical field to Sec. S1.4 in the Supplementary Information, and we also moved Fig. 2(c) to Fig. S2(c). We added a reference [R1] showing the two-gap nature, which further discusses the s^{++} and $s^{\pm}$ states based on impurity dependence of the $MoTe_2$ for the general readers.

7. Fig. S1 in Supplementary Information claims temperature evolution but this is only the spectrum at 0.3 K with two undistinguishable fits.

Thank you for this comment. The caption regarding the figure was a typo, we intended to show the results of the T_d phase at 0.3 K. Therefore, we have revised the caption of Fig. S1 as follows:

"The differential conductivity of $MoTe_2$ at 0.7 GPa in the T_d phase normalized by the 3.8 K data. The red and blue lines are best least-square fit with s-wave one-gap and two-gap at 0.3 K and 0 kG."

8. At higher pressure above 1.2 GPa (Fig. 3 (d)) a new type of spectra have been observed with the zero-bias conductance peak (ZBCP) which seems to be neither from a thermal effect nor an effect of magnetic impurity (no split under the field). But ZBCP is rapidly suppressed by a relatively small magnetic field of 1 kG (0.1 T). If this is due to a p-wave superconductivity it is strange.

Thank you for this valuable comment. In p-wave superconductors, the spin-triplet pairing typically results in an unusually high upper critical field (H_{c2}), as observed in heavy fermion superconductors. However, in the case of p-wave superconductivity in $MoTe_2$, it is induced on the surface via the proximity effect from the bulk s-wave superconductivity, as shown in Figure 1(b) of the main manuscript. This indicates that the surface p-wave pairing cannot exist if there is no bulk s-wave pairing. Therefore, our experimental results that show the suppression of the ZBCP at a relatively small magnetic field are consistent with our theoretical analysis. We have revised the manuscript to emphasize this notion by adding the following paragraphs.

"We now investigate the physical properties of the emerging p-wave superconductivity in the $1T'$ phase. First, the p-wave superconductivity is closely tied to bulk s-wave superconductivity via the proximity effect, as illustrated in Fig.1. Experimental evidence demonstrates that s- and p-wave superconducting gaps exhibit strikingly similar temperature-dependent behaviors, as shown in Fig. 3(c). Both gaps decrease with increasing temperature and vanish at the same T_c, indicating their intrinsic connection. Moreover, the s- and p-wave superconductivity also exhibit similar magnetic field responses. For this, we distinguish the contribution of s- and p-wave superconductivity. Figure 3(b) depicts the magnetic field-dependent PCAR spectra, which are fitted using a single s-wave BTK model (solid lines), implying the suppression of s-wave superconductivity under a magnetic field. On the other hand, figure 3(d) represents residual p-wave contributions after removing the s-wave superconductivity. Finally, the inset in Fig. 3(d) summarizes a magnetic field-dependent s-wave superconducting gap (black solid squares) alongside the magnitude of the ZBCP, a hallmark of p-wave superconductivity (blue open diamonds). They are gradually suppressed by the magnetic field and completely suppressed near 15 kG. Unlike intrinsic spin-triplet p-wave superconductivity, which is robust against magnetic fields, the observed p-wave pairing vanishes more easily, further confirming its induced nature. These observations indicate that p-wave feature is strongly correlated with the s-wave superconductivity, irrespective of tuning factors such as temperature and magnetic field.

Note that the role of symmetry is critical in stabilizing the p-wave superconductivity. The ZBCP observed in the inversion-symmetric $1T'$ phase disappears in the inversion symmetry-broken T_d phase, as shown in Figs. 2(a), S2, and S3. Also, the magnetic field easily breaks the p-wave superconductivity, as shown in Fig. 3(c,d). This indicates that inversion and time-reversal symmetries play a fundamental role in protecting the p-wave superconductivity, in agreement with the theoretical predictions below."

9. The authors analyze different gap symmetries in the Supplementary Information and compare the simulated spectra with the measured ones. The main conclusion is that the best fit is by a combination of the s-wave gap and the p-wave gap (Fig. 3(a), black, not blue lines), but the details of this fit are missing. There is no trace of two gaps in the form of two pairs of peaks in the raw data. What is the temperature dependence of these two gaps, do they have a common and what T_c? Etc.

Thank you for this valuable comment. The detailed fitting results, illustrating the temperature-dependent evolution of the ZBCPs, are provided in Fig. 3(a) of the revised manuscript, and the detailed numerical values are provided in Table S4 in the Supplementary Information. The evolution of p-wave and s-wave gaps with respect to temperature is given in Fig. 3(c) of the revised manuscript, which shows that they share the same T_c. This implies that the two gaps are closely related, which is consistent with the theoretical results, as mentioned in response to comment 8, the p-wave pairing is induced via the proximity effect between the s-wave superconductor and the SOTI. Such proximity-induced p-wave pairing vanishes when there is no bulk s-wave pairing, resulting in the same T_c.

According to this reply, we have revised Fig. 3 and the manuscript as follows on page 12:
"Therefore, we theoretically demonstrate that both s- and p-wave surface superconductivity are induced by the bulk s-wave superconductivity, as illustrated in Fig. 1. Notably, the p-wave pairings induced in the SOTI arise from the interplay between the trivial bulk s-wave superconductivity and the topological surface states, which are protected by inversion and time-reversal symmetries. Thus, if the bulk s-wave gap, $\Delta_{s\text{-wave}}$, vanishes, no proximity-induced pairings can exist as all the induced bulk pairings are proportional to $\Delta_{s\text{-wave}}$ (see detailed form in Sec. S2.2.2 of SI). This theoretical analysis aligns with the experimental results in Figs. 2 and 3, where both the p-wave and s-wave pairings vanish at the same temperature and are fragile under inversion symmetry breaking or an applied magnetic field."

Response to Referee 2

In this work, the authors studied the superconducting properties of the T_d phase and $1T'$ phase of $MoTe_2$. Using soft-point contact Andreev reflection spectroscopy, the authors claimed that they observed s±-wave pairing in the T_d phase. As pressure increases, the material undergoes a phase transition into the $1T'$ phase. In the $1T'$ phase, a zero-bias conductance peak was observed. Theoretical analysis further suggests that there are Majorana hinge modes in the $1T'$ superconducting phase.

- In the experiment, the presence of two-gap superconductivity is convincing. However, there is no experimental evidence that the pairing is $s^{\pm}$-wave. The observation of disorder-induced reduction of T_c in T_d-$MoTe_2$ (as observed in previous works) is not strong enough to claim that $MoTe_2$ is in the $s^{\pm}$-pairing state. When the charge carrier density is not high, it is reasonable to expect that T_c can be sensitive to changes in disorder strength. Based on the current experimental observations, the authors can only claim that T_d-$MoTe_2$ is a two-gap superconductor but cannot claim that it is a $s^{\pm}$-wave superconductor.

- The second major experimental finding of the authors is the appearance of the zero-bias conductance peak observed in the $1T'$ phase. The authors claim that the zero-bias conductance peak can be explained by $s + p$-wave pairing. I am not convinced by this claim. Zero-bias peaks can be observed in many multigap superconductors. In the fitting, I suspect that the authors used a small p-wave pairing gap and a large s-wave gap in the model calculation. In this case, the zero-bias peak emerges because of the smallness of the p-wave gap. In a multigap superconductor, a zero-bias peak can emerge without p-wave pairing. When one pairing gap from some Fermi pockets is much smaller than the other pairing gap from other Fermi pockets, a zero-bias peak can emerge. I am not convinced that the observed zero-bias conductance peak was induced by $s + p$-wave pairing.

- Furthermore, the authors claimed that there are Majorana hinge modes in the material. There is no evidence for this claim. To claim the existence of hinge states, one probe the tunnelling conductance at the hinges. This kind of experiments are not performed by the authors.

The experimental observations of the authors are not entirely trivial. However, I am not convinced that there is evidence of p-wave superconductivity in $MoTe_2$. There is also no evidence of higher-order topology in the material. I do not recommend the publication of this work in Nature Communications.

We thank the Referee for their detailed feedback on our manuscript. We greatly appreciate the acknowledgment that “the experimental observations of the authors are not entirely trivial.” This recognition underscores the value of our experimental results in advancing the understanding of $MoTe_2$, a material of significant interest in the condensed matter physics community.

In response, we provide a thorough analysis of our methodology and evidence, which support our conclusion that the superconducting gap in the T_d phase demonstrates a two-gap structure, while the $1T'$ phase exhibits proximity-effect-induced surface $s + p$-wave pairing characteristics. We hope the detailed clarification provides further support for the robustness of our findings.

1. In the experiment, the presence of two-gap superconductivity is convincing. However, there is no experimental evidence that the pairing is $s^{\pm}$-wave. The observation of disorder-induced reduction of T_c in T_d-$MoTe_2$ (as observed in previous works) is not strong enough to claim that $MoTe_2$ is in the $s^{\pm}$-pairing state. When the charge carrier density is not high, it is reasonable to expect that T_c can be sensitive to changes in disorder strength. Based on the current experimental observations, the authors can only claim that T_d-$MoTe_2$ is a two-gap superconductor but cannot claim that it is a $s^{\pm}$-wave superconductor.

We appreciate your insightful comments, including the remark "In the experiment, the presence of two-gap superconductivity is convincing." As noted by Referee 2, distinguishing between the s^{++} and $s^{\pm}$ gap symmetries remains a significant challenge given the limitations of our current dataset. Our focus regarding the gap of $MoTe_2$ in the T_d phase is not to show that the system's pairing will be $s^{\pm}$ pairing, but that it is a two-gap feature as the Referee points out. We intended that our data verifies the two-gap nature of the T_d phase, providing critical validation for the reliability of our pressure-dependent experiments. Regarding the disorder-induced reduction of the T_c, which is discussed in the introduction, we agree with the Referee that it is not strong enough to claim that the system will have $s^{\pm}$ pairing.

Based on this reply, we have erased the discussion on the $s^{\pm}$ pairing from the abstract and main text.

2. The second major experimental finding of the authors is the appearance of the zero-bias conductance peak observed in the 1T′ phase. The authors claim that the zero-bias conductance peak can be explained by $s + p$-wave pairing. I am not convinced by this claim. Zero-bias peaks can be observed in many multigap superconductors. In the fitting, I suspect that the authors used a small p-wave pairing gap and a large s-wave gap in the model calculation. In this case, the zero-bias peak emerges because of the smallness of the p-wave gap. In a multigap superconductor, a zero-bias peak can emerge without p-wave pairing. When one pairing gap from some Fermi pockets is much smaller than the other pairing gap from other Fermi pockets, a zero-bias peak can emerge. I am not convinced that the observed zero-bias conductance peak was induced by $s+p$-wave pairing.

We thank Referee 2 for their valuable comment. Regarding the fitting data, we have revised the manuscript such that the size of the pairings can be seen. According to the fitting parameters, the two pairings are comparable. This shows that the ZBCP is not formed accidentally, but due to a physical process. According to our theoretical analysis, p-wave pairing is induced from the bulk s-wave, and therefore s- and p-wave pairings coexist in 1T′ phase.

If the p-wave pairing is small, there could be a different mechanism for the formation of the ZBCP. As the referee pointed out, in the case of multi-gap superconductivity, the formation of a ZBCP can indeed occur when there is a very small s-wave gap coexisting with a relatively large s-wave gap at relatively high temperature. However, this scenario is theoretically feasible only when the difference between the two gaps is extremely large (the size of large gap is seven times larger than that of the small gap at 3.3 K [see Fig. R1(a)]). However, as shown in Fig. 3(c), at 2.2 GPa, the p-wave gap is 75% of the size of the s-wave gap.

For our experiment, the gap size difference is less than a factor of seven, and the temperature of samples is as low as 0.3 K, such an observation becomes highly unlikely [see Fig. R1(b)]. The possibility of a zero-bias conductance peak (ZBCP) arising from a small gap, as suggested by the Referee, can be excluded based on our analysis. Furthermore, we tested various gap structures and performed the corresponding fittings [See Fig. S4-S6]. Our results indicate that the $s + p$-wave fitting provides the most plausible explanation.

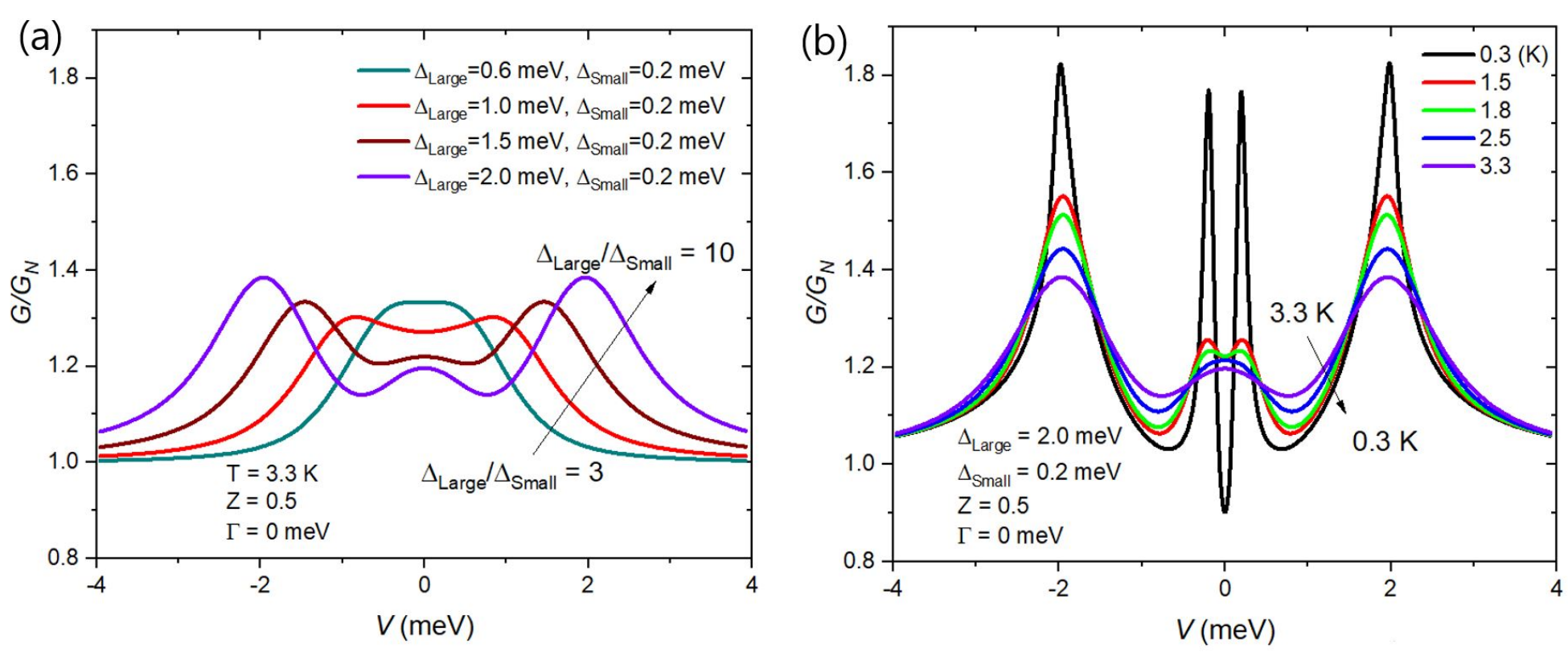

Fig. R1. **(a)** Simulated BTK model for two s-wave gaps depending on $\Delta_{\mathrm{Large}}/\Delta_{\mathrm{Small}}$. **(b)** Simulated BTK model for two s-wave gaps depending on temperature for $\Delta_{\mathrm{Large}}/\Delta_{\mathrm{Small}} = 10$.

3. Furthermore, the authors claimed that there are Majorana hinge modes in the material. There is no evidence for this claim. To claim the existence of hinge states, one probe the tunnelling conductance at the hinges. This kind of experiments are not performed by the authors.

We thank the Referee for the insightful comment. We acknowledge the critical importance of experimental validation in substantiating claims regarding the existence of Majorana hinge modes.

Our principal claim centers on the demonstration of surface p-wave superconductivity induced via the proximity effect in 1T′-$MoTe_2$. This claim is strongly supported by our experimental data, which reveal a zero-bias conductance peak—a hallmark signature of p-wave superconductivity. Furthermore, we observed that this p-wave superconductivity is fragile under a relatively small magnetic field, consistent with the notion that it arises from bulk s-wave superconductivity. This behavior contrasts with conventional spin-triplet p-wave superconductivity, which is generally more robust against magnetic fields. Additionally, we report that the s-wave and p-wave superconducting gaps share the same critical temperature, which is in agreement with our theoretical predictions. These observations provide robust evidence for the emergence of p-wave superconductivity through the proximity effect, as supported by systematic theoretical analysis.

Regarding the Majorana states, as the Referee pointed out, direct experimental evidence such as tunneling conductance measurements is indeed required to confirm their existence. However, performing such measurements under the extremely high-pressure conditions used in our study remains a significant experimental challenge.

In the absence of such direct evidence, we have included an additional theoretical analysis to demonstrate the potential emergence of Majorana states in this system. Using the Schrieffer-Wolff transformation, we analytically derived the induced $s + p$-wave pairings arising from the proximity effect. We further analyzed the hinge dispersion of the system through the surface Hamiltonian, finding excellent agreement between our analytical results and tight-binding calculations. Furthermore, we found that the resulting induced pairings give Majorana corner states with a topological $\mathbb{Z}_8$ index, which matches our new calculations. We have updated Figure 4 in the manuscript to comprehensively include all pairings induced by the proximity effect.

We acknowledge the theoretical nature of our claim regarding Majorana states and have revised the manuscript to reflect a more measured perspective. Specifically, we now present 1T′-$MoTe_2$ as a promising platform for realizing higher-order Majorana modes, while emphasizing that experimental validation remains an open challenge. To align with this refined claim, we have modified the title of the manuscript to:

"Surface p-wave Superconductivity and Higher-Order Topology in $MoTe_2$."

The abstract also reflects this when regarding the potential Majorana states as follows:

"Moreover, we suggest that the topological hinge states, derived from second-order topological bands, evolve into zero-energy Majorana corner states in this proximity-effect-induced third-order topological superconducting phase."

Relevant sections of the manuscript have also been updated to clarify and temper the discussion accordingly.

List of Changes:

The manuscript has been diligently revised to be in line with our responses.

1. Title: Changed to "Surface p-wave Superconductivity and Higher-Order Topology in $MoTe_2$" in accordance to Referee 2's comments.

2. Authors: No change.

3. Abstract: The abstract has been changed to account for the changes in the main text.

4. Main text:

   (a) We have moved the section regarding the T_d phase (originally subsection A of the Results section) to the Supplementary Information.

   (b) Regarding the surface p-wave pairing in the 1T′ phase, we have highlighted experimental results that indicate that the p-wave pairing is induced via the proximity effect.

   (c) A new discussion has been included in the Results section. This discussion shows what pairing is induced via the bulk-to-surface proximity effect.

   (d) The induced pairings suggest the superconducting state of the 1T′ phase as a third-order topological superconducting system. Therefore, we have changed the theoretical discussion to show that Majorana corner states emerge using numerical methods and have also introduced a topological index, which shows that the system is indeed a third-order topological system.

   (e) We have changed the tone of the manuscript to be more mild and suggestive regarding the emergence of Majorana states in accordance with Referee 2's comments.

5. Figures:

   (a) Fig. 1 has been updated to show the proximity effect in the real and reciprocal space.

   (b) Fig. 2 has been moved to the Supplementary Information.

   (c) A new Fig. 2 has been included, which shows the phase transition from the T_d to 1T′ phase and the superconducting pairings in the 1T′ phase.

   (d) Fig. 3 has been updated in accordance with the Referees' comments.

   (e) Fig. 4 has been updated by changing the model of the tight-binding calculations and showing the topological index regarding the Majorana corner states.

6. References: The following references have been added to the manuscript:

[14] Lane, C. & Zhu, J.-X. Identifying topological superconductivity in two-dimensional transition-metal dichalcogenides. Physical Review Materials **6**, 094001 (2022).

[15] Schrade, C. & Fu, L. Nematic, chiral, and topological superconductivity in twisted transition metal dichalcogenides. Physical Review B **110**, 035143 (2024).

[16] Xu, G., Wang, J., Yan, B. & Qi, X.-L. Topological superconductivity at the edge of transition-metal dichalcogenides. Physical Review B **90**, 100505 (2014).

[17] Bahramy, M. et al. Ubiquitous formation of bulk Dirac cones and topological surface states from a single orbital manifold in transition-metal dichalcogenides. Nature materials **17**, 21–28 (2018).

[34] Pan, Z.-C. et al. Altshuler-Aronov-Spivak interference of one-dimensional helical edge states in $MoTe_2$. Physical Review B **107**, 045411 (2023).

[35] Chen, P. et al. Asymmetric edge supercurrents in $MoTe_2$ Josephson junctions. Nanoscale Advances **6**, 690–696 (2024).

[36] Huang, S.-J., Park, K. and Hsu, Y.-T. Hybrid-order topological superconductivity in a topological metal $1T'$-$MoTe_2$. npj Quantum Materials **9**, 21 (2024).

[37] Sasaki, S. et al. Topological superconductivity in $CuBi_2Se_3$. Physical review letters **107**, 217001 (2011).

[38] Kallin, C. Chiral p-wave order in Sr_2RuO_4. Reports on Progress in Physics **75**, 042501 (2012).

[40] Lutchyn, R. M., Sau, J. D. & Das Sarma, S. Majorana fermions and a topological phase transition in semiconductor-superconductor heterostructures. Physical review letters **105**, 077001 (2010).

[42] Qin, W., Gao, J., Cui, P. & Zhang, Z. Two-dimensional superconductors with intrinsic p-wave pairing or nontrivial band topology. Science China Physics, Mechanics & Astronomy **66**, 267005 (2023).

[54] Schrieffer, J. R.& Wolff, P. A. Relation between the Anderson and Kondo Hamiltonians. Physical Review **149**, 491 (1966).

[57] Haim, A. & Oreg, Y. Time-reversal-invariant topological superconductivity in one and two dimensions. Physics Reports **825**, 1–48 (2019).

[58] Zhang, F., Kane, C. & Mele, E. Time-reversal-invariant topological superconductivity and majorana kramers pairs. Physical review letters **111**, 056402 (2013).

[61] Ahn, J. & Yang, B.-J. Higher-order topological superconductivity of spin-polarized fermions. Physical Review Research **2**, 012060 (2020).

[62] Khalaf, E., Po, H. C., Vishwanath, A. & Watanabe, H. Symmetry indicators and anomalous surface states of topological crystalline insulators. Physical Review X **8**, 031070 (2018).

7. Supplementary Information:
   (a) We have moved the section regarding the T_d phase and Fig. 2 of the main text to the Supplementary Information.
   (b) We have included Tables S1-S4 to show the parameters used for our fitting and experimental analysis in accordance with the Referees' comments.
   (c) We have changed the theoretical details. We analytically derived the proximity effect induced $s + p$-wave pairings and the Majorana corner states.

Response to Referee #1

The authors have addressed the issues raised in the initial reports and have made several changes to the manuscript, which certainly improve its quality. However, I do not believe the paper is ready for publication in Nature Communications.

We thank the Referee for carefully re-evaluating our revised manuscript and for recognizing that the changes have improved its quality. We understand that the remaining concern is whether the evidence now reaches the level required for publication in *Nature Communications*. In this second revision, we have therefore focused on strengthening the quantitative basis for multi-gap superconductivity in the T_d phase, more tightly constraining the origin of the $s+p$ superconductivity in the $1T'$ phase, and aligning the scope of our topological claims with what is firmly demonstrated by experiment.

1. Substantial Improvement of Scientific Content

- For the **T_d phase**, we have expanded the discussion of the **double-peak structure** in the Andreev reflection spectra [now presented in the main text with Fig. 2]. We provide a systematic analysis of its **multi-gap nature**, including temperature- and field-dependent data, detailed fitting parameters, and alternative results [Sec. S1.2]. These results are consistent with the multi-gap scenario is reported in muon-spin-rotation experiments.

- For the **$1T'$ phase**, we have added both experimental and theoretical analyses clarifying its distinct pressure-induced evolution and the emergence of **proximity-induced p-wave surface pairing** [reorganized Fig. 3 and related discussion in the main text, with quantitative analysis in Sec. S1.3].

- We have refined **a unified microscopic modeling** so that the calculated temperature and field dependence of the gaps for both phases reproduce the experimental results semi-quantitatively [Figs. 2 and 4].

2. Addressing Specific Reviewer Concerns

- We clarify that the observed BCS ratio in $MoTe_2$ values are compatible with conventional BCS limits when considering experimental uncertainties and the broad superconducting transition in $MoTe_2$. We also explain why the two-gap features are more apparent in field-suppressed spectra than in zero field, drawing analogies to MgB_2 under c-axis tunneling conditions.

- We now clearly distinguish experimentally observed signatures (multi-gap s-wave superconductivity in T_d and pressure-induced surface $s+p$ behavior in $1T'$) from theoretically motivated consequences (possible third-order topology and Majorana corner modes), and we state explicitly that the theory provides a consistent and predictive framework for interpreting the data rather than a direct demonstration of topological hinge or corner modes.

3. Scientific Significance

- In the T_d phase, the robust and reproducible **double-peak structure** under three independent tuning parameters (magnetic field, temperature, and pressure), together with the two-gap BTK and two-band BCS analysis, provides a consistent signature for multi-gap superconductivity generated with the type-II Weyl semimetal band structure.

- In the 1T′ phase, the selective appearance and evolution of the zero-bias near the conduction peak are **consistent with topologically assisted surface** $s{+}p$ **pairing** arising from the interplay between bulk s-wave superconductivity and higher-order topological boundary bands. Within this microscopic framework, 1T′-$MoTe_2$ emerges as a plausible route to a third-order topological superconductor in which hinge-localized Dirac states evolve into Majorana corner modes, while we explicitly acknowledge that we do not directly image these modes.

- All measurements were performed on a **high-quality crystal** while continuously tuning pressure, allowing us to follow the evolution from the T_d to 1T′ phases without introducing disorder from chemical substitution. This unified experimental design provides phase-mapping capabilities comparable to chemical doping, while tuning only the lattice via applied pressure. It minimizes extrinsic effects such as impurity scattering and variations in carrier concentration, enabling a clean interrogation of symmetry-driven changes in the superconducting and topological properties.

The manuscript and Supplementary Information have been updated accordingly, and a comprehensive *List of Changes* has been included.

In light of these focused and extensive revisions, we believe the manuscript now fully addresses the previous criticisms and is suitable for publication in *Nature Communications*.

Regarding the orthorhombic T_d phase, the authors now provide more detailed parameters for their two-gap fit and have moved most of this section to the Supplementary Information. Nevertheless, some uncertainties remain. In the tables, does γ represent the weight of a specific gap? If so, is it associated with the smaller gap in both cases—s-wave in the T_d phase (Tables S1 and S2) and s+p in the 1T' phase (Tables S3 and S4)? In Section S1.5, this weight is denoted as w—is this consistent?

We thank the Referee for the careful reading of our Supplementary Information and for pointing out the potential confusion in our notation. We have now clarified and standardised the symbols used in the two-gap BTK analysis as follows.

- **Weighting factor** w
  In all two-gap BTK fits (both the $s+s$ fits in the orthorhombic T_d phase and the $s+p$ fits in the 1T′ phase), the parameter w denotes the spectral weight of one of the two gap components in the total conductance,
  $$G(V) = w\, G_1(V) + (1 - w)\, G_2(V).$$
  The values of w is given explicitly in Sec. S1.1.3 and in Tables I-VII (Tables S1-S7) and are used consistently throughout the Supplementary Information. In the key datasets discussed in the main text (0.7 GPa in the T_d phase and 2.2 GPa in the 1T′ phase), we fix $w = 0.5$ to avoid unnecessary fitting freedom; thus w is not tied to the smaller or larger gap, but simply specifies a fixed relative contribution of the two components.

- **Symbol** γ **versus** Γ
  The symbol γ that appeared in an earlier version of Tables S1-S4 was a typographical error and was not intended to represent a gap weight. It should have denoted the quasiparticle lifetime broadening parameter, conventionally written as Γ in BTK analyses. Γ is defined explicitly in Sec. S1.1.1 In the revised Supplementary Information, we have corrected this and now use Γ uniformly for the broadening parameter in both the text and all tables.

For clarity, we have added an explicit section on "Fitting Methodology" in Sec. S1.1, including definitions of the quasiparticle broadening Γ and the weighting factor w in Secs. S1.1.1 and S1.1.3. In addition, we have added tables corresponding to all figures in the main manuscript, included the missing w values, and corrected all occurrences of γ to 'Γ' in Tables S1-S7.The relevant tables are listed below.

We hope that these updates fully remove any ambiguity in our notation and provide a clear correspondence between the figures and the fitting parameters.

Type	T (K)	Z	w (weighting factor)	$\Delta_{s,large}$ (meV)	$\Delta_{s,small}$ (meV)	Γ
s-wave (one-gap)	0.3	0.5	N/A	0.4	N/A	0.12
s+s wave (two-gap)	0.3	0.5	0.5	0.4	0.18	0.12

TABLE I. [Revised Table. S1] Fitting parameters for single-gap and two-gap s-wave BTK models used to analyze the normalized differential conductance of $MoTe_2$ at 0.3 K and 0.7 GPa in the T_d phase. Here, T is the measurement temperature, Z is the dimensionless barrier strength, w is the weighting factor between the two gaps, $\Delta_{s,\mathrm{large}}$ and $\Delta_{s,\mathrm{small}}$ are the large and small superconducting gaps, and Γ is the quasiparticle broadening parameter.

Type	T (K)	Z	w (weighting factor)	$\Delta_{s,\mathrm{large}}$ (meV)	$\Delta_{s,\mathrm{small}}$ (meV)	Γ
s+s wave (two-gap)	0.3	0.5	0.5	0.40	0.18	0.12
	0.9	0.5	0.5	0.40	0.10	0.12
	1.5	0.5	0.5	0.40	0.04	0.12
	1.8	0.5	0.5	0.37	0.02	0.12
	2.1	0.5	0.5	0.33	0.00	0.12
	2.5	0.5	0.5	0.22	0.00	0.12
	2.9	0.5	0.5	0.08	0.00	0.12
	3.3	0.5	0.5	0.00	0.00	0.12

TABLE II. [Revised Table. S2] Temperature-dependent fitting parameters of the two-gap s-wave BTK model for the normalized differential conductance of $MoTe_2$ at 0.7 GPa in the T_d phase. Here, Z is the dimensionless barrier strength, w is the weighting factor between the two gaps, and Γ is the quasiparticle broadening parameter.

Type	$\mu_0 H$ (kG)	Z	w (weighting factor)	$\Delta_{s,\mathrm{large}}$ (meV)	$\Delta_{s,\mathrm{small}}$ (meV)	Γ
s+s wave (two-gap)	0.0	0.5	0.5	0.40	0.18	0.12
	0.2	0.5	0.5	0.40	0.16	0.13
	0.4	0.5	0.5	0.39	0.15	0.20
	0.5	0.5	0.5	0.37	0.13	0.22
	1.0	0.5	0.5	0.25	0.00	0.22
	2.0	0.5	0.5	0.00	0.00	0.22

TABLE III. [Revised Table. S3] Magnetic-field-dependent fitting parameters of the two-gap s-wave BTK model for the normalized differential conductance of $MoTe_2$ at 0.3 K and 0.7 GPa in the T_d phase. As in Table S2, Z denotes the dimensionless barrier strength, w the weighting factor between the two gaps, and Γ the quasiparticle broadening parameter.

Type	T (K)	Z	w (weighting factor)	$\Delta_{s,large}$ (meV)	$\Delta_{s,small}$ (meV)	Γ
s+s wave (two-gap)	0.3	0.51	0.46	0.51	0.17	0.14
	0.6	0.51	0.46	0.5	0.17	0.14
	1.2	0.51	0.46	0.5	0.17	0.14
	1.8	0.51	0.46	0.46	0.15	0.14
	2.4	0.51	0.46	0.44	0.11	0.14
	3.0	0.51	0.46	0.4	0.03	0.14
	3.3	0.51	0.46	0.3	0	0.14
	3.6	0.51	0.46	0.17	0	0.14
	3.8	0.51	0.46	0	0	0.14

TABLE IV. [Revised Table. S4] Temperature-dependent fitting parameters of the two-gap s-wave BTK model for the normalized differential conductance of $MoTe_2$ at 1.2 GPa in the T_d phase. As in Table S2, Z denotes the dimensionless barrier strength, w the weighting factor between the two gaps, and Γ the quasiparticle broadening parameter.

Type	$\mu_0 H$ (kG)	Z	w (weighting factor)	$\Delta_{s,large}$ (meV)	$\Delta_{s,small}$ (meV)	Γ
s+s wave (two-gap)	0	0.51	0.46	0.51	0.17	0.14
	0.2	0.51	0.46	0.49	0.1	0.16
	0.4	0.51	0.46	0.36	0.15	0.21
	0.8	0.51	0.46	0.25	0.14	0.21

TABLE V. [Revised Table S5] Magnetic-field-dependent fitting parameters of the two-gap s-wave BTK model for the normalized differential conductance of $MoTe_2$ at 0.3 K and 1.2 GPa in the T_d phase. As in Table S2, Z denotes the dimensionless barrier strength, w the weighting factor between the two gaps, and Γ the quasiparticle broadening parameter.

Type	T (K)	Z	w (weighting factor)	Δ_p (meV)	Δ_s (meV)	Γ
s+p wave	0.3	0.6	0.5	0.4	0.6	0.24
	0.6	0.6	0.5	0.4	0.6	0.24
	1.2	0.6	0.5	0.41	0.59	0.24
	1.8	0.6	0.5	0.43	0.57	0.24
	2.4	0.6	0.5	0.4	0.5	0.24
	3	0.6	0.5	0.37	0.4	0.24
	3.3	0.6	0.5	0.3	0.28	0.24
	3.6	0.6	0.5	0.22	0.23	0.24
	4.2	0.6	0.5	0.01	0.01	0.24

TABLE VI. [Revised Table. S6] Temperature-dependent fitting parameters of the s+p-wave BTK model for the normalized differential conductance of $MoTe_2$ at 2.2 GPa in the $1T'$ phase. Z denotes the dimensionless barrier strength, w the weighting factor between the two gaps, and Γ the quasiparticle broadening parameter.

Type	T (K)	Z	w (weighting factor)	Δ_p (meV)	Δ_s (meV)	Γ_p/Γ_s
s+p wave	0.3	0.6	0.5	0.4	0.6	0.24
	0.3	0.6	0.5	0.40	0.58	0.31/0.33
	0.3	0.6	0.5	0.39	0.49	0.35/0.37
	0.3	0.6	0.5	0.37	0.39	0.37/0.39
	0.3	0.6	0.5	0.26	0.26	0.39/0.4
	0.3	0.6	0.5	0	0	0

TABLE VII. [Revised Table. S7] Magnetic-field-dependent fitting parameters of the s+p-wave BTK model for the normalized differential conductance of $MoTe_2$ at 2.2 GPa in the 1T′ phase. Z denotes the dimensionless barrier strength, w the weighting factor between the two gaps, and Γ the quasiparticle broadening parameter.

In my previous report, I raised concerns about the unusual coupling strength when both gaps are below the BCS limit. This point has not yet been discussed, even though I am unaware of any other examples of such behavior.

As the Referee correctly points out, in prototypical two-gap superconductors such as MgB_2, it is frequently observed that the smaller gap exhibits a reduced BCS ratio, while the larger gap can also deviate from the nominal single-band value. This behavior arises once finite interband coupling between the gaps is taken into account. In such systems, the superconducting transition temperature T_c is not associated with an individual gap but is instead determined by a cooperative instability involving both bands. Consequently, the gap amplitudes mutually influence each other: the larger gap partially sustains the smaller one, while the smaller gap affects the evolution of the larger gap near T_c, leading to a characteristic tail in the temperature dependence of the gaps, an effect that is particularly pronounced for the smaller gap [R1].

From an experimental perspective, the intrinsic critical temperatures associated with individual gaps may therefore differ from the experimentally defined T_c, which is typically extracted from transport or spectroscopic onset criteria. For $MoTe_2$ in the T_d phase, using the bulk T_c determined from the onset of superconducting features in temperature-dependent Andreev-reflection spectra, we obtain $2\Delta/k_BT_c \simeq 1.4$ and 3.2 for the smaller and larger gaps, respectively. If instead the onset temperature from resistivity measurements is used ($T_{c,\text{onset}} \sim 2.1$ K [R2,R3]), the larger-gap ratio increases to approximately 4.4. This demonstrates that the extracted value of $2\Delta/k_BT_c$ depends sensitively on the definition of T_c and the measurement method, reflecting an inherent experimental uncertainty.

Within this context, the measured ratios for $MoTe_2$ fall naturally within the conventional BCS range. As emphasized by Tinkham [R4], weak-coupling superconductors typically exhibit $2\Delta/k_BT_c$ values spanning approximately 3–4.5. While values near 3.53 are often quoted as the canonical BCS result, elemental superconductors such as Al is known to display ratios around 2.8–3.9 [R5], demonstrating that values below 3.53 are not uncommon in real materials.

More generally, in multiband (multi-gap) superconductors, deviations from the single-band BCS value $2\Delta/k_BT_c = 3.53$ are an expected consequence of finite interband coupling. Near T_c, the linearized gap equations show that the transition temperature is governed by the largest eigenvalue of the interband coupling matrix, corresponding to a collective pairing eigenmode involving all bands, which can enhance T_c beyond the intraband pairing strength of any individual band. By contrast, at zero temperature the nonlinear gap equations distribute the pairing amplitude among the bands according to the associated eigenvector, such that a stronger band partially sustains the gap of a weaker one. As a result, the individual zero-temperature gap amplitudes are reduced relative to the enhanced T_c, and the ratios $2\Delta_i(0)/k_BT_c$ can fall below the single-band BCS value without implying anomalously weak pairing, even within a weak-coupling BCS framework [R1].

Consistent with this picture, reduced effective BCS ratios have been reported in a variety of multi-gap superconductors, including $Nb_2Pd_{0.81}S_5$ (1.6 and 3.1) [R6], V_3Si (2.0 and 2.8) [R7], $FeSe_{0.5}Te_{0.5}$ (2.96) [R8], $LuNi_2B_2C$ (3.2) [R9], and 122-type iron pnitide superconductors (3.1) [R10]. These examples illustrate that reduced BCS ratios are a common feature of multiband superconductivity arising from interband coupling and band-dependent pairing strengths.

Whether the relatively small ratios carry additional physical significance beyond experimental uncertainty is vague, given the present measurements. Addressing this question would require more precise and carefully controlled experiments. Nevertheless, the observed values are compatible with conventional BCS limits when considering experimental uncertainties and the broad

superconducting transition in $MoTe_2$.

- **Results, page 3, line 9:**
  The individual gap contributions are displayed in Fig. 2(b), and the extracted gap values (symbols) are summarized in Fig. 2(c). Notably, the smaller gap exhibits a pronounced tail as T approaches T_c, consistent with finite coupling in a coupled two-gap system [Sec. S2]. This characteristic is shown for the BTK analysis (symbols) as well as the theoretical calculations (solid lines) and does not exist when there is no interband coupling (dashed lines), within a type-II Weyl-semimetal-derived SC model. The resulting BCS gap ratios are comparable to those reported for other multi-gap superconductors [Sec. S1.2.6]

[R1] H. Suhl, B. T. Matthias, and L. R. Walker, Bardeen-Cooper-Schrieffer theory of superconductivity in the case of overlapping bands, Phys. Rev. Lett. 3, 552 (1959)
[R2] S. Lee et al., Origin of extremely large magnetoresistance in the candidate type-II Weyl semimetal $MoTe_2$, Scientic reports 8, 13937 (2018)
[R3]M. M. Piva, L. O. Kutelak, R. Borth, Y. Liu, C. Petrovic, R. D. dos Reis, and M. Nickla,Superconducting pairing symmetry in $MoTe_2$, Phys. Rev. Materials. 7, L111801 (2023)
[R4] M. Tinkham, Introduction to Superconductivity, 2nd edition.
[R5] B. L. Blackford, A tunneling investigation of energy-gap anisotropy in superconducting bulk aluminum crystals, Journal of Low Temperature Physics, 23, 43 (1976)
[R6] E. Park et al., Spectroscopic evidence for two-gap superconductivity in the quasi-1D chalcogenide $Nb_2Pd_{0.81}S_5$, J. Phys.: Condens. Matter 30, 165401 (2018)
[R7] S. Ding et al., Surface structure and multi-gap superconductivity of V_3Si (111) revealed by scanning tunneling microscopy, Quantum Frontier, 2, 3 (2023)
[R8] R. Escudero and R.E. Lo'pez-Romero, The energy gap of the compound $FeSe_{0.5}Te_{0.5}$ determined by specific heat and Point Contact Spectroscopy, Solid State Communications 220, 21 (2015).
[R9] Y. De Wilde et al., Scanning Tunneling Microscopy Observation of a Square Abrikosov Lattice in $LuNi_2B_2C$, Phys. Rev. Lett., 78, 22 (1997).
[R10] X. Zhang et al., Evidence of a universal and isotropic $2\Delta/k_BT_c$ ratio in 122-type iron pnictide superconductors over a wide doping range, Phys. Rev. B, 82, 020515(R) (2010).

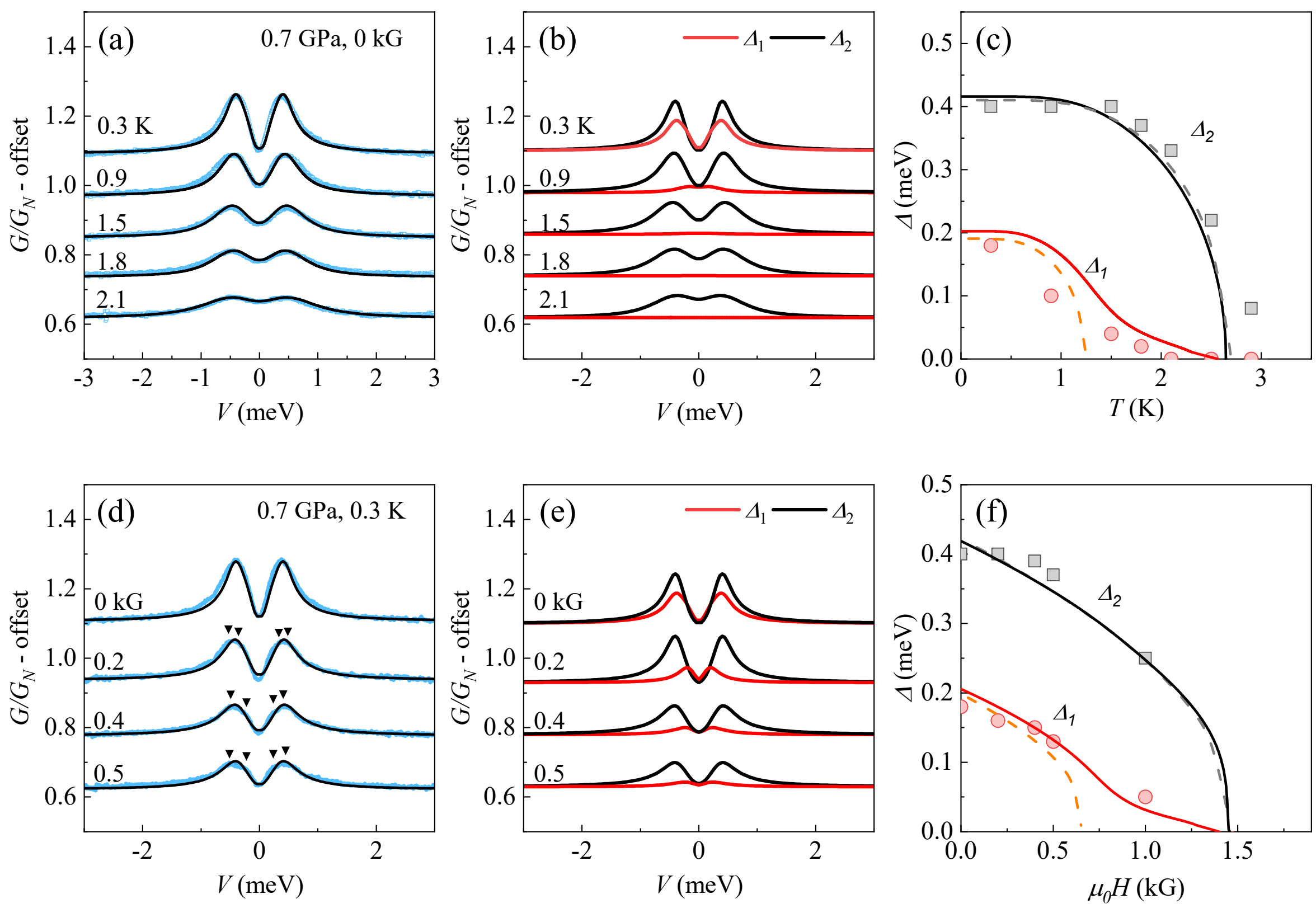

Fig. R1. **[Revised Fig. 2] Multi-gap superconductivity in T$_d$-MoTe$_2$ at 0.7 GPa.** **(a)** Temperature-dependent normalized conductance spectra. Symbols show experimental data, and solid lines represent two-gap s-wave BTK fits. **(b)** Decomposition of the BTK fit into contributions from the larger gap Δ_2 (black) and the smaller gap Δ_1 (red). Curves in (a,b) are vertically offset for clarity. **(c)** Temperature dependence of the extracted gaps $\Delta_{1,2}(T)$ from BTK analysis (symbols) compared with coupled-gap calculations. Solid lines include finite interband coupling, whereas dashed lines show the decoupled limit without coupling. **(d)** Field-dependent normalized conductance spectra at 0.7 GPa and 0.3 K with corresponding BTK fits (solid lines). Arrows mark the two gap-edge features and their evolution with the magnetic field. **(e)** Decomposition of the field-dependent spectra into Δ_2 (black) and Δ_1 (red) components. Curves in (d,e) are vertically offset for clarity. **(f)** Magnetic-field dependence of the gaps $\Delta_{1,2}(H)$ extracted from BTK analysis (symbols), compared with calculations with (solid lines) and without (dashed lines) coupling.

I have acknowledged that the spectra in a magnetic field indicate the presence of two gaps, whereas the zero-field data do not. However, this discrepancy still requires discussion. For instance, a similar phenomenon was observed in MgB_2, where, under dominant c-axis tunneling, only a small gap on the π-band was detected in zero field. However, under a magnetic field, this small gap was rapidly suppressed, revealing a larger gap with a small weight that persisted up to a higher upper critical field (Physica C 385 (2003) 244).

We thank the Referee for this thoughtful comment and for pointing out the analogy with MgB_2. The essence of the issue is that the multi-gap character is not very pronounced at zero field but becomes much clearer in a magnetic field. Below we summarize why this is expected in our case and how it is reflected in the revised Figs. 2(d-f).

1. Zero-field spectra: limited resolving power for two nearby gaps

At 0.7 GPa and zero field, the PCAR spectra [Fig. R1(a)] show two relatively broad conductance maxima. Because (i) the two gaps are comparable in magnitude and (ii) finite lifetime broadening causes their contributions to overlap, the overall lineshape can be reasonably captured by an effective single-gap BTK fit [Sec. S1.2.1]. This does not imply that superconductivity is single-gap, but only that the zero-field spectra have limited resolving power for two nearby gaps. When a two-gap BTK analysis is applied, it consistently yields two energy gaps of $\Delta_1 = 0.18$ meV and $\Delta_2 = 0.4$ meV, which satisfies the two-gap BCS criteria as discussed in the previous reply [revised Fig. R1(c) and Sec. S1.2].

2. Field dependence: two gaps and interband coupling

In a magnetic field, the situation changes qualitatively [Figs. R1(d-f)]:

- The field-dependent spectra at 0.3 K [Fig. R1(d)] cannot be described satisfactorily by a single-gap BTK model. Two-gap fits, by contrast, account for all fields with a single parameter set [Table I]. The decomposition [Fig. R1(e)] shows that the smaller gap loses spectral weight more rapidly at low fields, making the two-gap structure more visible than at zero field.

- The extracted gap amplitudes $\Delta_{1,2}(H)$ are plotted in Fig. R1(f). For comparison we indicate two limiting behaviours, corresponding to (i) nearly uncoupled gaps with different intrinsic H_{c2} and (ii) interband-coupled gaps with a common H_{c2}. Our data follow the coupled scenario. Although Δ_1 is suppressed more rapidly at low fields, it does not vanish at a separate low H_{c2}, but persists as a shoulder-like feature up to fields where Δ_2 is still present. This "long-tail" behaviour is difficult to reconcile with two independent gaps.

- Solid and dashed lines in Fig. R1(f) show the corresponding calculations within a two-gap superconducting model derived from the type-II Weyl-semimetal band structure [Supplementary Sec. S2]. With finite interband pairing, the theory reproduces the key features. As shown in Fig. R1(f), a smoother suppression of the larger gap and a smaller gap that is strongly reduced at low fields but survives as a tail up to the common H_{c2}. If the interband coupling is switched off, the small gap collapses at a much lower field, in clear disagreement with experiment, fully consistent with standard multiband BCS theory for overlapping bands [R1].

3. Relation to MgB_2

The Referee's example of MgB_2 [Physica C **385**, 244 (2003)] illustrates a similar qualitative ef-

fect. Under dominant c-axis tunnelling, the zero-field spectra mainly probe the small π-band gap, whereas a magnetic field suppresses this contribution and reveals the larger σ-band gap with a different H_{c2} [R11]. In T$_d$-MoTe$_2$, spectral broadening makes the zero-field spectrum compatible with an effective single-gap fit, but applying a field changes the balance between the two bands. Their distinct field responses, together with finite interband coupling, cause the two-gap structure to become pronounced in Figs. R1(d,e), while the $\Delta(H)$ behaviour in Fig. R1(f) shows that the smaller gap is "dragged" towards the common H_{c2} rather than closing independently.

We have revised the main text around Figs. R1(d-f) and expanded Secs. S1.2.6 and S2 to make this discussion explicit. We hope this clarifies why the two-gap nature is less evident in zero-field spectra but emerges in a magnetic field, in close analogy to the MgB_2 case, as pointed out by the Referee [R12].

- **Results, page 3, second paragraph:**
  The magnetic field dependence of the PCAR spectra further supports the multi-gap scenario. The two-gap character becomes apparent under fields up to 0.5 kG, as indicated by the arrows in Fig. 2(d), and the evolution of the two gaps with respect to a magnetic field is shown in Fig. 2(e). The extracted gap magnitudes $\Delta_i(H)$ from the BTK analysis are summarized in Fig. 2(f) (symbols). For comparison, the solid and dashed lines show calculations from multi-gap equations with and without interband coupling, respectively [Sec. S2], within the same model used in Figs. 2(c) Without interband coupling (dashed lines), the smaller gap is rapidly suppressed with increasing field and closes near its intrinsic pair-breaking scale. In contrast, finite interband coupling (solid lines) enforces a cooperative evolution of the two gaps, allowing the smaller gap to persist to higher fields by being sustained by the larger gap. This coupled-gap behavior reproduces the experimentally observed long-tailed suppression of the smaller gap as H goes to H_{c2}, which is absent in the decoupled limit.

- **Results, page 3, second to last sentence of third paragraph:**
  This behavior persists at an elevated pressure of 1.2 GPa as shown in Fig. S3, underlining the robustness of the two-gap structure against pressure [see Secs. S1.2.4-S1.2.6 for detailed data, fitting parameter, and field dependence].

[R11] S. Tajima *et al.*, "Superconducting gap in the multi-band system MgB_2," J. Low Temp. Phys. **131**, 1217 (2003).

[R12] P. Samuely *et al.*, "Point-contact spectroscopy of MgB_2," Physica: Superconductivity **385**, 244 (2003).

Figure S3 presents the spectra at 1.2 GPa with two-gap fits at different temperatures, but neither the data nor the fits are tabulated or compared with the gaps at 0.7 GPa. This comparison is necessary.

We thank the Referee for pointing out the need for an explicit comparison between the 0.7 GPa and 1.2 GPa data. In the revised Supplementary Information, we now provide a direct, side-by-side comparison of the two-gap analysis in the T_d phase [see Sec. S1.2.4 and S1.2.5]:

- **Revised Fig. S3** shows the PCAR spectra and two-gap s-wave BTK fits at 1.2 GPa as a function of temperature and magnetic field, displayed alongside the corresponding results at 0.7 GPa.
- **Revised Tables S4 and S5** list the temperature- and field-dependent BTK fitting parameters at 1.2 GPa, in the same format as Tables S2 and S3 for 0.7 GPa (including Z, w, $\Delta_{s,\text{large}}$, $\Delta_{s,\text{small}}$, and Γ).

From this comparison we find that:

- Pronounced double-peak structures persist at 1.2 GPa up to $T_c \approx 3.8$ K, and the temperature evolution of the large and small gaps closely follows that at 0.7 GPa. A finite interband coupling is again required to capture the extended tail of the smaller gap [Fig. S3(d), Secs. S1.2.4-S1.2.5].
- The magnetic-field dependence at 1.2 GPa exhibits the same two-step suppression of the conductance peaks as at 0.7 GPa, and the data are better described by a fit with a single common H_{c2} for the two gaps than by a fit with separate H_{c2} values [Figs. S3(b,c,e), Table S5].

These additions show that the two-gap superconducting state in the T_d phase of $MoTe_2$ is robust over a finite pressure range, rather than being specific to a single pressure point.

We have also added a brief discussion of this comparison to the main text to elucidate the consistency of the two-gap behavior across these pressures.

- **Results, page 3, second to last sentence of third paragraph:**
  This behavior persists at an elevated pressure of 1.2 GPa as shown in Fig. S3, underlining the robustness of the two-gap structure against pressure [see Secs. S1.2.4-S1.2.6 for detailed data, fitting parameter, and field dependence].

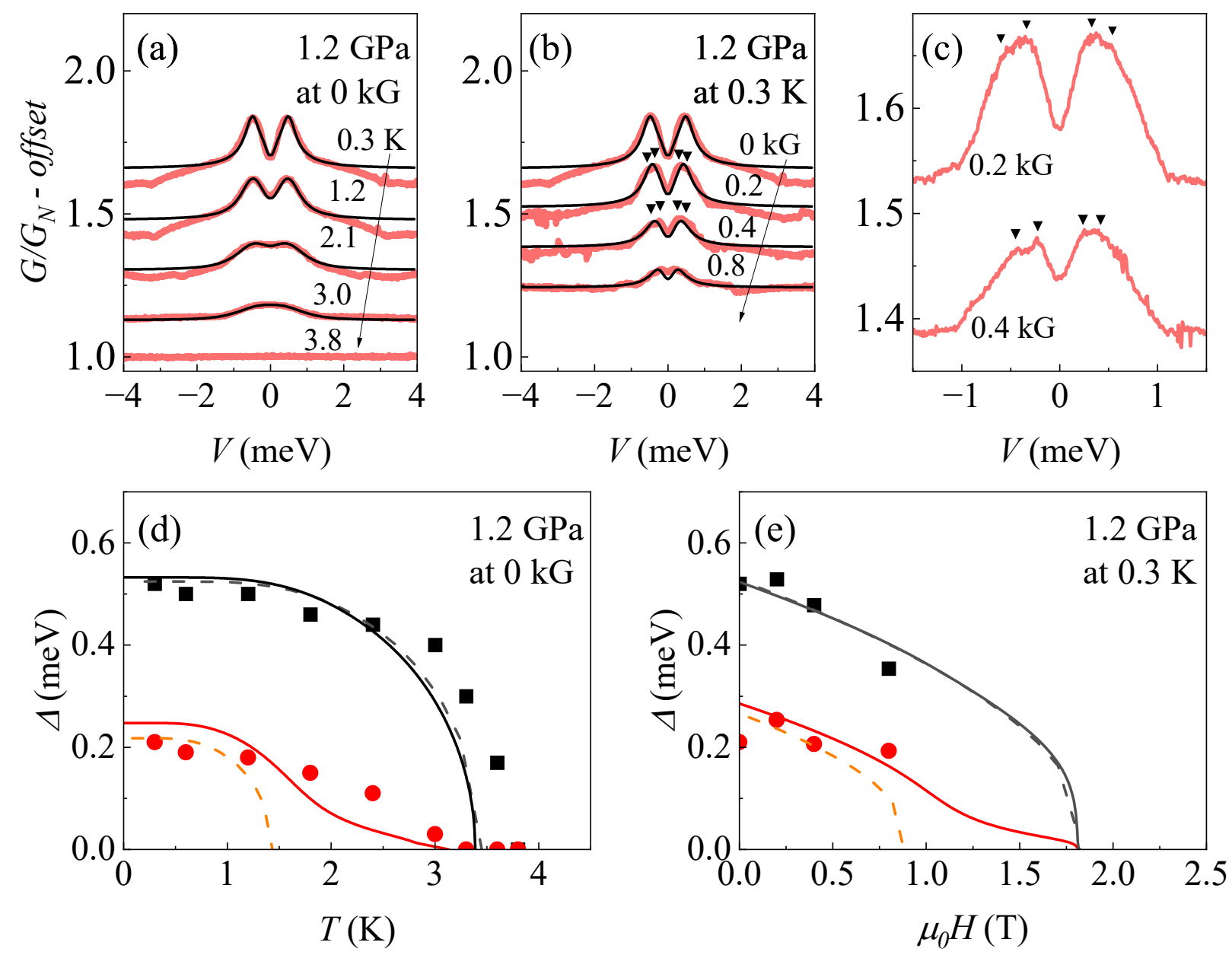

Fig. R2. [Revised Fig. S3] Alternative experimental data for $MoTe_2$ in the T_d phase at 1.2 GPa. **(a)** Temperature evolution of the normalized differential conductance, referenced to the 3.8 K data. Black curves are best fits using a two-gap s-wave BTK model; spectra are vertically offset for clarity with respect to the 0.3 K, 0 kG curve. **(b)** Magnetic-field evolution of the normalized differential conductance at 0.3 K. The two gap-edge features in the conductance peaks, indicated by black arrowheads, reflect the distinct field responses of the large and small gaps; curves are vertically shifted for clarity. **(c)** Magnetic-field evolution of the normalized differential conductance at 0.3 K, focusing on the two-gap feature (black arrows), is shown for 0.2 kG and 0.4 kG. **(d)** Temperature dependence of the two superconducting gaps. Solid and dashed lines represent theoretical calculations based on a coupled multi-gap model for a type-II Weyl-semimetal-derived superconducting system. The pronounced tail of the smaller gap persisting up to T_c arises from finite interband coupling between the two superconducting bands. **(e)** Magnetic-field dependence of the two superconducting gaps. The gap amplitudes follow closely the theoretical calculations based on the multi-gap model for a type-II Weyl-semimetal-derived superconducting system.

Additionally, there is an incorrect statement in Section II, Results: "These results are consistent with the previously reported topologically nontrivial superconductivity under ambient pressure [Refs. 30,31]." However, Ref. 31 presents only single-gap spectra with $2\Delta/k_B T_c \sim$ 3.7, and Ref. 30 explicitly states that the μSR experiment lacks phase/sign-of-the-gap resolution, merely speculating about topological issues based on sensitivity to disorder. Therefore, the authors should be more cautious when making claims about topologically nontrivial superconductivity.

We thank the Referee for correcting this point.

Per recommendation, we have rewritten the sentence to cite only the established result of ambient-pressure two-gap s-wave superconductivity in T_d-MoTe$_2$ (now Ref. [28]), and we have removed Ref. [31] entirely. The revised text now avoids any implication that prior work demonstrated topological superconductivity, and instead places our results solely in the context of multi-gap behaviour under pressure:

- **Results, page 3, end of third paragraph:**
  Taken together, these observations are consistent with previous muon-spin-rotation measurements at ambient pressure, which reported two s-wave gaps in T_d-MoTe$_2$ [28].

On superconductivity in monoclinic 1T′ phase

At the pressure above 1.4 GPa in the spectrum a central peak emerges in the PCAR spectra. In the revised manuscript, the authors perform a thorough analysis of the possible origins of the zero-bias conductance peak and conclude that s+p-wave gap is the most probable explanation for the spectra taken at 2.2. GPa. At the same time, they show that p-wave component is not intrinsic but rather induced from bulk to surface as evidenced by the similar temperature and field behavior of the s and p components.

Fig. 3(d) presents the spectrum after subtracting the s-wave contribution, but the resulting p-wave component appears very different from that shown in Fig. 2(c).

In the previous version of the manuscript, the two panels indeed represented different quantities: previous Fig. 2(c) showed the theoretical p-wave contribution obtained from the full s+p-wave BTK fit, whereas previous Fig. 3(d) displayed the experimental spectra after subtracting only the fitted s-wave component defined in previous Fig. 3(b). Because one panel mixed theory and the other showed a partial experimental subtraction, their lineshapes could appear inconsistent.

To remove this ambiguity, we have reorganized the figures and now present the s+p decomposition in a uniform way:

- The decomposition of the 2.2 GPa spectrum at 0.3 K and 0 kG into bulk s-wave and induced p-wave contributions is now shown consistently in revised Fig. 3(b), where both components come from a single self-consistent BTK fit rather than from ad hoc subtraction.
- All systematic measurements in the 1T′ phase are consolidated in the new Fig. 4. Panels in Figs. 4(a,d) present the experimental PCAR spectra as functions of temperature and magnetic field, respectively, while Figs. 4(b,e) show the corresponding s- and p-wave contributions extracted from the same BTK fits, making clear how each component shapes the total lineshape. The associated gap amplitudes and their evolution with field and temperature are compared with the theoretical gap evolution calculated from our microscopic model [Figs. 4(c,f)], supporting that the p-wave component is proximity-induced.

We believe that this restructuring makes the distinction between the measured spectra and the individual s- and p-wave contributions transparent and resolves the apparent discrepancy appeared in the previous version.

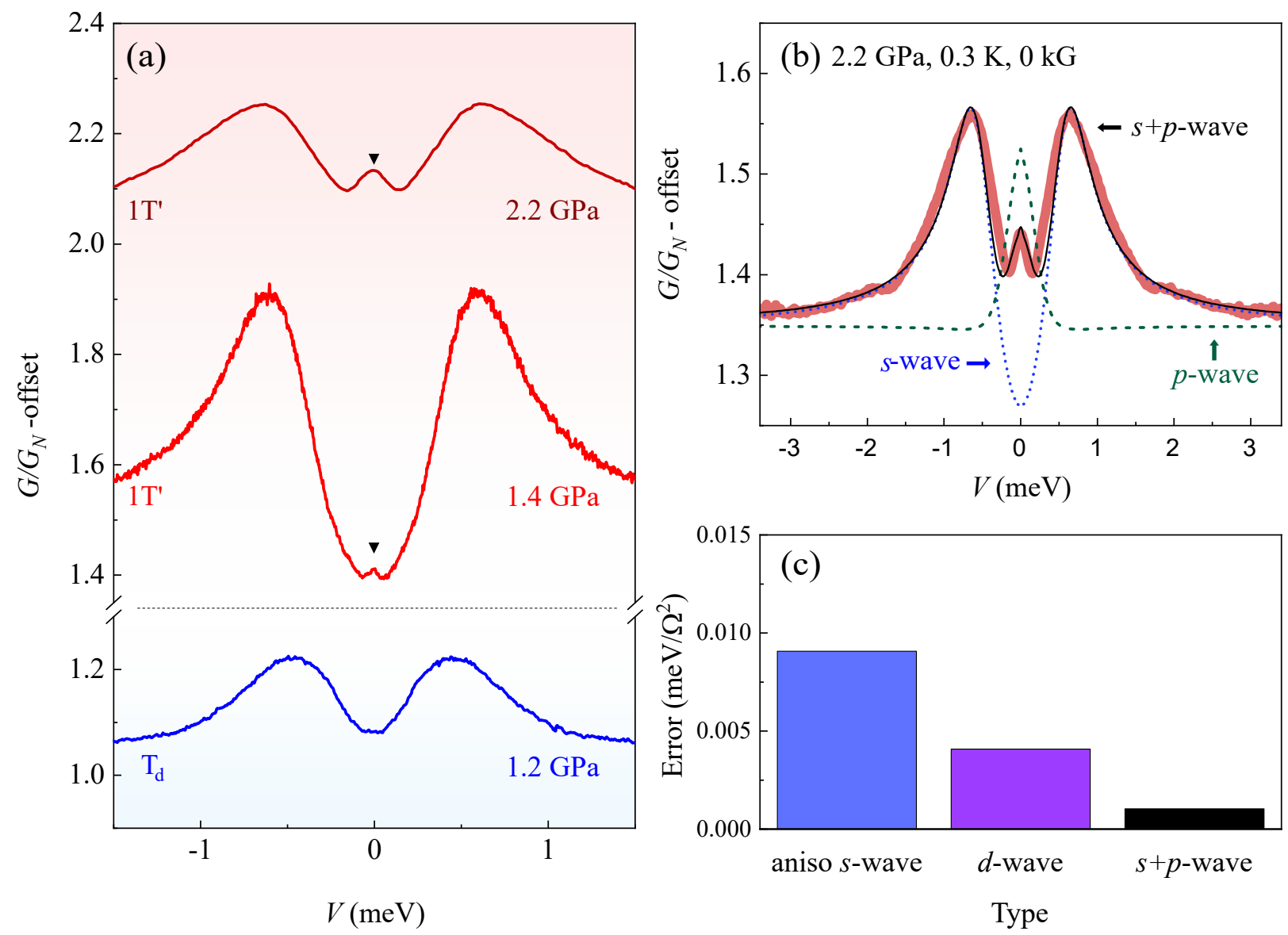

Fig. R3. **[Revised Fig. 3] Pressure-tuned emergence of $s+p$-wave superconductivity in $MoTe_2$. (a)** Evolution of the normalized differential conductance from the T_d (1.2 GPa) to the 1T′ (1.4 and 2.2 GPa) phases at 0.3 K. The black triangle marks a conductance peak near zero-bias, which appears only in the 1T′ phase. **(b)** BTK fits to the normalized conductance measured at 2.2 GPa. Blue, green, and black curves represent the s-, p-, and combined $s+p$-wave SC states, respectively, derived from two-gap BTK modeling. **(c)** Total fitting error, estimated as the difference between the experimental data and BTK calculations, which highlights the agreement of the $s+p$-wave model.

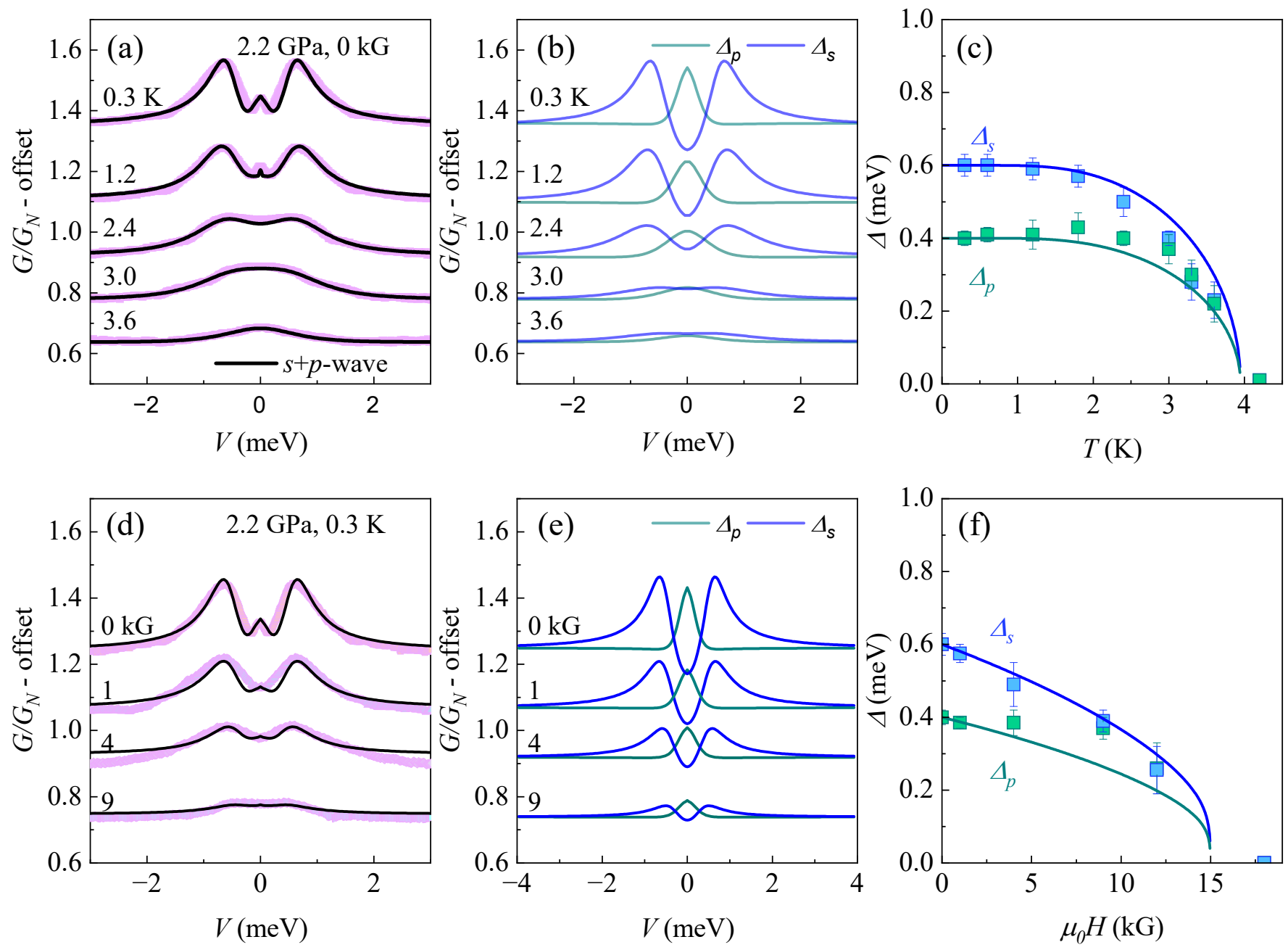

Fig. R4. **[Revised Fig. 4] Surface s+p-wave superconductivity in 1T′-MoTe$_2$ at 2.2 GPa. (a)** Temperature-dependent normalized differential conductance spectra of MoTe$_2$ at 2.2 GPa. Colored curves show experimental data, while black curves represent extended BTK fits using a s+p-wave model. **(b)** Decomposition of the BTK fits in (a) into the s-wave (black) and p-wave (red) components. Curves in (a,b) are vertically offset for clarity. **(c)** Temperature dependence of the extracted gaps $\Delta_s(T)$ and $\Delta_p(T)$ (symbols) compared with theoretical calculations including interband coupling (solid lines). **(d)** Field-dependent normalized conductance spectra at 0.3 K (symbols) with BTK fits (solid lines), showing a progressive suppression of the single peak near zero-bias without observable splitting. **(e)** Decomposition of the field-dependent spectra in (d) into the s- and p-wave components. Curves in (d,e) are vertically offset for clarity. **(f)** Magnetic-field dependence of $\Delta_s(H)$ and $\Delta_p(H)$ (symbols), compared with theoretical calculations (solid lines).

Also question arises what happened to the two s-wave gaps observed in the orthorhombic T_d phase?

In our picture, both superconducting gaps in T_d-$MoTe_2$ arise from distinct normal-state bands that descend from the high-symmetry T_0 structure. As established by previous first-principles and band-structure studies [R1-R3], the centrosymmetric T_0 phase hosts Dirac-like bands. Breaking inversion symmetry stabilizes a type-II Weyl semimetal in the T_d phase, while an in-plane shear distortion drives the system into the 1T′ phase, where the Dirac/Weyl cones are gapped into a second-order topological insulator (SOTI) band structure. Our schematic phase evolution in Figs. R5(b,d) follows this established literature.

Within this framework, the two-gap s-wave behaviour observed in the T_d phase at 0.7 and 1.2 GPa [Figs. R1, R2, and Supplementary Sec. S2] is naturally described by a two-band BCS model in which both the Weyl-derived band and a parabolic band crossing the Fermi level near Γ contribute to superconductivity. Upon further increasing pressure, the structural transition to the 1T′ phase gaps out the Weyl cone and converts it into topological surface (and hinge) states of the SOTI band, while only the parabolic band remains as a bulk Fermi surface [schematically illustrated in Fig. R5(d)]. As a result, the bulk superconductivity in 1T′-$MoTe_2$ is characterized by a single s-wave gap on this remaining band, consistent with our BTK analysis of the 1T′ spectra. At the same time, the SOTI surface/hinge states inherit a proximity-induced s+p-wave gap from the bulk s-wave condensate, which is what gives rise to the surface s+p channel resolved in our high-pressure PCAR data [Figs. R3 and R4].

In this way, the two-gap s-wave state in the T_d phase evolves consistently with the known T_0 to T_d/1T′ band-structure evolution. One band continues as the bulk s-wave superconductor in the 1T′ phase, while the other is transformed into higher-order topological surface states that host the proximity-induced s+p pairing component.

The corresponding changes in the manuscript are as below.

- **Results, page 6 first and second paragraph:**
We now theoretically investigate the SC gaps of the T_d and 1T′ phases below T_c. A high-symmetry parent phase, T_0, has been proposed to evolve into the noncentrosymmetric T_d phase via inversion-symmetry breaking, or into the centrosymmetric 1T′ phase via a shear-mode distortion [see Secs. S2.1 and S3.1 for model Hamiltonians]. In the T_0 phase, the low-energy band structure near the Γ point consists of a parabolic band and a Dirac-like cone, with PT symmetry constraining the degeneracies. When inversion symmetry is broken and the system enters the T_d phase, the Dirac-like cone splits into type-II Weyl cones located near the Fermi level, which coexist with the parabolic band [Fig. 1(b)]. Based on this electronic structure, we construct a SC Hamiltonian to rationalize the observed gap structure, in which both the parabolic band and the Weyl sectors acquire gaps below T_c. We derive coupled two-gap BCS self-consistency equations from the mean-field theory and solve them numerically; a finite interband coupling is essential to reproduce the experimentally observed persistence of the smaller gap toward T_c [see Method and Sec. S2 for detailed analysis]. In the following, we contrast this multi-gap pairing framework in the T_d phase with the centrosymmetric 1T′ phase, where the shear-distorted band topology gives rise to higher-order (hinge) boundary states that can host a proximity-induced p-wave component.

To describe the 1T′ phase, we adopt a minimal model capturing the low-energy effective theory in which the effect of the shear distortion is encoded through SOC and symmetry-allowed mass terms. The SOC gaps out the Dirac-like cone of the T_0 phase, and the additional mass terms drive a double band inversion, realizing an SOTI band, as illustrated in Fig. 1(d). As shown in Fig. 5(a), this SOTI band structure supports surface states that consist of two helical Dirac cones with spin-momentum locking, which are gapped by symmetry-allowed mass terms [see Sec. S3.1.3 for analytical details]. In a finite geometry, these surface Dirac cones, in turn, host one-dimensional gapless hinge states, a hallmark of higher-order topology [Fig. 5(b)]. Additional details—including the lattice Hamiltonian, symmetry analysis, and numerical and analytical results—are provided in Sec. S3.1.

We updated Figs. 1 and 5 of the main text (Figs. R5 and R6, respectively) to better illustrate the changes in the system across the phase transition.

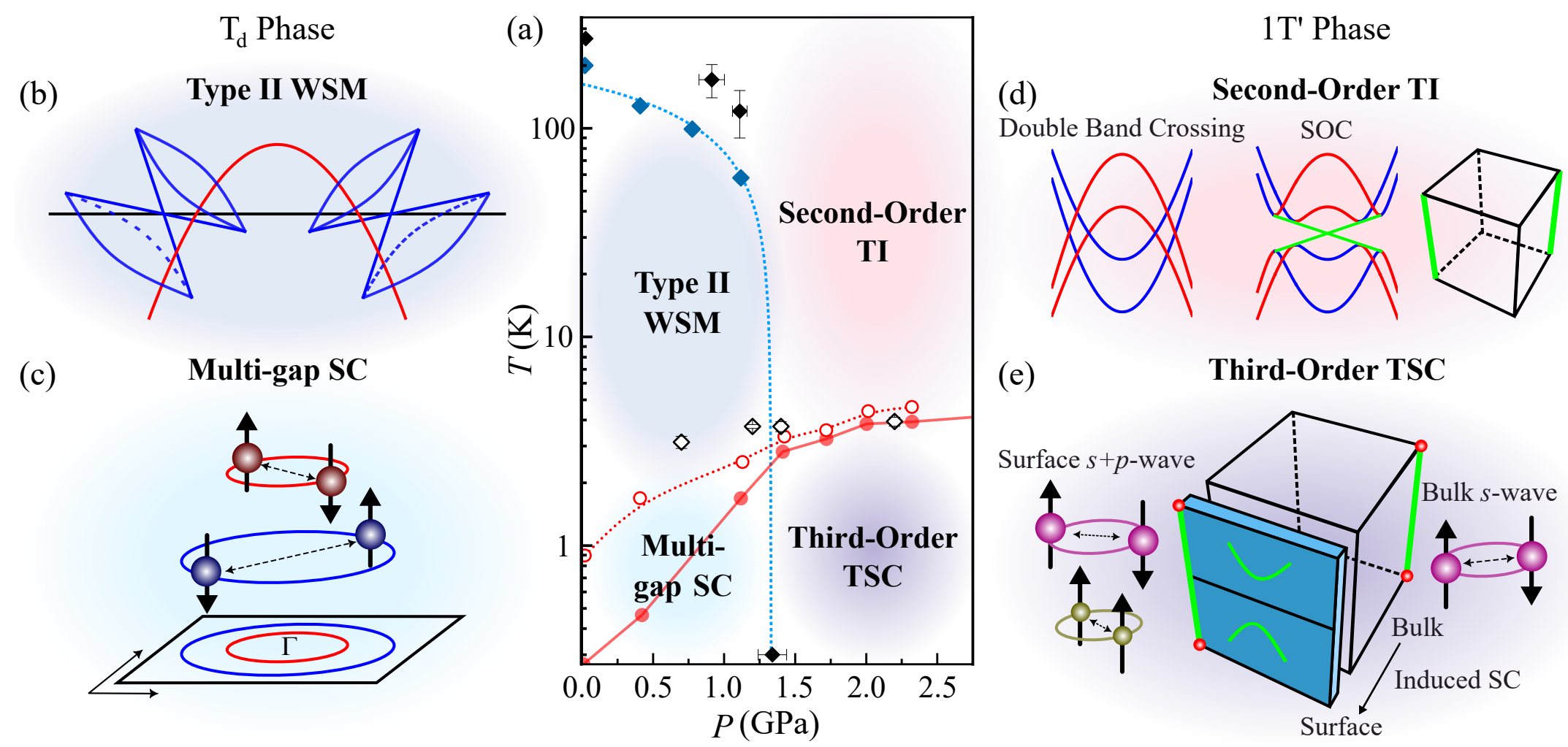

Fig. R5. **[Revised Fig. 1] Temperature-pressure phase diagram and schematic evolution of topology and superconductivity in $MoTe_2$. (a)** Phase diagram of $MoTe_2$ across the T_d and 1T′ phases. Blue and black diamonds indicate the structural transition temperature (T^*) from 1T′ to T_d derived from resistivity and X-ray data. Red circles mark the onset ($T_{c,onset}$) and zero-resistance (T_{zero}) SC transitions, while open black diamonds show T_c measured by point-contact Andreev reflection, in good agreement with $T_{c,onset}$. The suppression of T^* correlates with a sharp rise of T_c in the T_d phase near the putative quantum critical point ($P_c \approx 1.4$ GPa). **(b)** Schematic band structure near Γ for the type-II Weyl semimetal, where a parabolic band (red) lies between two Weyl cones (blue). **(c)** Multi-gap superconductivity in the T_d phase. Below T_c, both the parabolic band and Weyl cones in (b) develop distinct SC gaps, forming separate Cooper-pair condensates. **(d)** Band structure and wave-function distribution of the second-order topological insulating band. With SOC, the double band crossing (left) opens a gap, yielding a bulk insulator that hosts second-order topological boundary states (green, middle), localized at the crystal hinges or corners (right). **(e)** Schematic of the bulk-to-surface proximity effect. Bulk s-wave superconductivity induces a p-wave component on the helical surface states (left), forming s+p-wave pairing on the surface (right) and gapped hinge states (green) hosting localized Majorana corner modes (red) in the proposed third-order topological superconducting phase.

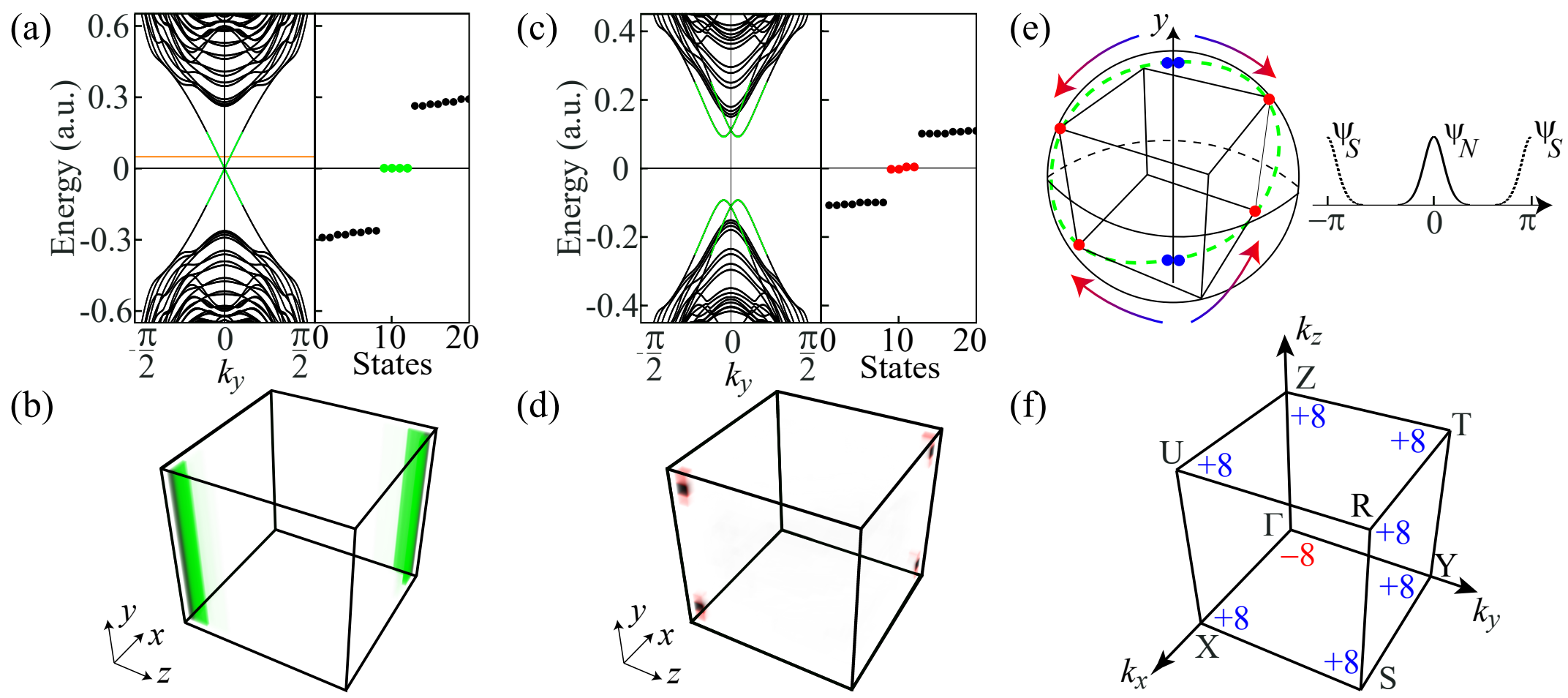

Fig. R6. **[Revised Fig. 5] Theoretical analysis of the emergence of Majorana corner states. (a)** (Left) Band structures of the finite tight-binding Hamiltonian with 15×15 unit cells in the xz plane and periodic in y (black) for the 1T′ phase. The analytic hinge dispersion (green) overlaps with the numerical result; the orange line marks $\mu = 0.05$. (Right) Energy eigenvalues at $k_y = 0$. **(b)** Wavefunction distributions of zero-energy hinge states. Darker color indicates higher density. **(c,d)** (Left) Band structures for the SC phase with 15×15 unit cells (black), where the analytic hinge dispersion (green) coincides with the numerical result. (Right) Eigenvalues of the $15 \times 15 \times 15$ lattice showing four nearly zero-energy states, whose real-space wavefunction distributions are shown in (d). **(e)** (Left) Schematics of Majorana zero-energy states; pole states (blue) in spherical geometry move to corner states (red) along the dotted green line in cubic geometry. (Right) Localized Majorana wavefunctions along a hinge through the poles, with north (Ψ_N, solid) and south (Ψ_S, dashed) states well-localized at $\theta = 0$ and π. **(f)** Parity eigenvalues of occupied states at TRIM points. The parameters are given in Methods.

On page 8 it is stated "However, an anisotropic s-wave scenario is improper due to the ill-fitting, as shown in Fig. S4(a).". However, it appears that Fig. S4(a) actually uses an isotropic s-wave gap, not anisotropic one.

We apologize for the confusion caused by the incorrect figure reference in the Supplementary Information.

To improve clarity and consistency, we have reorganized the presentation of the fitting results: the isotropic s-wave and p-wave fits in Fig. S5 and the anisotropic s-wave and d-wave fits now appear in Fig. S6, given below.

Accordingly, the anisotropic s-wave fit, which was originally intended to be referenced, is now clearly shown in Figs. S6(a,b), consistent with the statement in the main text.

The figure reference in the main text has also been updated from Fig. S4(a) to Figs. S6(a,b).

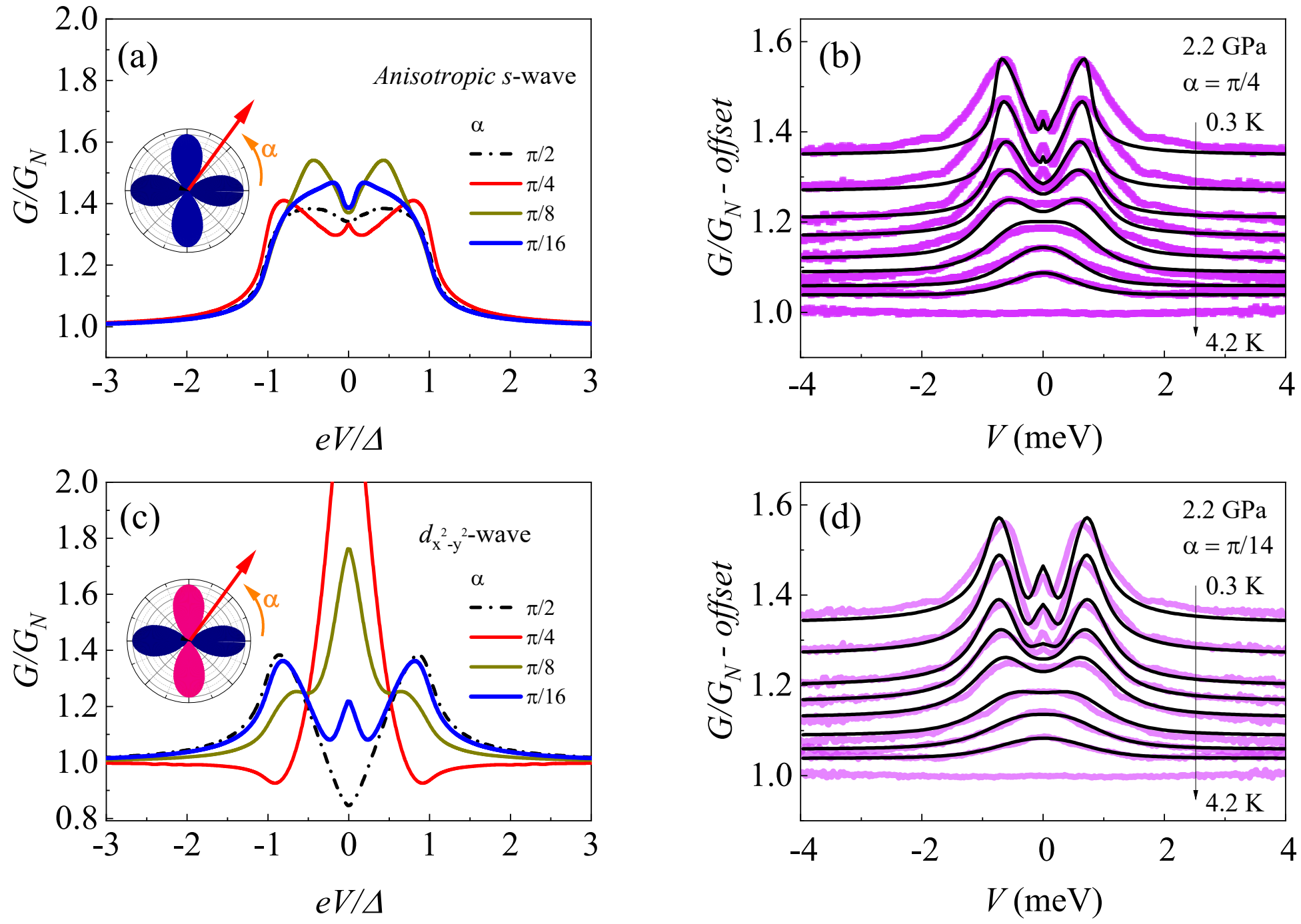

Fig. R7. [Revised Fig. S6] Blonder-Tinkham-Klapwijk (BTK) analysis of competing gap symmetries in the 1T′ phase. **(a,c)** Simulated PCAR spectra for (a) anisotropic s-wave and (c) $d_{x^2-y^2}$-wave pairing states, calculated for several polar tunneling angles $\alpha = \pi/2, \pi/4, \pi/8$, and $\pi/16$. The angle α denotes the orientation of the soft point contact relative to the gap structure. In (a), $Z = 0.42$ and $\Gamma = 0$ meV, while in (c), $Z = 0.69$ and $\Gamma = 0.13$ meV. **(b,d)** Temperature-dependent PCAR spectra at 2.2 GPa (colored curves) fitted by (b) anisotropic s-wave and (d) $d_{x^2-y^2}$-wave BTK models. In (b) and (d), the representative tunneling angles are $\alpha = \pi/4$ and $\alpha = \pi/14$, respectively. For clarity, all spectra are vertically offset relative to the 0.3 K curve.

Additionally, Figures 2 and S5 are identical.

As you noted, Figs. 2 and S5 in the previous version present the same data set.

In that version, Fig. S5 was included in the Supplementary Information to show the corresponding fitting parameters (Table S4 of the previous manuscript), and provide additional details such as the barrier strength Z_i, which are not shown in the main text.

In the revised version, to avoid confusion and improve clarity, Fig. S5 has been removed from the Supplementary Information and the layout has been revised accordingly.

Response to Referee #3

The authors report on high-pressure point-contact Andreev reflection (PCAR) spectroscopy measurements in $MoTe_2$, aiming to uncover unconventional superconductivity and possible higher-order topological phases. In particular, they claim the observation of two-gap s-wave superconductivity in the T_d phase and proximity-induced surface p-wave superconductivity in the high-pressure $1T'$ phase, further supported by theoretical modeling suggesting the emergence of third-order topological superconductivity (TOTSC) and associated Majorana hinge modes. The manuscript addresses an important and timely topic at the interface of superconductivity and topological materials. The experimental technique, soft point-contact spectroscopy under quasi-hydrostatic pressure, is technically demanding and well-suited for exploring pressure-induced phases. However, I have several concerns regarding the robustness of the conclusions and the strength of the evidence presented.

We thank the Referee for the careful summary of our work and for recognizing both the topical relevance of pressure-tuned superconductivity in $MoTe_2$ and the technical challenges inherent to high-pressure PCAR measurements. In order to address the Referee's concerns regarding the robustness of our conclusions and the strength of the supporting evidence, we have re-analyzed the data, expanded the experimental basis, and sharpened the theory-experiment connection, as outlined below.

- **Strengthened the discussion on two-gap superconductivity in the T_d phase.**
  We now provide an expanded set of temperature- and field-dependent PCAR spectra at 0.7 GPa, together with a systematic two-gap BTK analysis and two-band BCS modelling including interband coupling [revised Fig. 2 and Sec. S1]. Additional PCAR measurements at 1.4 GPa, compared with the data at 0.7 GPa, confirm that the multi-gap superconducting behavior is reproducible [Fig. S3 and Sec. S1.2.4-S1.2.5].

- **Clarified the fitting strategy and model selection.**
  We have reorganized the description of our fitting procedures to clearly distinguish between single-gap, two-gap, and anisotropic-gap scenarios. For each pressure regime, we now show side-by-side comparisons of these models, summarize fit quality metrics, and provide complete tables of fitting parameters, allowing the reader to assess the degree to which the data require a multi-band and/or anisotropic description [revised Secs. S1.2-S1.3 and Tables S1-S7].

- **Refined the analysis of the $1T'$ phase and the interpretation of the zero-bias conductance peak.**
  Additional field- and temperature-dependent spectra at high pressure are included, together with a more detailed discussion that excludes alternative origins of the ZBCP including Andreev bound states and heating effects. We show that an s+p-wave BTK description, in which the surface p-wave component tracks the bulk s-wave gap, most consistently captures the observed behavior, while explicitly stating the assumptions and limitations of this interpretation [revised Fig. 3 and Sec. S1.3]. We also present experimental results for alternative conditions on another sample, to show the reproducibility of our findings [Fig. S9].

- **Higher-order topology: role of theory and candidate status.**
  The theoretical modeling of higher-order topology and possible third-order topological superconductivity has been streamlined and aligned more closely with the experimental

observables. The temperature and field dependence of the gaps in both phases is semi-quantitatively captured by a single microscopic framework [Figs. 2 and 4], which we now explicitly present as a consistent and predictive scheme for interpreting the data, rather than as a direct demonstration of hinge or corner-localized modes. We clearly distinguish experimental results (multi-gap and pressure-induced superconductivity) from theoretically motivated scenarios (Majorana corner states) and refer to the 1T′ phase as a candidate of higher-order topological superconductor.

- **Improved organization and transparency of the Supplementary Information.** All fitting parameters, model assumptions, and pressure-dependent trends are now consolidated in a small number of key figures and tables [revised Figs. S1-S4 and Tables S1-S7], making it easier to trace how each conclusion is supported by the underlying data and analysis.

We believe that these revisions substantially reinforce the experimental foundations of our main claims, clarify the level of certainty associated with the proposed topological interpretations, and address the concerns on the robustness of the evidence. A point-by-point response to the specific comments is provided below.

The main experimental claim of the manuscript is the observation of a zero-bias conductance peak (ZBCP) in the 1T′ phase, interpreted as evidence for surface p-wave superconductivity. While the BTK fits using an s+p-wave model can reproduce the ZBCP, the distinction from alternative multi-gap scenarios—such as two s-wave gaps with a very small Δ_2*—is not fully convincing. As Referee 2 also noted, ZBCPs can arise in multi-gap superconductors without requiring unconventional pairing. The authors argue that the observed ZBCP is inconsistent with such a scenario based on the gap-size ratios and their temperature dependence. While this interpretation is plausible, it would be significantly strengthened by additional diagnostics, such as angular conductance anisotropy or impurity sensitivity, to more rigorously constrain the pairing symmetry.*

We thank the Referee for raising this important point and for emphasizing the need to sharply distinguish our interpretation from conventional multi-gap scenarios.

In line with this concern, in the revised manuscript we avoid the term zero-bias conductance peak (ZBCP), which may suggest a specific topological interpretation, and instead refer to the observed feature as a single peak near zero-bias.

We then address the Referee's question on two levels: by performing a quantitative comparison of competing gap symmetries within a common BTK framework, and by explicitly testing whether a two-gap s-wave state or extrinsic mechanisms could account for the single near-zero-bias peak. The main points are as follows.

1. **BTK model selection: *s+p* versus competing gap structures.**
   We extend the BTK analysis of the 1T′-phase spectra at 2.2 GPa to single-gap s-wave, single-gap p-wave, anisotropic s-wave, d-wave, and mixed s+p-wave parameterizations [revised Fig. 3(c), Figs. S4-S6]. Using the same fitting protocol and error metric for all cases, the s+p-wave model yields the smallest residuals and alone reproduces both the sharp central peak and the flanking Andreev maxima. The optimized s+p fits reveal two comparable gap scales, with $\Delta_s \sim 0.6$ meV and $\Delta_p \sim 0.4$ meV [Figs. 3(b) and 4(c)], demonstrating that the p-wave component provides a substantial and robust contribution rather than arising from fine-tuned numerical adjustments.

2. **Explicit test of a two-gap *s+s* origin.**
   In Sec. S1.4.2 and Fig. S8 we systematically explore whether a two-gap s+s-wave state with a very small secondary gap can mimic the observed single peak near zero-bias. BTK simulations for two isotropic s-wave gaps, Δ_{large} and Δ_{small}, show that a pronounced single near-zero-bias peak occurs only under rather extreme conditions: an unusually large gap disparity, $\Delta_{\text{large}}/\Delta_{\text{small}} \gtrsim 7$, and temperatures close enough to T_c that thermal smearing essentially washes out the smaller-gap structure. For more moderate gap ratios, clear two-gap features remain. In our experiment at 2.2 GPa, the extracted gaps satisfy $\Delta_s/\Delta_p \sim 1.3$-$1.5$, and the key spectra are taken at $T \approx 0.3$ K $\ll T_c$ [Figs. 3(a,b) and 4(a,c)]. In this realistic parameter range, a purely two-gap s-wave model does not generate a sharp, unsplit peak comparable to our data, whereas the mixed s+p state does so consistently.

3. **Temperature/field evolution and pressure dependence.**
   The extended s+p BTK fits track the full temperature and field evolution of the 2.2 GPa spectra [Figs. 4(a,b,d,e), Sec. S1.3.4]. Both $\Delta_s(T,H)$ and $\Delta_p(T,H)$ decrease in tandem

and vanish at the same T_c and pair-breaking field, which is quantitatively reproduced by our microscopic model of a proximity-induced surface p-wave component strongly coupled to a bulk s-wave condensate [Figs. 4(c,f)]. Moreover, throughout the noncentrosymmetric T_d phase we observe robust two-gap s-wave superconductivity without any enhancement at zero bias [Fig. 2, Sec. S1.2]. The sharp single peak near zero-bias emerges only in the centrosymmetric 1T′ phase and follows the structural phase boundary [Fig. 3(a), Fig. S9], which is difficult to reconcile with a generic multi-gap s-wave mechanism already operative in T_d.

4. **Extrinsic mechanisms and outlook for further diagnostics.**
We have added Sec. S1.4.1 and Fig. S7 to demonstrate that point-contact heating produces a broad, strongly field-sensitive maximum at zero-bias, accompanied by a zero-bias conductance that follows the inverse temperature dependence of the bulk resistivity. This behavior stands in stark contrast to the sharp zero-bias peak observed in our ballistic contacts, which coexists with well-resolved Andreev-reflection features and does not exhibit the $G(0,T) \propto 1/\rho(T)$ scaling characteristic of the thermal regime. In Sec. S1.4.3 and Fig. S9 we argue against magnetic-impurity scenarios: impurity-induced bound states are expected to Zeeman-split under field, whereas our single near-zero-bias peak remains unsplit and is merely suppressed in amplitude [Figs. 4(d,e)], and it appears reproducibly only in the 1T′ phase. We fully agree that angle-resolved PCAR and controlled impurity studies would provide even more stringent constraints on the pairing symmetry. Such experiments are technically demanding at multi-GPa pressures, but we are actively developing angle-sensitive PCAR and complementary impurity-controlled measurements at ambient pressure for future work.

We have revised the relevant parts of the Results section and Supplementary Information to reflect this analysis and to clarify that our results are not consistent with a purely two-gap s-wave explanation, while supporting a proximity-induced surface $s{+}p$ state in the 1T′ phase within the limitations of the current experimental setup.

The corresponding changes in the manuscript are summarized below.
Specifically, we have revised the final paragraph on page 13 of the Results section to improve clarity and alignment with this discussion.

- **Results, page 5, second paragraph:**
Before turning to our theoretical analysis, we evaluate the origin of the single peak near zero-bias in Figs. 3 and 4 by excluding extrinsic mechanisms that could mimic a p-wave response. One possible scenario is a heating-induced thermal regime, in which local Joule heating and critical-current effects can generate a broad, strongly field-sensitive anomaly [Fig. S7]. However, the magnetic-field-dependent PCAR spectra at 2.2 GPa and 0.3 K shown in Fig. 4(d) exhibit none of these thermal signatures, and the zero-bias conductance does not follow the expected $G(0,T) \propto 1/\rho(T)$ scaling, arguing against a heating-induced origin [Sec. S1.4.1]. A second possibility is that a single peak near zero-bias arises within a two-gap s-wave scenario when one gap is extremely small. Such a scenario would require an extreme gap disparity—the larger gap must exceed the smaller one by at least a factor of seven at 3.3 K [see Fig. S8 and Sec. S1.4.2 for a detailed argument]. At 2.2 GPa, by contrast, the extracted p-wave gap is approximately 75% of the s-wave gap, well outside this

regime [Fig. 4(c)]. Given the small gap ratio and the low measurement temperature (0.3 K), a purely two-gap s-wave model cannot account for the observed zero-bias peak.

Furthermore, in Fig. 2(c), the authors apply a two-gap s+p-wave model, which introduces a large number of fitting parameters. This raises concerns about potential overfitting and reduces the credibility of the analysis. What are the fitting uncertainties? Are the fits robust under reasonable variation of parameters?

We thank the Referee for the constructive comments and for raising the important issue of possible overfitting in our two-gap s+p-wave analysis. In the revised manuscript we address (i) how the BTK model is parametrized and constrained, (ii) the associated fitting uncertainties, and (iii) the robustness of the results under parameter variations, as summarized below and discussed in detail in Supplementary Secs. S1.3 and S1.4.

(1) Parameterization and constraints
In the two-gap s+p-wave BTK fits for the 1T$'$ phase at 2.2 GPa the effective number of free parameters per spectrum is kept deliberately small:

- The tunnelling angle is fixed to $\alpha = 0$ (consistent with the contact geometry), and the relative spectral weight of the two components is fixed to $w = 0.5$.
- A single barrier parameter Z and a single broadening parameter Γ are used for a given contact and kept common to the entire temperature- or field-dependent data set.
- Only the two gap amplitudes, Δ_s and Δ_p, are allowed to vary with T and H, subject to a smooth evolution and a common T_c and pair-breaking field.

Thus, most parameters are shared across many spectra rather than adjusted independently, and the model complexity is constrained by physical considerations.

(2) Fitting uncertainties
We estimate the uncertainties by mapping the χ^2 landscape in the (Δ_s, Δ_p) plane and by varying Z and Γ within physically reasonable bounds. For each spectrum we identify the region where the normalized χ^2 increases by less than 5% above its minimum. This procedure yields typical relative uncertainties of order 5% in the extracted gap magnitudes, while the central values remain close to $\Delta_s \approx 0.6$ meV and Δ_p of comparable size [revised Figs. 3(b,c) and 4(c)]. The corresponding error bars and representative parameter sets are now explicitly shown in the revised figures and Tables S6 and S7.

(3) Robustness against parameter variation and model choice
The stability of the s+p-wave description is supported by several tests:

- Starting the optimization from widely different initial guesses and scanning a broad parameter range always yields convergent solutions with similar (Δ_s, Δ_p) within the above uncertainties.
- Varying Z and Γ within reasonable ranges modifies the detailed line shape but does not eliminate the need for a sizable p-wave component to reproduce both the single peak near zero-bias and the flanking Andreev maxima.
- A quantitative comparison with simpler models shows that isotropic s, anisotropic s, pure p, and d-wave fits all exhibit significantly larger residuals [Fig. 3(c), Fig. S4]. In particular, anisotropic s-wave and d-wave descriptions require finely tuned nonzero tunnelling angles ($\alpha \neq 0$) and still leave systematic discrepancies, whereas the constrained s+p model with $\alpha = 0$ provides the most consistent description across temperature and field.

In summary, although the two-gap $s{+}p$-wave BTK model formally contains more parameters than a single-gap description, most of these parameters are fixed or globally constrained, the resulting uncertainties in the extracted gaps are moderate and quantified, and the fits are robust against reasonable parameter variations. Together with the systematic failure of alternative gap symmetries to describe the data with fewer parameters, this supports our conclusion that the two-gap $s{+}p$-wave model provides the most reliable and physically consistent description of the spectra in the 1T$'$ phase.

Additionally, to dismiss other possible origins of the ZBCP, such as heating effects, the authors argue that "in this case, the ZBCP is generally broad and exhibits sensitivity to weak magnetic fields due to the critical current and high normal-state resistivity..." This statement is not sufficiently compelling.

We thank the Referee for pointing out that our original statement regarding heating effects was simplistic, and we agree that a more explicit justification is required to exclude a thermal origin of the single peak near zero bias. In the revised manuscript we now provide a detailed discussion in Supplementary Sec. S1.4.1 and an explicit experimental example [revised Fig. S7 below]. Here we summarize the main points:

- **Expected line shape in the thermal (non-spectroscopic) regime.** As discussed in Sec. S1.4.1, when the effective contact diameter exceeds the inelastic mean free path or the current density approaches the critical current density J_c, the junction crosses over from the ballistic/diffusive PCAR regime to a thermal regime. In this limit the injected power $P = IV$ is dissipated locally and the electronic temperature in the constriction rises well above the bath temperature. Self-heating and critical-current effects then dominate the differential conductance, which acquires a characteristic non-spectroscopic line shape. This behavior is characterized by a broad zero-bias conductance maximum with suppressed conductance at finite bias and an absence of sharp Andreev-reflection features, often accompanied by strong magnetic-field sensitivity and a zero-bias conductance that follows the inverse temperature dependence of the bulk resistivity. See Supplementary Sec. S1.4.1.
- **Experimental example of a thermal contact in $MoTe_2$.** Figure S7(a) (Fig. R8) shows a representative spectrum measured at $T \approx 0.3$ K in the thermal, non-spectroscopic regime. The conductance $G(V)$ exhibits a wide hump centered at zero bias, flanked by shallow minima at finite bias and lacking any well-defined gap-edge or Andreev-reflection features. This line shape is characteristic of a point contact dominated by local heating and critical-current effects, for which the conductance primarily reflects bulk transport properties rather than the superconducting quasiparticle spectrum (see Fig. S7(b)). It is therefore clearly distinct from the spectra used in the analysis of the pressure-induced superconducting gaps.
- **Contrast with the high-pressure PCAR spectra at 2.2 GPa.** In the 1T′ phase at 2.2 GPa and 0.3 K, the spectra analyzed in Figs. 3 and 4 display a sharp single peak near zero bias that coexists with well-resolved Andreev side peaks, and the conductance at finite bias is not suppressed. Moreover, the magnetic-field-dependent spectra of Fig. 4(d) do not show the broadening and strong field sensitivity characteristic of thermal contacts. The central peak remains narrow and unsplit and is only gradually reduced in amplitude over the entire field range. These features are incompatible with the signature of the thermal regime exemplified by Fig. S7 and instead indicate that the contacts used for the gap analysis operate in the spectroscopic regime.

Taken together, the theoretical expectations for thermal contacts, the experimental example of a spectrum in $MoTe_2$ in thermal regime, and the clear qualitative differences from the high-pressure data provide a more compelling basis to exclude point-contact heating as the origin of the single peak near zero bias.

The discussion has been added to the main text (page 14, line 2), and detailed explanations along with the additional experimental example are provided in Sec. S1.4.1 and Fig. S7 in the Supplementary Information.

- **Results, page 5, second sentence of the second paragraph:**
  One possible scenario is a heating-induced thermal regime, in which local Joule heating and critical-current effects can generate a broad, strongly field-sensitive anomaly [Fig. S7].

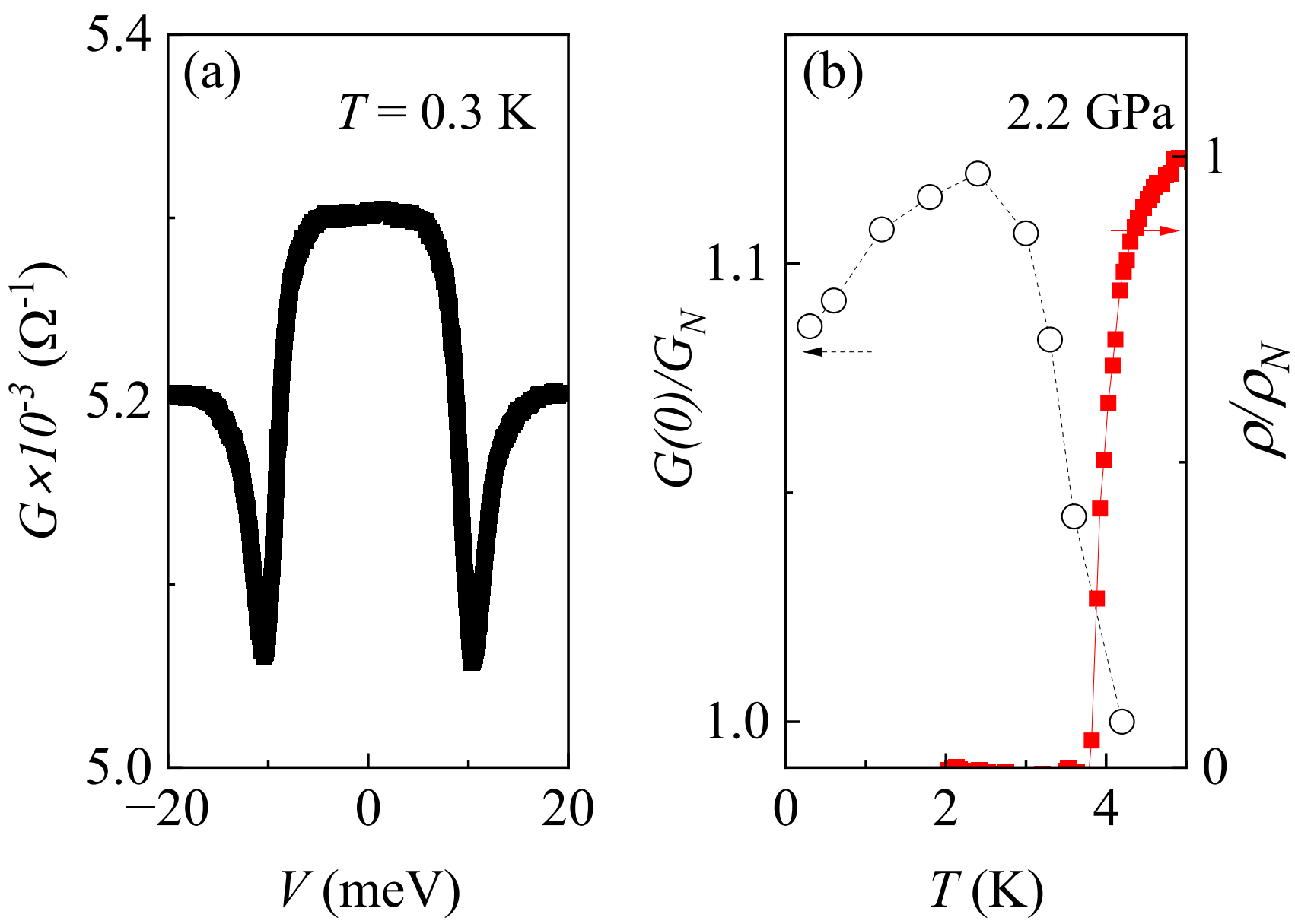

Fig. R8. [Revised Fig. S7] **(a)** Differential conductance $G(V)$ measured at $T = 0.3$ K for a point contact operating in the thermal (non-spectroscopic) regime. The spectrum exhibits a broad zero-bias conductance maximum with suppressed conductance at finite bias and no sharp Andreev-reflection features, consistent with transport dominated by local heating and critical-current effects rather than ballistic point-contact spectroscopy. **(b)** Zero-bias differential conductance $G(0)$ (open symbols) shown as a function of temperature (left ordinate), together with the electrical resistivity $\rho(T)$ (solid symbols, right ordinate) magnified near the superconducting phase transition.

The entire dataset appears to be derived from a single sample. Given the central claim regarding surface p-wave superconductivity, it is essential to demonstrate reproducibility. I strongly recommend that the authors provide data from at least one additional sample to rule out sample-specific or contact-dependent artifacts.

We appreciate the Referee's comment regarding sample reproducibility.

To address this point, we have added data from an independent point contact on a second crystal. As shown in the revised Fig. S9 below, measurements at 0.6 GPa and 1.6 GPa for this additional sample exhibit the same qualitative behaviour as in the main dataset: in the T_d phase at 0.6 GPa the spectra display only the double Andreev peaks expected for multi-gap s-wave superconductivity with no enhancement at zero bias, whereas in the 1T′ phase at 1.6 GPa a sharp single peak near zero bias develops between the two Andreev maxima.

The fact that this evolution is reproduced for a different contact on a separate crystal indicates that the central peak is an intrinsic property of the high-pressure 1T′ superconducting phase rather than a sample- or contact-specific artifact. The new data and discussion are presented in Supplementary Sec. S1.4.3 and Fig. S9.

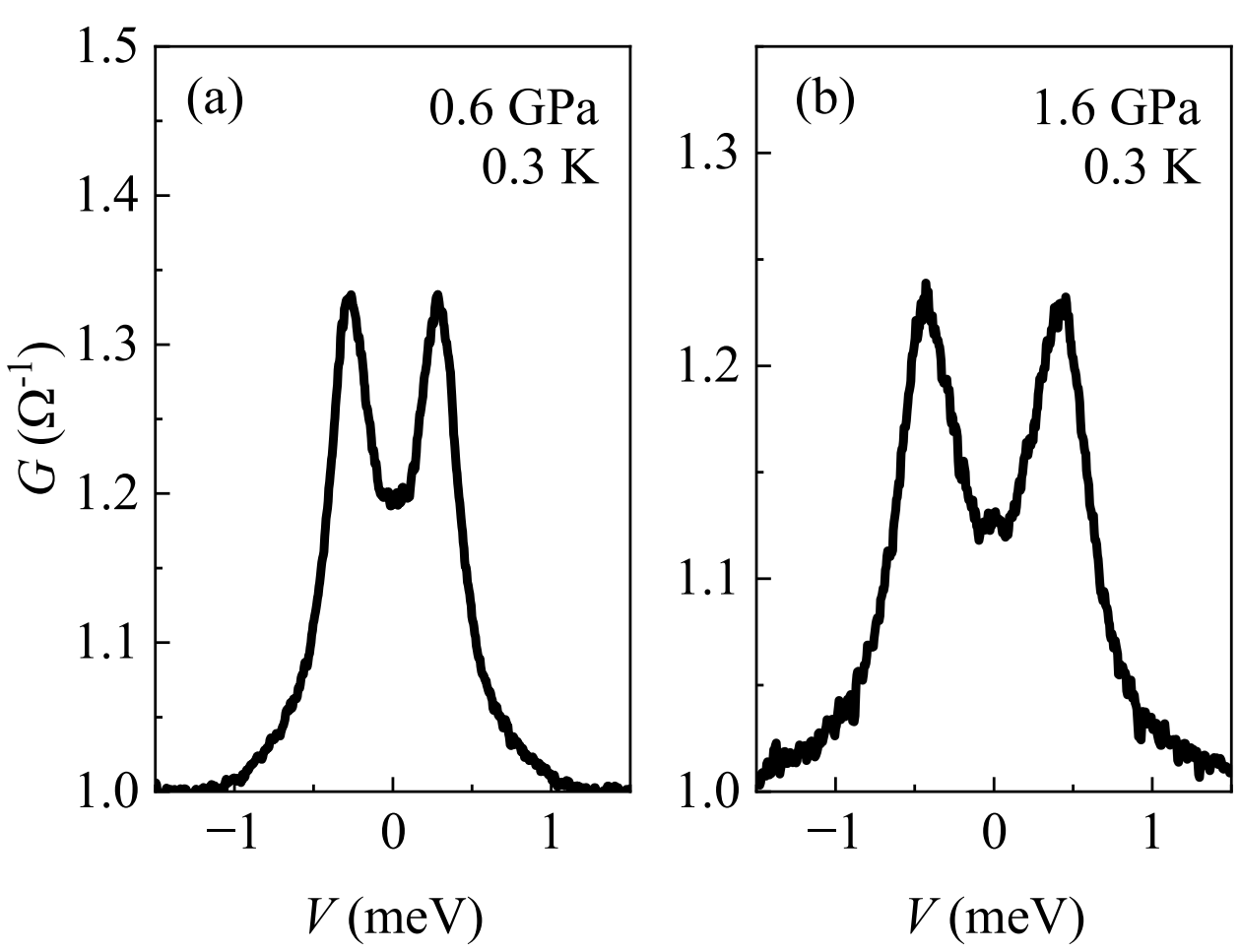

Fig. R9. [Revised Fig. S9] Evolution of the single near-zero-bias conductance peak between the double Andreev maxima for two different point contacts. **(a)** Spectrum at 0.6 GPa in the T_d phase, showing only the double Andreev peaks expected for multi-gap s-wave superconductivity and no enhancement at zero bias. **(b)** Spectrum at 1.6 GPa in the 1T′ phase, where a sharp zero-bias peak develops between the two Andreev peaks. The appearance of the central peak only after the pressure-driven phase transition from T_d to 1T′ transition supports its intrinsic origin in the high-pressure superconducting state.

The observed suppression of both s- and p-wave features under relatively modest magnetic fields is used to argue that the p-wave gap is induced via proximity to bulk s-wave superconductivity, not intrinsic. This is a reasonable interpretation and consistent with the theoretical model.

However, the fragility of the ZBCP under field also means that the associated topological protection (if any) is weak, and further experiments would be necessary to confirm the unconventional nature of the state.

We thank the Referee for this thoughtful comment and for recognizing that the field dependence of the s- and p-wave gaps is consistent with a proximity-induced p-wave component in the 1T′ phase.

1. Symmetry protection and field fragility

- In our microscopic description, the second-order topological insulator phase of $MoTe_2$ is protected by inversion ($\mathcal{P}$) and time-reversal ($\mathcal{T}$) symmetry. In the superconducting 1T′ phase, a bulk s-wave condensate coexists with inversion-protected helical boundary states; their proximity coupling generates an $s+p$ pairing channel and realizes a time-reversal-invariant higher-order topological superconductor at low fields.
- An applied magnetic field explicitly breaks $\mathcal{T}$ and enters the model as a Zeeman pair-breaking term, suppressing both the bulk s-wave gap and the induced p-wave component. The experimentally observed fragility of the single peak near zero bias with respect to field [Figs. R4(d-f), revised Figs. 4(d-f)] is therefore not in conflict with a symmetry-protected origin, but rather reflects the breaking of the protecting symmetry.

2. Consistency with the proximity-induced $s+p$ scenario

- Quantitatively, the PCAR spectra at 2.2 GPa are captured over the entire field range by a single two-gap $s+p$ BTK model [Figs. R4(d,e)], and the extracted gaps $\Delta_s(H)$ and $\Delta_p(H)$ decrease in tandem and vanish at a common pair-breaking field, in good agreement with the coupled bulk-surface $s+p$ scenario [Fig. R4(f)].
- This contrasts with expectations for an intrinsic spin-triplet p-wave superconductor, where the p-wave gap would typically be more robust against moderate fields, and supports our interpretation of the p-wave component as proximity-induced rather than independent.

3. About further experiment

- We fully agree with the Referee that, given this field fragility, any topological protection is necessarily weak at finite field and that additional phase-sensitive and spatially resolved experiments will be required to directly establish the unconventional and higher-order topological nature of the state.
- In the present work, we therefore refer to the 1T′ phase as a candidate higher-order topological superconductor and emphasize the consistency of our data with this proximity-induced $s+p$ framework, rather than claiming a definitive demonstration of topological protection.

The corresponding explanation has been incorporated into the main text.

- **Results, page 5, first paragraph:**

  Symmetry considerations provide a natural framework for the emergence of the p-wave component. The single peak near zero-bias observed in the inversion-symmetric 1T′ phase is absent in the inversion-symmetry-broken T_d phase [Fig. 3(a)]. Moreover, the p-wave component is rapidly suppressed by an applied magnetic field, indicating its sensitivity to time-reversal-symmetry breaking [Figs. 4(d–f)]. These trends suggest that the symmetry setting of the 1T′ phase—bulk inversion symmetry and time-reversal symmetry, combined with surface inversion breaking in the presence of strong spin–orbit coupling—is closely tied to the emergence of the p-wave response, consistent with the theoretical analysis presented below.

The theoretical analysis is technically sound and comprehensive, involving a BdG treatment of a SOTI band structure and demonstrating how a third-order topological superconducting state can emerge. However, no direct experimental evidence of hinge or corner-localized states is presented. The claim of Majorana corner states therefore remains speculative and should be framed accordingly—as a theoretical proposal rather than an experimental observation.

We thank the Referee for this valuable comment regarding the presentation of Majorana corner states. We fully agree that, in the absence of direct experimental evidence for hinge- or corner-localized modes, any statement about Majorana corner states should be framed as a theoretical prediction rather than an observation.

In the revised manuscript, we have modified the wording throughout the Abstract, Introduction, and Results. The discussion of Majorana corner modes is now explicitly identified as a BdG-based theoretical proposal on top of the experimentally inferred $s{+}p$-wave state, and we refer to the 1T$'$ phase as a candidate higher-order topological superconductor. We also explicitly state that our theory provides a consistent and predictive framework for the data, but does not constitute direct experimental evidence for the hinge or corner modes.

We believe these changes bring the theoretical claims into alignment with the current level of experimental support.

To reflect this, we have revised several sections as follows:

- **Abstract, the last sentence:**
  Finally, we further propose that the resulting higher-order hinge boundary channels provide a natural route toward potential zero-energy Majorana corner modes under the observed $s{+}p$-wave proximity pairing, suggesting $MoTe_2$ as an intrinsic, pressure-tunable platform for multi-gap and $s{+}p$-wave topological superconductivity.

- **Introduction, page 2-3, second to last sentence:**
  Furthermore, theoretical analysis based on a Bogoliubov–de Gennes (BdG) Hamiltonian suggests that the SOTI hinge states can provide a route to corner-localized Majorana zero modes under appropriate $s{+}p$-wave proximity pairing, pointing to a possible third-order topological superconducting (TOTSC) regime.

- **Results, page 7, first sentence of the last paragraph:**
  Taken together with the SOTI boundary structure, the induced s- and p-wave components provide a minimal route toward a TOTSC phase under suitable conditions.

While the manuscript presents intriguing results and offers a novel perspective on pressure-induced topological superconductivity in $MoTe_2$, I find that the central claims—particularly those concerning p-wave superconductivity and higher-order topology—are not yet supported by sufficiently direct experimental evidence. Although the observed zero-bias conductance peak (ZBCP) is suggestive, it is not unique to p-wave pairing, and no spatially resolved measurements of hinge or corner states are provided. The theoretical framework is rigorous, but remains predictive rather than demonstrative. I cannot support publication in its current form, though I believe the work may be suitable after substantial experimental reinforcement.

We thank the Referee for this thoughtful overall assessment and for emphasizing the need for stringent analysis regarding p-wave superconductivity and higher-order topology.

As detailed in our responses to the specific comments in the reply, we have clarified the scope of our claims so that the 1T$'$ phase is consistently presented as a candidate higher-order topological superconductor, strengthened the experimental basis for the $s+p$ wave interpretation through additional pressure-, temperature-, and field-dependent PCAR data and a more quantitative BTK comparison of candidate gap symmetries. Additionally, we made it explicit that the higher-order topological superconductivity scenario is a concrete theoretical proposal to be tested by future spatially resolved and phase-sensitive experiments rather than a definitive demonstration.

After addressing the concerns raised by the reviewer, we believe that the revised manuscript is substantially improved and is now better suited for publication in this journal.

List of Changes:

The manuscript has been diligently revised to be in line with our responses. Modifications related to the Referee comments are highlighted in the "main_highlighted.pdf" document.

1. Title:
   Changed to "Symmetry-Controlled Multi-gap Superconductivity and Higher-Order Topological Phases of $MoTe_2$".

2. Authors: No change.

3. Abstract:
   The abstract has been changed to account for the changes in the main text.

4. Main text:
   (a) We have included a discussion regarding the T_d phase and its multi-gap superconducting state.
   (b) A theoretical discussion regarding the phase transition between the superconducting T_d and $1T'$ phase is also included in accordance with Referee 1's comments.
   (c) We have changed the tone of the manuscript to be more mild and suggestive regarding the emergence of Majorana states in accordance with Referee 3's comments.

5. Figures:
   (a) Fig. 1 has been updated to show characteristics of the T_d and $1T'$ phases in their normal and superconducting states.
   (b) A new Fig. 2 has been included, which shows the multi-gap nature of the T_d phase with theoretical comparisons that agree with the experimental findings.
   (c) Fig. 2 has been updated to compare the $s{+}p$-wave pairing to other potential candidates and show that this pairing best explains the results. This figure is now Fig. 3.
   (d) Fig. 3 has been updated to also show magnetic field dependence and a theoretical comparison is also provided. This figure is now Fig. 4.
   (e) Fig. 4 has been updated to show the second-order topological insulating state of the normal $1T'$ phase and been pushed to Fig. 5.

6. References:
   We have updated and corrected the reference list to reflect the revisions made to the manuscript and the points raised in the Referees' reports.

7. Supplementary Information:

   (a) We have included experimental results for alternative cases requested by Referee 3 in Secs. S1.2.4 and S1.2.5.

   (b) We discuss and dismiss other possible causes for the conductance peak at zero-conductance in Sec. S1.4.

   (c) Theoretical calculations for the T_d phase and its multi-gap characteristics are discussed in Sec. S2.
